# Distributional labour challenges and opportunities for decarbonizing the US power system

**DOI:** 10.1038/s41558-023-01802-5

**Published:** 2023-11-02

**Authors:** Judy Jingwei Xie, Melissa Martin, Joeri Rogelj, Iain Staffell

**Affiliations:** 1 Centre for Environmental Policy, Imperial College London, London, UK; 2 Grantham Institute on Climate Change and the Environment, Imperial College London, London, UK; 3 Element Energy (an ERM Group company), London, UK; 4 Energy, Climate, and Environment Program, International Institute for Applied Systems Analysis, Laxenburg, Austria

**Keywords:** Energy justice, Energy policy, Energy modelling, Climate-change mitigation, Energy economics

## Abstract

The transition towards a low-carbon power system presents challenges and opportunities for the workforce with important implications for just transitions. Studies of these distributional labour impacts could benefit from tighter linkages between energy and employment modelling. Here, we couple a power-sector optimization model, an employment impact model and demographic databases to understand state-level job characteristics and the societal implications of low-carbon transitions in the US. Although decarbonization brings consistent job growth, it heightens the need for investment in human capital and supply chain restructuring. Major fossil fuel-producing states need to prepare for fewer mining jobs under the US Long-Term Strategy, so other opportunities should be created or seized. The lowest-skilled workers will experience more uncertain employment outcomes. Expanding renewable energy could improve opportunities for women in fossil fuel-dependent states, but not enough to disrupt the national gender status quo. This work provides a new quantitative perspective to inform proactive just transition policies.

## Abstract (web summary)

The low-carbon transition would bring both challenges and opportunities for the labour market, which have important implications for a just transition. In the US power system, the lowest-skilled workers in fossil fuel-dependent states will face more uncertain employment and job training will be necessary.

## Main

The green economy sectors in the United States (US) employ more people and generate the highest revenues compared to their counterparts in other countries within the Organisation for Economic Co-operation and Development^1^. Simultaneously, being the country with most fossil fuel assets globally, the US is also exposed to high financial risks through low-carbon transitions^2^. Expanding the clean economy is crucial if the US is to remain competitive internationally and if it is to boost economic growth during downturns^3^. In addition, the US Long-Term Strategy stresses the necessity of reaching net-zero electricity by 2035^4^. The societal impacts of such environmental policies, however, are complex. For example, the 1990 Clean Air Act may have led to $5.9 billion in foregone earnings due to reduced employment^5^, although the resulting improvements to air quality may have boosted long-term labour market outcomes^6^. A discourse where climate policies are portrayed as killing jobs could hinder the political ambition, especially if distributional impacts are not managed^7^. Presently, it is estimated that the 2022 Inflation Reduction Act^8^ will create over 5 million clean-energy job-years in the next decade^9^ and will reduce the gap between current policy projections and the aim of halving emissions by 2030 from 2005 levels by 22–45% (ref. ^10^). To achieve the full benefits of the transition, states must plan for the unemployment, retraining and relocation costs due to the declining fossil fuel workforce to avoid a persistent fall in living standards^11^.

Utilizing the low-carbon transition for job growth in the US^11,12^ and globally^13–15^ is not new. The just transition concept arose within the labour union movement in the 1980s in response to the impact of environmental regulations on employment in North America^16^. It now encompasses distributional, recognition, procedural, restorative and cosmopolitan justice^17–19^. The concept rose to prominence in climate change policy discourse after the 2015 Paris Agreement^20^. Its goals have since broadened from creating ‘decent work and quality jobs’^20^ to highlighting the importance of inclusivity and social dialogues, as described in the 2018 Silesia Declaration^21,22^. The just transition will produce both labour opportunities and challenges^23^. Although the fossil fuel workforce is expected to decline, in a Paris-aligned world, the introduction of low-carbon technologies would create manufacturing and construction jobs^13–15^. However, the local impacts of such transitions are sensitive to political, industrial and technological factors^24^.

Given the impactful roles of subnational climate actors^25^ and the cost-effective potential for state-driven strategies^26^, geographically granular analyses are needed to produce relevant insights for those actions. The Net Zero America report highlighted the overall growth in solar, wind and grid jobs^27^. Retraining coal workers for solar jobs is both feasible and financially manageable^28^. Solar also has more resource availability to provide local jobs in coal mining regions^29^. Expanding wind energy in states with large existing capacities could create substantial economic impacts locally and through spillovers into other states^30^. These employment impacts will not be distributed equally across states^27^ and neither will be the relevant local social policies^18^. Uncertainties around the future energy system, especially relating to natural gas prices^31^ and geopolitical tensions^32^, will further complicate the problem.

Ex ante studies of US subnational employment impacts typically use computable general equilibrium models^33^, input–output models^11,30,34^ or analytical methods^35^. They are linked to energy-system models that incorporate emissions reductions and some system sensitivities (for example, electrification)^34^ or are based on specific policy instruments^30,33,35^. Other efforts that explore social justice implications often provide qualitative^36^ or ex post evaluations^37^. Considering the pace of change required to achieve the target of net-zero electricity by 2035^4,38^, a quantitative, ex ante and granular understanding of state-level distributional impacts in the uncertain future would improve institutional readiness to leverage the transition.

## Results

**Fig. 1:**
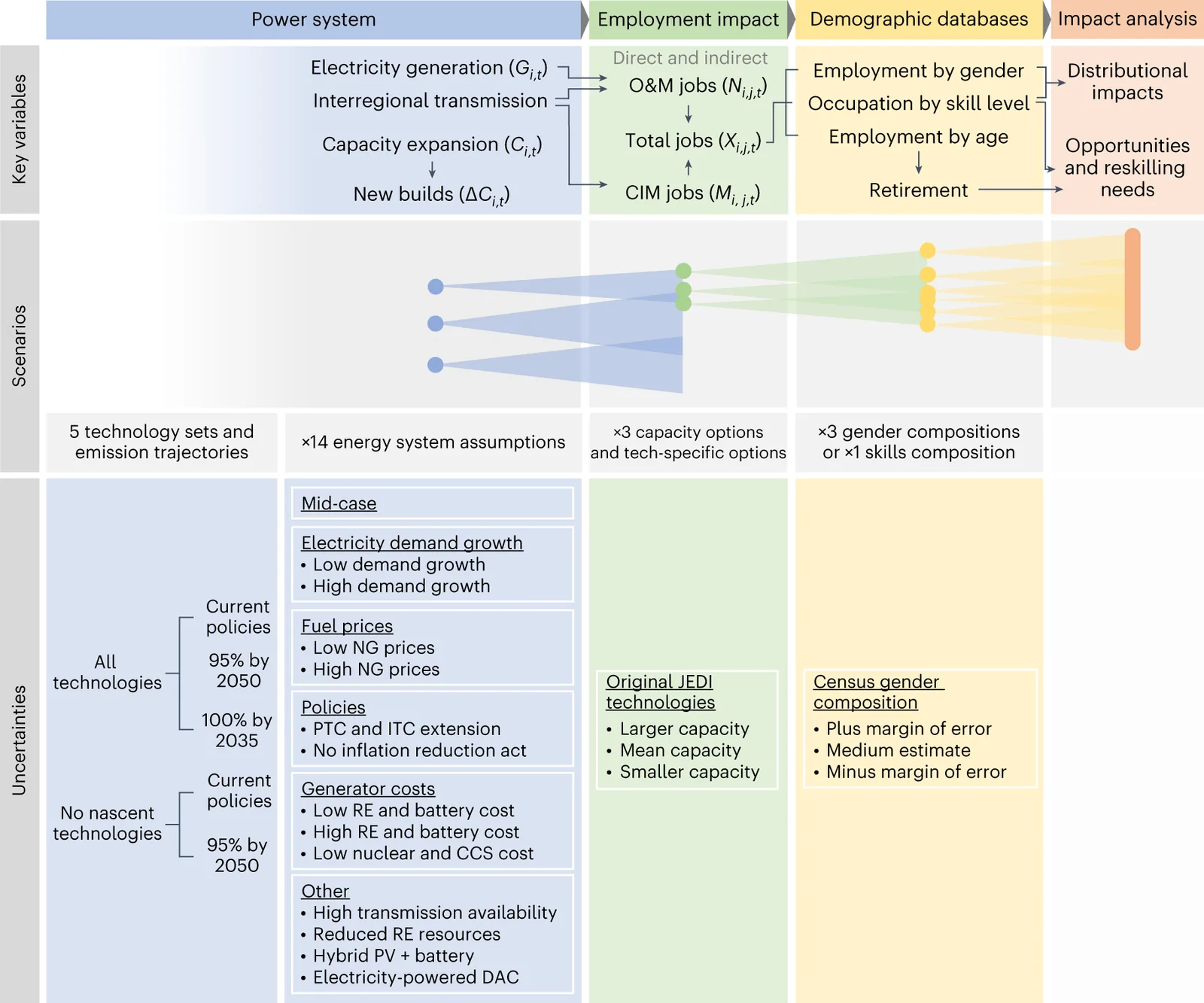
Schematics of the model and analysis workflow for evaluating the distributional employment impacts of power-system transition scenarios. The supply-side power-system parameters include the installed capacity (*C*) of electricity generators in MW, annual electricity generation (and consumption by DAC in its sensitivity case) (*G*) in MWh and the interregional transmission lengths and capacities reported by ReEDS^41^. The distribution system is not modelled by ReEDS and, thus, is not included in this analysis. These parameters are passed to the JEDI models^40^, which calculate the operation and maintenance (O&M), construction, installment, and manufacturing (CIM), and total jobs (*M*, *N* and *X*), as a function of year and sector. Both direct and indirect jobs are included in units of full-time jobs. This framework accounts for various uncertainties covering (1) three emission trajectories and two cases excluding nascent technologies^41^ such as CCS, small modular nuclear reactors and floating offshore wind farms, (2) 14 energy-system assumptions including a mid-case and 13 sensitivities, (3) three main capacity multiplier options and (4) three gender compositions. The mid-case energy-system assumption uses central or median values of technology or fuel input parameters and assumes demand will grow by 1.3% per year^41^. The other sensitivity cases explore varied demand growth, generator costs and fuel prices. The emission trajectory for current policies includes electricity-sector policies as of September 2022 (including the Inflation Reduction Act)^41^. *i*, technology; *t*, year; *j*, economic sector; ITC, Investment Tax Credit; NG, natural gas; PTC, Production Tax Credit; RE, renewable energy.

We systematically evaluated the transition of the US power-sector workforce by coupling the 2022 Regional Energy Deployment System (ReEDS)^39^ (a state-level power-sector optimization model) with the Jobs and Economic Development Impact (JEDI) input–output models^40^ and demographic databases. Acknowledging the wide uncertainties in the future energy system^31^, we estimated the scale and pace of the workforce transition under 70 scenarios (Fig. 1). We contextualized our findings against historical technology-led workforce mobilization events and disaggregated workforce needs by state, economic sector and skill level. We discuss how the changing employment landscape may impact local community demographics and lead to changing social dynamics. From these results, we derived several policy implications relevant to just transition planning at the local and federal levels and suggest future research directions.

### Scale and pace of the workforce transition

We found that more stringent power-sector emission reductions will not require substantial labour expansions nationally compared to current policies, which include the ambitious efforts of the Inflation Reduction Act. Current policies with mid-case assumptions would lead to 77% job growth in the national power sector (66–94% minimum/maximum spread across all energy-system assumptions) and 77% emission reduction (46–87%)^41^. The employment outcome is equivalent to 458 (384–577) thousand new jobs in the electricity system in the 2040s, although some efforts will be front-loaded in the 2030s (Supplementary Section 1). Reducing emissions by 95% by 2050 would lead to statistically insignificant differences (*P* = 0.77), but the same trajectory would lead to 5.6% fewer jobs (*P* = 0.01) without nascent technologies. Achieving net zero by 2035 would result in 439 (347–554) thousand new jobs, which is 0.98 ± 0.05 times (*P* = 0.40) the number of jobs under current policies (Supplementary Section 7.1.1), indicating that there are negligible additional impacts compared to existing policies and consistent national employment growth in the power sector.

**Fig. 2:**
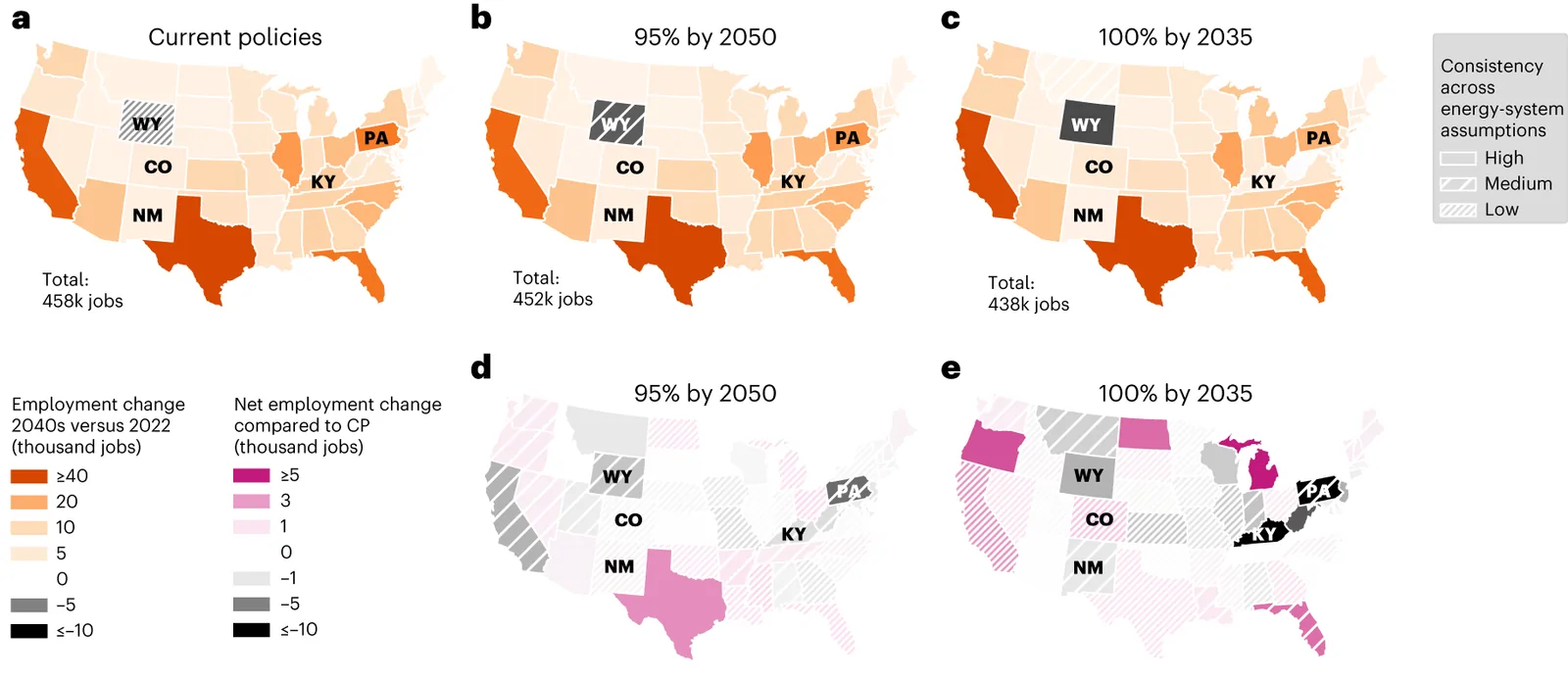
Modelled employment change and net employment change under the mid-case emission-reduction scenarios in the 2040s. Employment change (equation (7)) describes the difference of the workforce in the decade before mid-century compared to today while accounting for natural retirement. A value of 1 indicates that 1,000 new entrants would be required to deliver the transition. A value of −5 indicates that 5,000 current workers may lose their jobs in addition to natural retirement. Net employment change (equation (8)) is the employment change of an emission-reduction trajectory compared to that of the current policies trajectory. A value of 1 means that said emission trajectory creates 1,000 more jobs than current policies in the 2040s. **a**–**c**,The top row of maps shows the employment change under current policies (**a**), 95% reduction by 2050 (**b**) and 100% reduction by 2035 (**c**). States coloured orange see employment growth, whereas grey states see employment decline. **d**–**e**, The bottom row of maps shows the net employment change under 95% reduction by 2050 (**d**) and 100% reduction by 2035 (**e**) compared to current policies. States coloured pink see more employment under decarbonization than CP, whereas grey states see less. Hatching denotes the level of consistency across energy-system assumptions within each group. High consistency means all energy-system assumptions have the same sign of change as the mid-case. Medium and low consistencies represent more than and less than 80% of the 14 energy-system assumptions sharing the same sign as the mid-case, respectively. The base maps are from the US Census Bureau^85^. CP, current policies.

**Extended Data Fig. 1:**
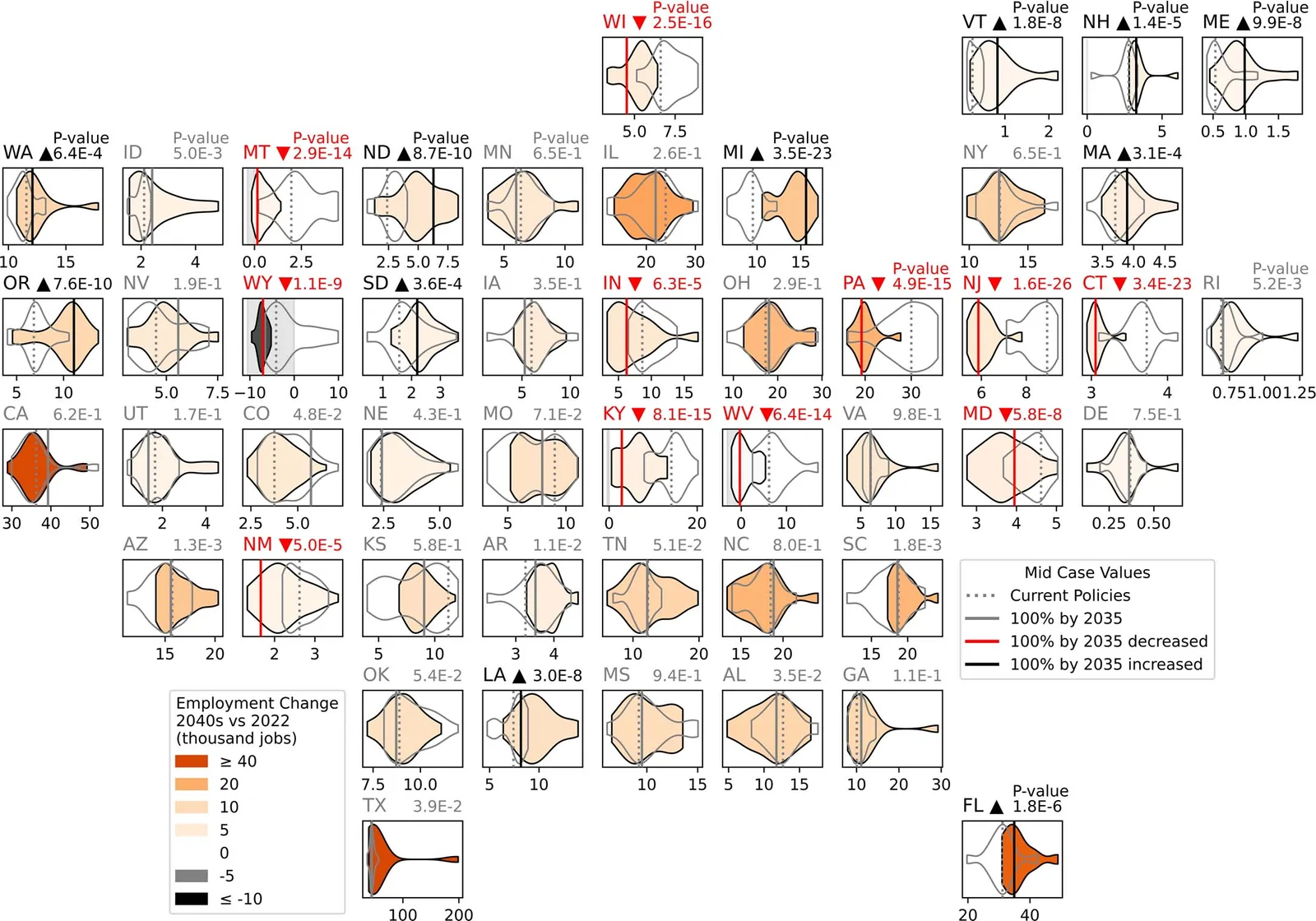
State-level Employment Change under the 100% by 2035 emission trajectory. Employment Change (Eq. 7) describes the difference in the workforce in the decade before mid-century compared to today while accounting for natural retirement. The empty violin plots (minima to maxima) with grey outlines show results from all systems assumptions under Current Policies (n = 39); the dotted grey lines represent the mid-case system assumption. The filled violin plots represent all results from the 100% by 2035 trajectory (n = 39); the solid lines represent their mid-case. Solid grey line mean the emission reductions show no significant difference from Current Policies (P > 0.001) across system assumptions. Solid red lines indicate that the emission reduction trajectories lead to significantly fewer jobs (P < 0.001); solid black lines indicate significantly more jobs (P < 0.001). The state labels are marked accordingly with black upward and red downward arrows. The P values to the right of state labels are calculated from one-sided Welch’s t-tests. The negative areas are shaded grey.

**Extended Data Fig. 2:**
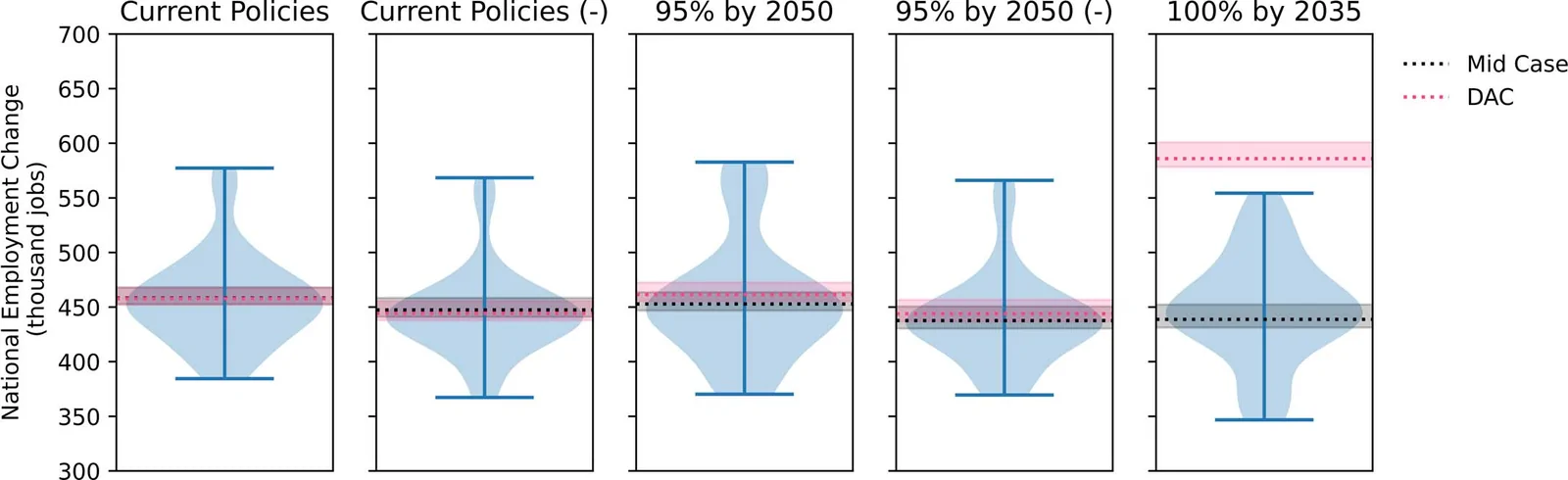
Modelled national Employment Change across all emission reduction trajectories and energy system assumptions in the 2040 s compared to the 2020 s. Employment Change (Eq. 7) values indicate the size of new opportunities created in the 2040 s compared to the 2020 s state-level electricity workforce, while accounting for natural retirement. The violin plots illustrate the distribution of outcomes across all modelled scenarios (n = 39) in each emission trajectory, which appears to be bimodal. The blue horizontal lines show the maxima and minima. The shaded areas indicate scenarios (n = 3) under the mid-case (black) and DAC (pink) energy system assumptions; the dashed lines indicate the scenario under the medium multiplier option. The mid-case scenarios occur in the main peaks. The DAC scenarios occur above the other scenarios only under the 100% by 2035 trajectory.

**Extended Data Fig. 3:**
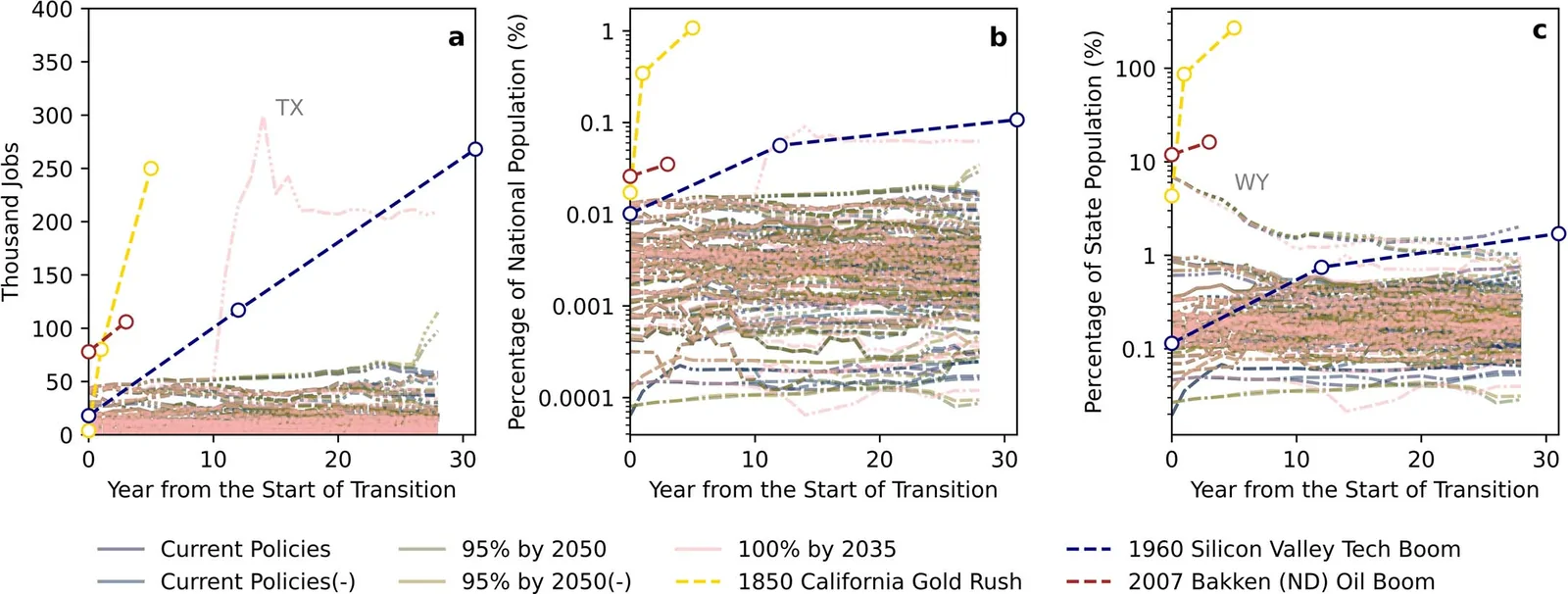
Modelled state-level employment growth under the DAC sensitivity compared to historical workforce mobilization events in energy and technology in the US. The modelled results are shown over the planning horizon of 2022–2050 for power system emission trajectories including Current Policies, CO2 emissions targets of a 95% reduction by 2050 with and without nascent technologies, the latter denoted with (−) and 100% reduction by 2035. Panel (**a**) shows the absolute employment growth, (**b**) shows the percentage of the total US national population mobilized as workforce at the start of the transition, and (**c**) shows the percentage of the state population mobilized as workforce at the start of the transition. The emission trajectory colour scheme is based on the Scientific Colour Map^86^. TX, Texas; WY, Wyoming.

Across emission trajectories under the mid-case assumption, power-sector jobs are likely to grow in all states apart from Wyoming (Fig. 2). Montana and West Virginia may also need to prepare for fewer electricity jobs under the US Long-Term Strategy (Extended Data Fig. 1). Generally, coastal states see more opportunities with high consistency across assumptions. In line with other works in the literature, West South Central and South Atlantic states are notable winners in the transition^33^. Under the net zero by 2035 trajectory, Texas’s workforce will grow from 48.6 ± 1.5 thousand jobs in the 2020s to 61.6 ± 4.4 in the 2040s, showing the largest growth due to the magnitude of its electricity system. Furthermore, competitive electricity prices and carbon transport and storage costs^41^ mean that Texas would see enough new electricity jobs for 1% of its current population if direct air capture (DAC) is deployed (Extended Data Figs. 2 and 3). Similarly, California would benefit from the scale of its electricity system and would have sizeable growth in wind and utility-scale photovoltaics (PV). Florida would see major growth of utility-scale PV jobs, which supply most of the state’s future increased demand for energy. Although Wyoming’s power sector employs 5% of the state’s workforce (Fig. 2), it could experience job losses from the declining coal supply chain across scenarios. Current policies would reduce its workforce from 23.6 ± 3.5 thousand jobs in the 2020 s to 11.3 ± 5.1 thousand jobs in the 2040s.

**Extended Data Fig. 4:**
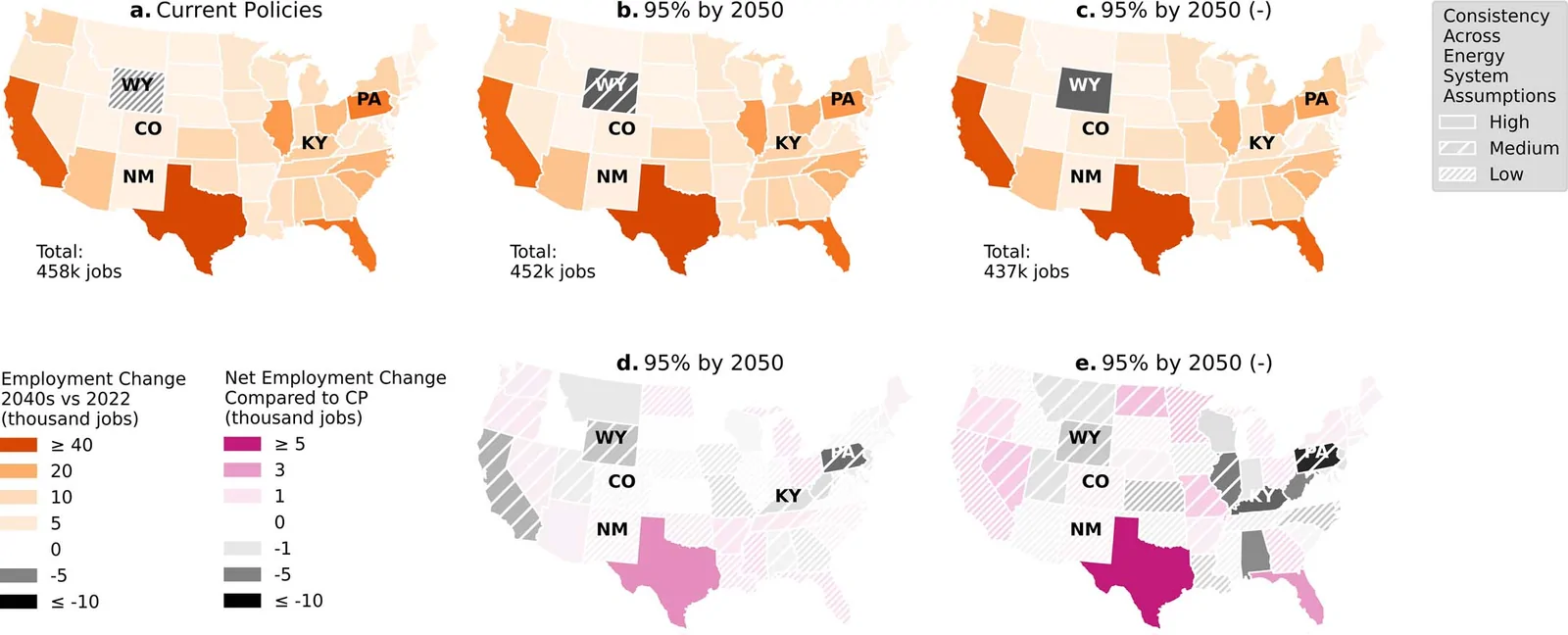
Modelled Employment Change and Net Employment Change under the mid-case emission reduction scenarios with and without nascent technologies in the 2040 s. Employment Change (Eq. 7) describes the difference of the workforce in the decade before mid-century compared to today while accounting for natural retirement. A value of 1 indicates that 1000 new entrants would be required to deliver the transition. A value of -5 indicates that 5000 current workers may experience job loss in addition to natural retirement. Net Employment Change (Eq. 8) indicates the Employment Change of an emission reduction trajectory compared to that of the Current Policies trajectory. A value of 1 means that said emission trajectory creates 1000 more jobs than Current Policies in the 2040 s. The top panel show the Employment Change under (**a**) Current Policies, (**b**) 95% reduction by 2050 with nascent technologies, and (**c**) 95% reduction by 2050 without nascent technologies. States coloured orange see employment growth, while grey states see employment decline. The bottom panel maps show the Net Employment Change under (**d**) 95% reduction by 2050 with nascent technologies and (**e**) 95% reduction by 2050 without nascent technologies compared to Current Policies (CP). States coloured pink see more employment under decarbonization than CP, while grey states see less. Hatching denotes the level of consistency across energy system assumptions within each group. High consistency means all energy system assumptions have the same sign of change as the mid-case. Medium and low consistencies represent more than and less than 80% of the 14 energy system assumptions sharing the same sign as the mid-case, respectively. The base maps are from the US Census Bureau^85^.

Despite consistent job growth across emission trajectories, rapid decarbonization would have winners and losers geographically. The disparity across states is intensified with more stringent emission-reduction targets (Fig. 2) and without available nascent technologies (Extended Data Fig. 4). Understandably, states with a heavy fossil fuel dependency (Appalachia) would experience less net employment growth in more decarbonized futures. With tighter emission constraints, Pennsylvania and Kentucky would show lower employment growth as the utilization of their natural gas assets falls and the assets become stranded. Despite this, the power-sector workforce in these states would expect new entrants, as retirements will outweigh job losses. Most states with lowered job growth under decarbonization scenarios show low consistency between energy-system assumptions, meaning that greater employment than with current policies is possible depending on how the low-carbon transition is designed. For example, the 100% by 2035 trajectory with low renewable energy costs could introduce ~1,000 more jobs in New Mexico through onshore wind, in contrast to the fewer jobs under other system assumptions (Extended Data Fig. 1). Conversely, Colorado would benefit from rapid decarbonization in the mid-case but could experience less employment growth under low natural gas prices and high renewable energy costs as its onshore wind becomes less competitive.

**Fig. 3:**
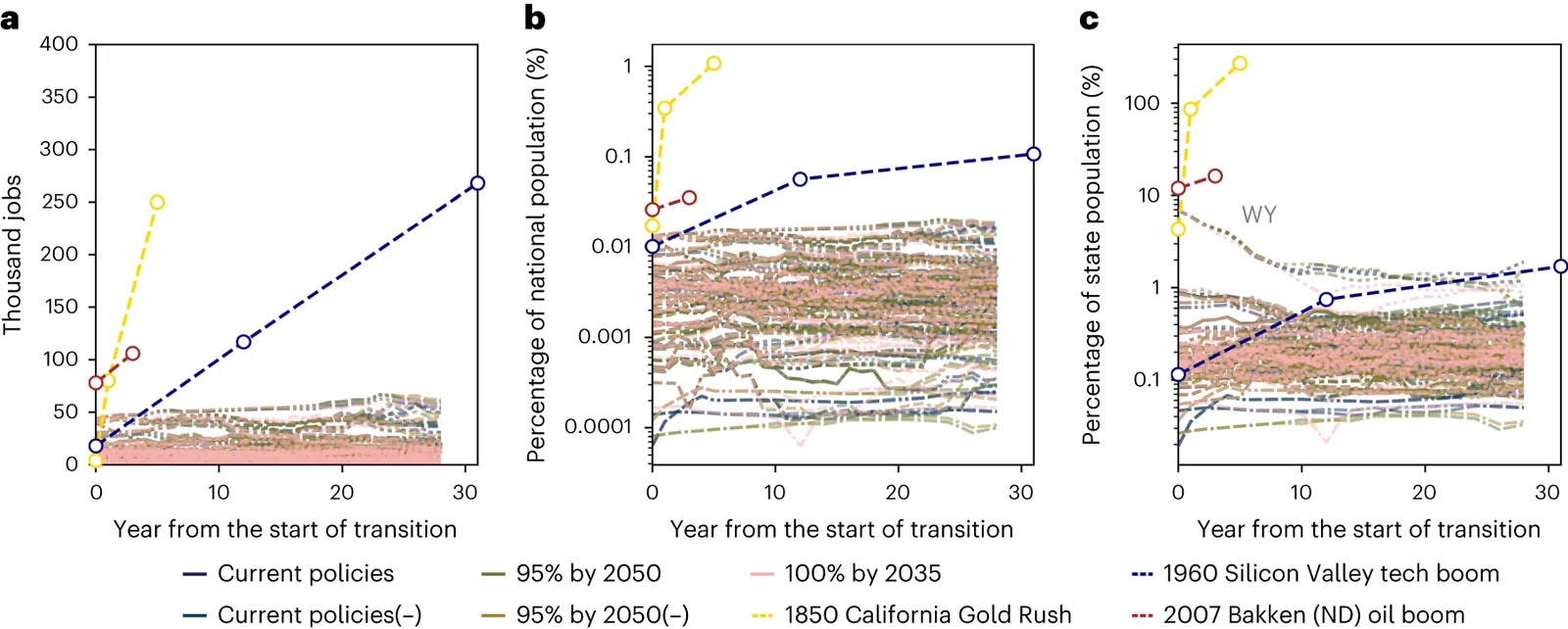
Modelled state-level employment growth under mid-case energy-system assumptions compared to historical workforce mobilization events in energy and technology in the US. The modelled results are shown over the planning horizon of 2022–2050 for power-system emission trajectories including current policies, CO_2_ emissions targets of a 95% reduction by 2050 with and without nascent technologies, the latter denoted with (−) and 100% reduction by 2035. **a**, Absolute growth of employment. **b**, Percentage of the total US national population mobilized as workforce at the start of the transition. **c**, Percentage of the state population mobilized as workforce at the start of the transition. The colour scheme for the emission trajectories is based on the Scientific Colour Maps^86^. WY, Wyoming.

We contextualized the feasibility of the scale and pace of this workforce mobilization with historical energy and technology transitions. The growth of state-level employment and their values as percentages of the national and state workforce^42^ are generally lower than those in the 1850 California Gold Rush^43^, the 1960 Silicon Valley tech boom^44^ and the 2007 North Dakota oil boom^45^ (Fig. 3). The pace of workforce mobilization required to reach net zero by 2035 does not exceed historical high-tech and lower-skilled^46^ labour shocks, although those shocks were more localized than the wide-scale electricity-system transition. The modelled employment in the state-level electricity system is on a par with those reported by the Department of Energy^47^ (Supplementary Section 7.2).

### Future sectoral and skill-level training needs

Technology-wise, solar will play a substantial role in electricity-system employment by mid-century. The expansion of employment in the onshore wind, transmission, batteries and hydro-energy sectors will be especially important to delivering net zero by 2035. This trajectory would also see a steady decline in oil power generation jobs and a shift from natural gas jobs to those with postcombustion capture (Supplementary Table 36). These results are in line with those in the literature^33^.

**Fig. 4:**
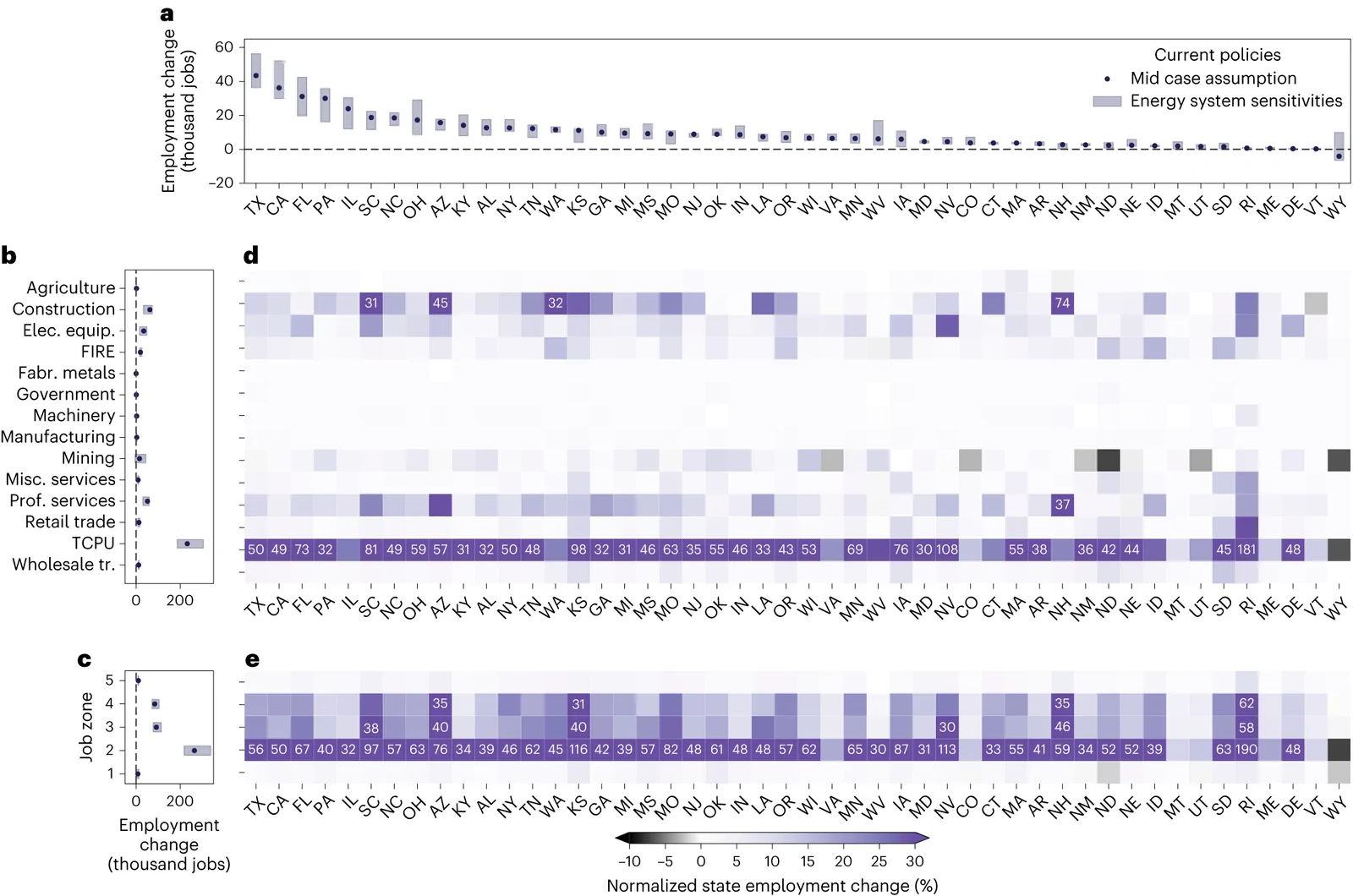
Distribution of employment change under current policies between the 2020s and 2040s. The change in employment is shown across states (horizontally), economic sectors and job zones (vertically) in terms of absolute number of jobs (blue bar charts) and normalized to the existing size of the electricity-sector workforce (purple/grey heat maps). **a**–**c**, The bar graphs show the employment change in thousands of jobs (equation (7)) for the 2040s compared to the 2020s, aggregated by state (**a**), economic sector (**b**) and job zone (**c**) across all power-sector transition scenarios. A value of 1 indicates that 1,000 new entrants would be required to deliver the transition. A value of −5 indicates that 5,000 current workers may lose their job in addition to natural retirement. The circles represent the mid-case, and the transparent bars represent the full suite of energy-system assumptions. States are sorted in descending order of their state-level employment growth. **d**–**e**, The heat maps represent the employment change normalized to the state’s electricity-system job numbers (as a percentage) in 2022 in the mid-case for economic sector (**d**) and job zone (**e**). Normalized state employment changes (equation (9)) show the magnitudes of employment growth (purple colour scheme) and job losses (grey colour scheme). The numbers in white indicate where the normalized state employment changes for subsets of the workforce (state and sector or job zone) grow by more than 30% of the current workforce. A value of 50% would indicate that the number of additional workers required to deliver the transition in a given state and sector (for example, agriculture in California) is equivalent to 50% of that state’s current electricity workforce. Miscellaneous services include sectors such as education, healthcare and the arts. Elec. equip., electrical equipment; Fabr. metals, fabricated metals; FIRE, finance, insurance and real estate; Misc., miscellaneous; Prof., professional; TCPU, transportation, communications and public utilities; Wholesale tr., Wholesale trade. The x-axis labels of **a**, **d**, and **e** are two-letter abbreviations of states within the contiguous United States.

Driven by the technology transition, employment impacts also vary across state and sector. Under current policies, the utilities sector shows the most growth due to the nature of the electricity system (Fig. 4). As with works in the literature^13,15^, we found that construction, professional services and electrical equipment exhibit the next highest employment growth, with 60.4 ± 9.2, 49.0 ± 7.7 and 34.6 ± 8.9 thousand new jobs, respectively. Some states benefit from technology expansion more than others, notably onshore wind (Texas), offshore wind (California), solar (California, Florida and South Carolina) and nuclear (Texas, Pennsylvania and Illinois). Other states fall behind due to the decline of coal (Wyoming and North Dakota) and mining jobs (Fig. 4). Unsurprisingly, Wyoming may avoid job losses if the costs of nuclear and carbon capture and storage (CCS) are low.

**Extended Data Fig. 5:**
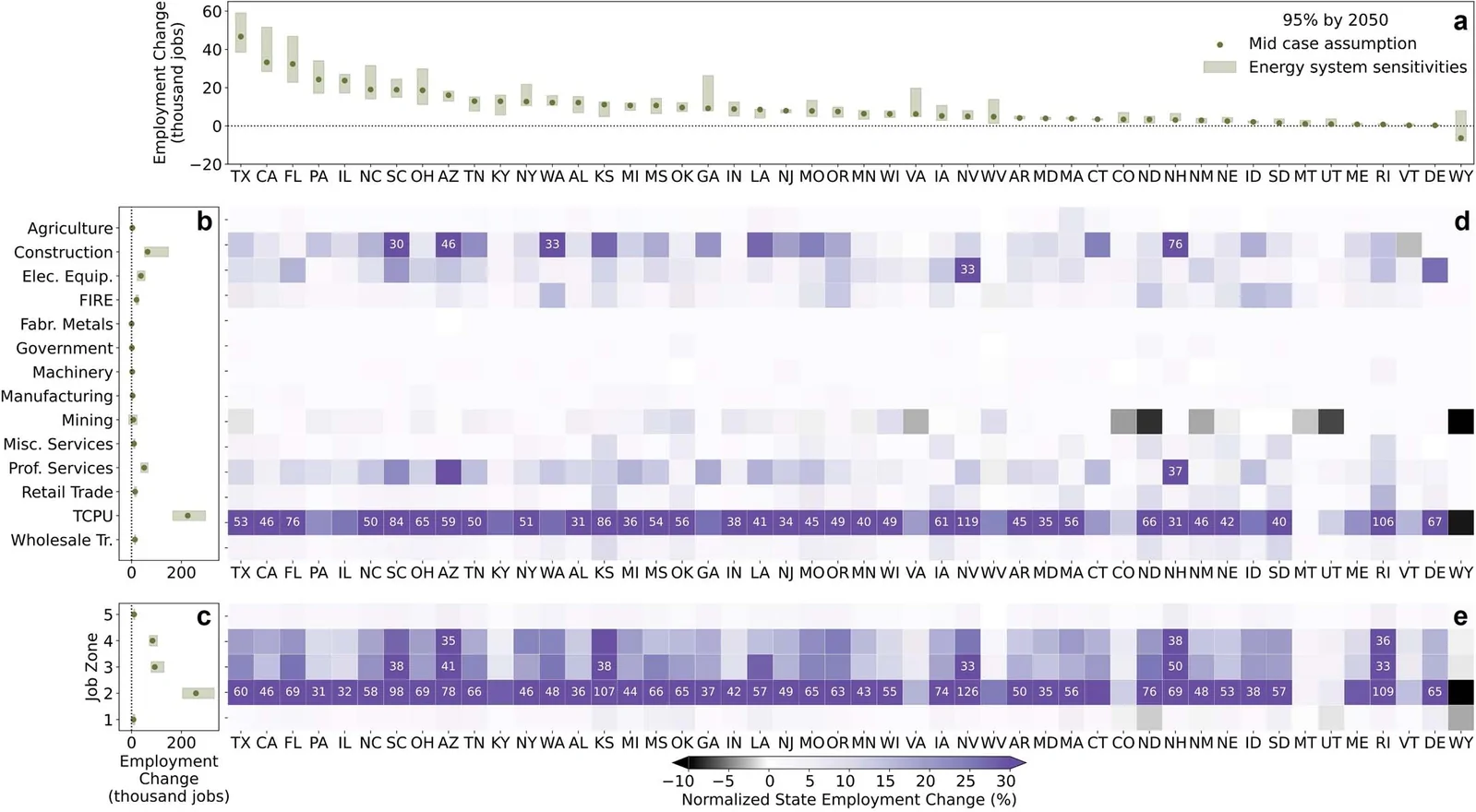
Distribution of Employment Change in the 95% by 2050 trajectory between the 2020s and 2040s. The change in employment is shown across states (horizontally), economic sectors and Job Zones (vertically) in terms of absolute number of jobs (blue bar charts) and normalized to existing size of the electricity sector workforce (purple/grey heatmaps). The bar graphs show the Employment Change in thousands of jobs (Eq. 7) of the 2040s compared to the 2020s aggregated by state (a), economic sector (b), and Job Zone (c) across all power sector transition scenarios. A value of 1 indicates that 1000 new entrants would be required to deliver the transition. A value of -5 indicates that 5000 current workers may experience job loss in addition to natural retirement. In the top panel, the circles represent the mid-case, and the transparent bars represent the full suite of energy system assumptions. States are sorted in descending order of their state-level employment growth. Heatmaps (d-e) represent the Employment Change normalized to the state’s electricity system job numbers (in percentage) in 2022 in the mid-case. The Normalized State Employment Changes (Eq. 9) show the magnitudes of employment growth (purple colour scheme) and job losses (grey colour scheme). The white texts indicate the Normalized State Employment Changes in subsets of the workforce (state and sector or job zone) growing more than 30% of the current workforce. A value of 50% would indicate that the number of additional workers required to deliver the transition in a given state and sector (for example, agriculture in California) is equivalent to 50% of that state’s current electricity workforce. TCPU stands for Transportation, Communications, and Public Utilities. FIRE stands for Finance, Insurance, and Real Estate. Miscellaneous services include sectors such as education, healthcare, and the arts.

**Extended Data Fig. 6:**
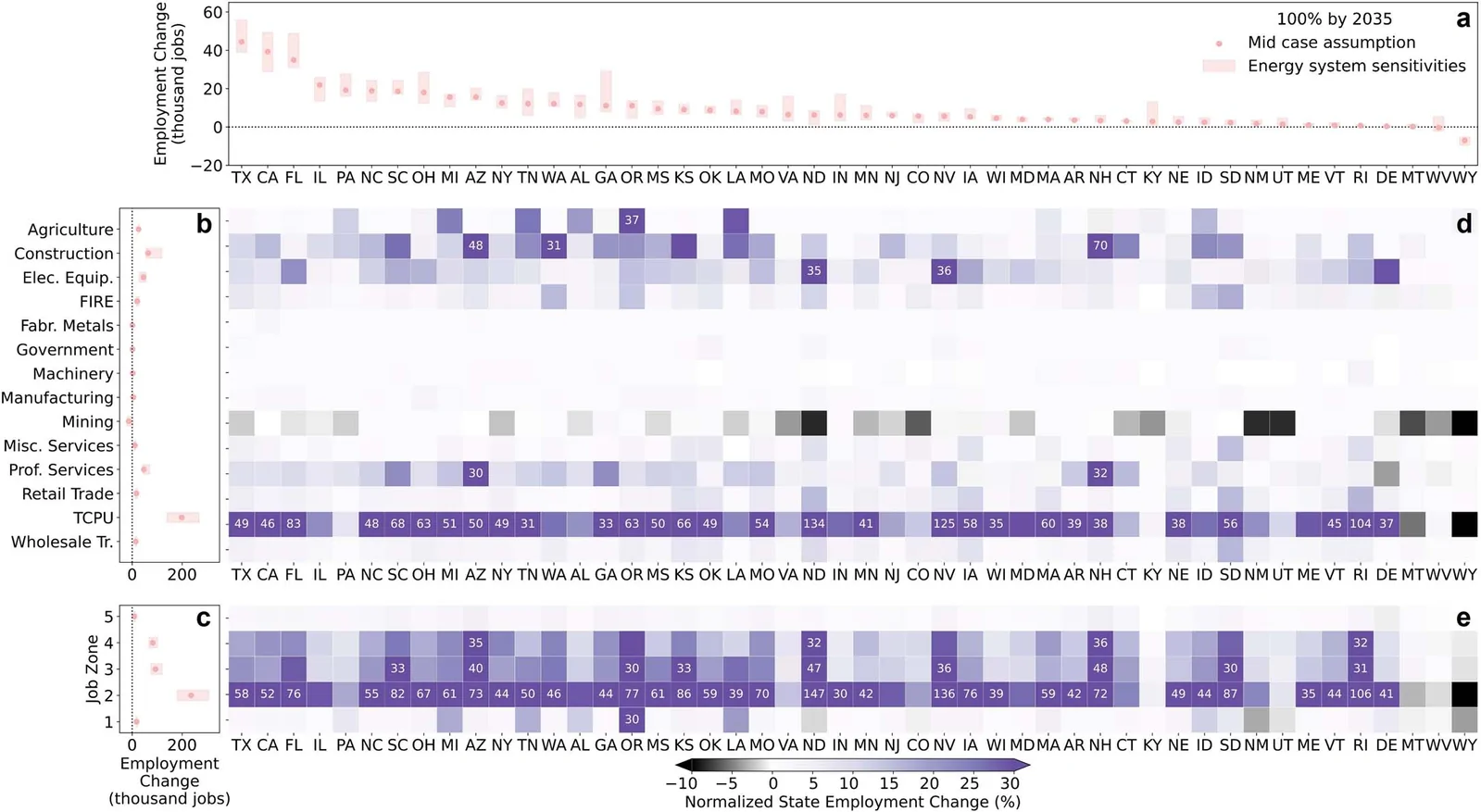
Distribution of Employment Change in the 100% by 2035 trajectory between the 2020s and 2040s. The change in employment is shown across states (horizontally), economic sectors and Job Zones (vertically) in terms of absolute number of jobs (blue bar charts) and normalized to existing size of the electricity sector workforce (purple/grey heatmaps). The bar graphs show the Employment Change in thousands of jobs (Eq. 7) of the 2040s compared to the 2020s aggregated by state (a), economic sector (b), and Job Zone (c) across all power sector transition scenarios. A value of 1 indicates that 1000 new entrants would be required to deliver the transition. A value of -5 indicates that 5000 current workers may experience job loss in addition to natural retirement. In the top panel, the circles represent the mid-case, and the transparent bars represent the full suite of energy system assumptions. States are sorted in descending order of their state-level employment growth. Heatmaps (d-e) represent the Employment Change normalized to the state’s electricity system job numbers (in percentage) in 2022 in the mid-case. The Normalized State Employment Changes (Eq. 9) show the magnitudes of employment growth (purple colour scheme) and job losses (grey colour scheme). The white texts indicate the Normalized State Employment Changes in subsets of the workforce (state and sector or job zone) growing more than 30% of the current workforce. A value of 50% would indicate that the number of additional workers required to deliver the transition in a given state and sector (for example, agriculture in California) is equivalent to 50% of that state’s current electricity workforce. TCPU stands for Transportation, Communications, and Public Utilities. FIRE stands for Finance, Insurance, and Real Estate. Miscellaneous services include sectors such as education, healthcare, and the arts.

More stringent emission-reduction targets intensify the differences across sectors. In the 95% by 2050 trajectory (Extended Data Fig. 5), Nevada leads employment growth with massive solar expansion, creating utilities and electrical equipment jobs. These sectors are dominated by lower-skill jobs. A substantial growth in agriculture (albeit from a small start) is required to drive the localized expansion of bioenergy with CCS (Oregon and Louisiana) to deliver net zero. The contraction of natural gas (Montana and Kentucky) may start to reduce employment. Notably, realizing net zero by 2035 would lead to job losses in mining equivalent to up to 10% of Wyoming’s current electricity workforce (Extended Data Fig. 6), which is consistent across the assumptions for energy systems (Extended Data Fig. 1). The growth in other sectors may not be able to compensate for the bulk of the job losses, indicating the lack of local transition options and the potential need to migrate some of the displaced mining and utility workforces.

**Fig. 5:**
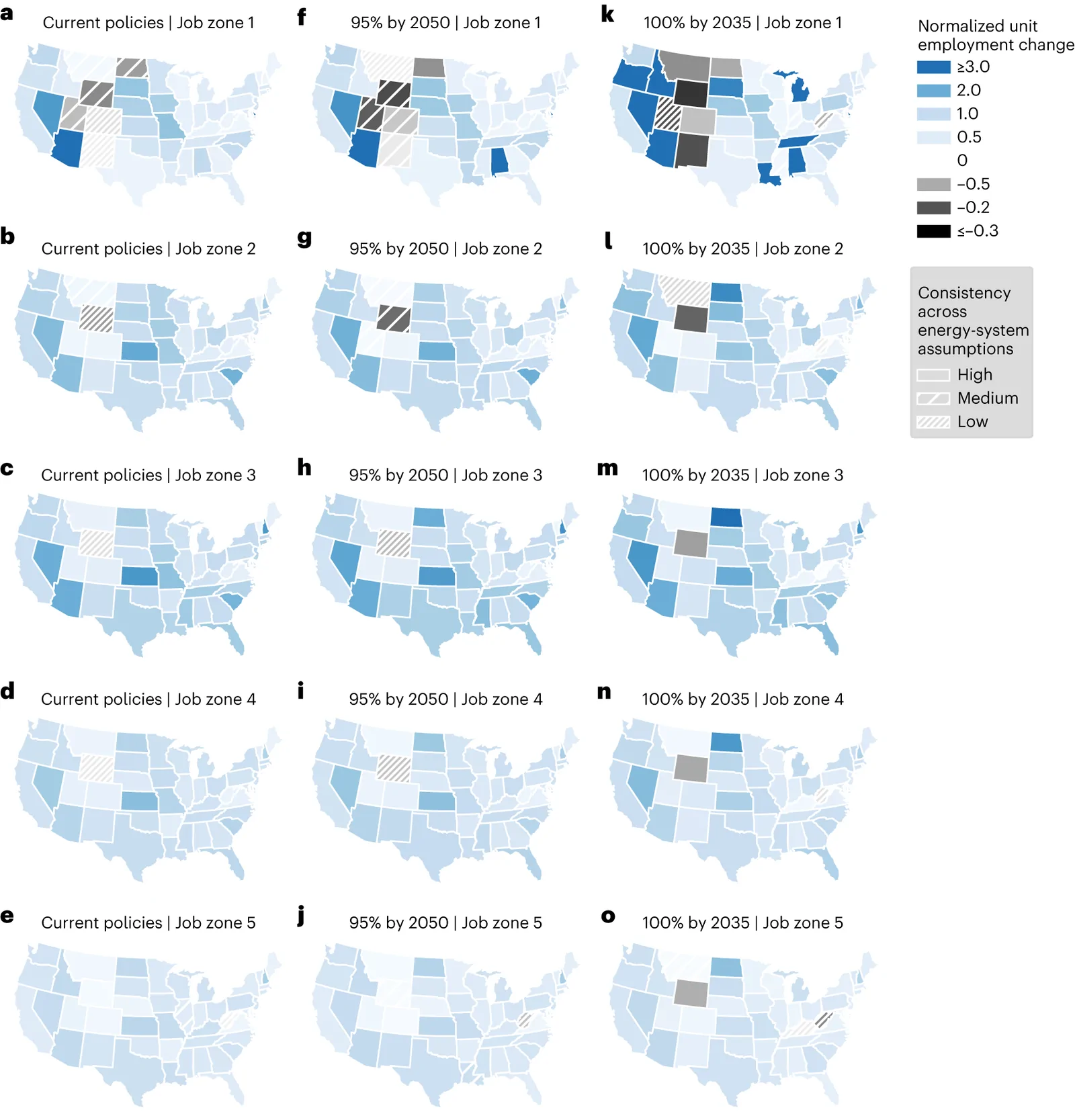
Modelled normalized unit employment change in job zones 1 through 5 for the 2040s compared to the 2020s. Normalized unit employment change (equation (10)) refers to the 2040s workforce in the state and job zone compared to the current workforce under the same category (excluding those in natural retirement), normalized to the size of the current state and job zone workforce now. Job zones range across 1 (little or no preparation, for example, conveyor belt operator), 2 (some preparation, for example, engine assembler), 3 (medium preparation, for example, geothermal production manager), 4 (considerable preparation, for example, construction manager) and 5 (extensive preparation, for example, chief sustainability officer)^48^. A value of 0.5 indicates 50% more opportunities. A value of −0.3 indicates that 30% fewer future jobs while accounting for natural retirement. **a**–**e**, Emission trajectories for current policies for job zones 1 (**a**), 2 (**b**), 3 (**c**), 4 (**d**) and 5 (**e**). **f**–**j**, The 95% reduction by 2050 constraint for job zones 1 (**f**), 2 (**g**), 3 (**h**), 4 (**i**) and 5 (**j**). **k**–**o**, The 100% reduction by 2035 constraint for job zones 1 (**k**), 2 (**l**), 3 (**m**), 4 (**n**) and 5 (**o**). Blue colours represent employment gains, and greys represent losses. Hatching denotes the level of consistency across energy-system assumptions within each group. High consistency means all sensitivities have the same sign of change as the mid-case (always positive or always negative). Medium and low consistencies represent more than and less than 80% of the 14 energy-system assumptions sharing the same sign as the mid-case, respectively. The base maps are from the US Census Bureau^85^.

The skill-level analysis is based on the job zones developed by the Occupation Information Network. Job zones are categorized by the education, training and experience required^48^. Jobs requiring some preparation (job zone 2) are highly demanded in most states (Fig. 4), whereas the lowest-skilled jobs (job zone 1) have geographically varied outcomes, especially with more stringent emission-reduction targets (Fig. 5). Job zone 1 workers in Wyoming and other eastern Mountain states are most negatively affected due to the large numbers employed in the mining sector. Western Mountain states could see low-skilled job growth through infrastructure expansion. Historically, the workforce primarily contained jobs at middle-skill levels (job zones 2 and 3) (Supplementary Section 6.5). Job needs in these areas are more evenly distributed and primarily positive across scenarios. Higher-skilled jobs would experience the slowest growth compared to others. North Dakota’s renewable energy development (especially solar) will contribute to the notable expansion of its high-skill workforce. Although high-skilled jobs in West Virginia and Kentucky may experience losses in the mid-case, these become gains under a future of low nuclear and CCS costs.

### Social dynamics in the communities

Job growth will affect different groups and communities based on their historical and likely future demographic compositions. The construction and manufacturing jobs created through the low-carbon transition tend to be male-dominated^15^. The changing employment landscape could also alter local communities. For example, the North Dakota oil boom was accompanied by social service challenges and shifting cultures^49^. The potentially substantial influx of low-skilled infrastructure workers in western Mountain states may perpetuate the structural inequalities in these states due to their lower economic development^50^. A Just Energy transition means equitably distributing the benefits and ills^51^ while accounting for the intersectionality of these social outcomes^52^.

**Fig. 6:**
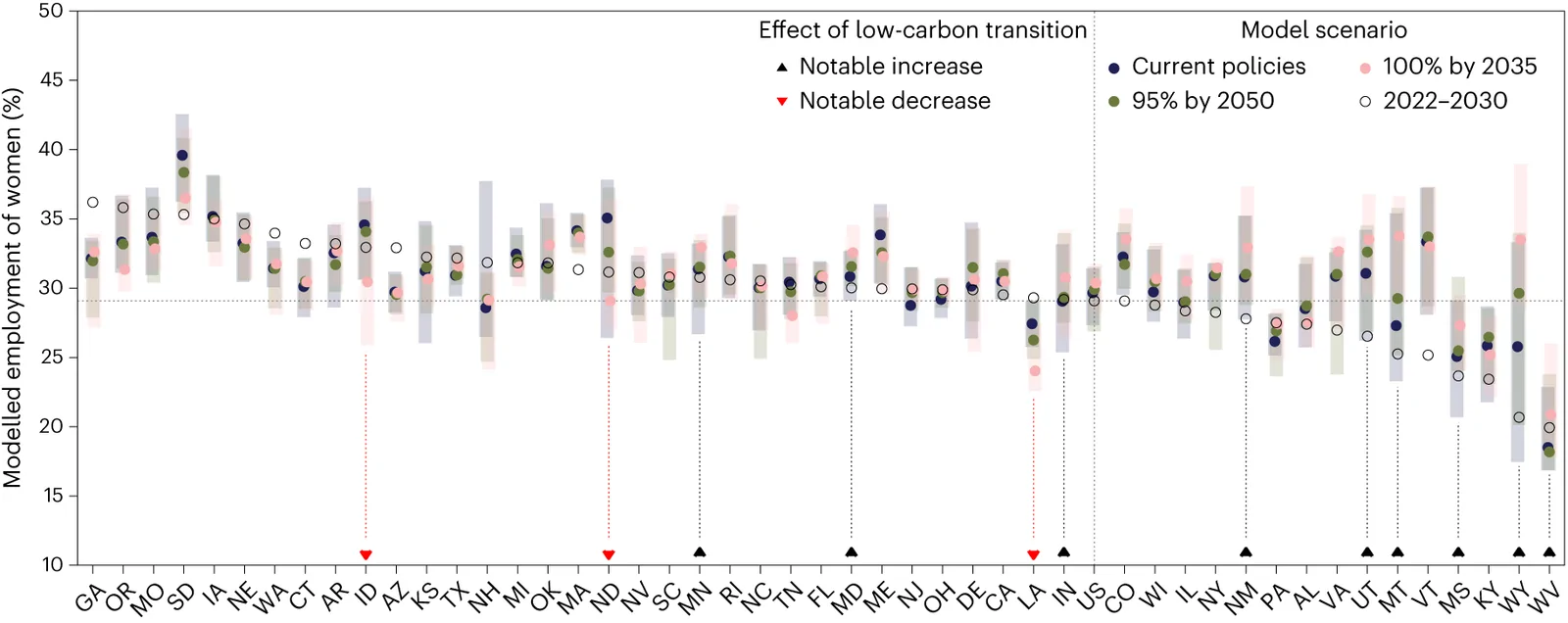
Modelled percentage of women in the power-sector workforce. The bars represent the range of outcomes in the 2040s from the full suite of energy-system assumptions. The filled circles represent the values from the mid-case scenarios. The empty circles represent the starting value (the 2020s) of current policies, by which the states are sorted in descending order. States are labelled with black up or red down arrows if the most ambitious outcomes (mid-case 100% by 2035) show *P* < 0.01 compared to current policies and their mean value lies 5% above or below the mean of current policies variants, respectively.

Low gender and racial diversity persist across the US energy sector^53^. Globally, renewable energy employs a larger share of women (32%) than fossil fuels (22%), but mostly in administrative roles^37^. The three sectors that tend to employ the lowest proportion of women are mining, construction and agriculture^54^ (Supplementary Fig. 22). We analysed the modelled composition of women in each state’s power-sector workforce in the 2040s (equation (5)), assuming that no policy interventions would disrupt the current demographics (Fig. 6). The current policies and 95% by 2050 trajectories do not change the gender composition substantially, as the structural changes required are not as disruptive. Realizing net zero by 2035, conversely, may disproportionally bring less benefits to women in some states. These states may be impacted by growth in (currently) male-dominated construction work, especially for solar (North Dakota) and bioenergy with CCS (Idaho and Louisiana). For states starting with lower percentages of women than the average employed in the power sector, net zero may present considerable opportunities to improve workplace gender diversity. Minnesota, Maryland, Indiana, Montana and Mississippi may benefit from the shift away from natural gas. New Mexico, Utah, Wyoming and West Virginia may benefit from the transition from coal. The transition from fossil fuels to renewable energy will shift jobs from mining to utilities, which have employed more women historically. The decline of male-dominated jobs does not necessarily mean unemployment, as these workers could shift to other sectors^55^. The improvements, however, do not raise women’s employment opportunities in the power sector much above the national status quo. The lower employment opportunities for women identified for the US low-carbon transition resemble those found by similar analyses of China^56^ and the EU^57^, highlighting the need for policy interventions.

In addition to the added climate vulnerability that people of colour bear^58^, the clean-energy workforce notably lacks representation of Black workers^59^. Moreover the coal-fuel sector employs fewer people of colour than average^47^. The racial dynamics in the workforce^60^, changes to racial demographics (Supplementary Fig. 28) and the impacts of potential equity-based high-road labour policies^61^ are complex and not covered by this study. Therefore, the scope for detailed projections of racial employment is limited. Instead, we highlight the uneven distribution of opportunities if existing trends persist. In 2021, Asian Americans and Black or African Americans constituted 2.1% and 6.3% of the construction workforce, although they comprised 5.9% and 13.4% of the national population (Supplementary Fig. 27). Hispanic Americans (18.5% of the national population) could benefit from the growth in construction. They were 32.6% of the sector in 2021, growing remarkably from 20.3% in 2003 (Supplementary Fig. 28). This over-representation could introduce vulnerabilities, as the quality and stability of these jobs may not be guaranteed^62,63^. The rapidly growing electrical equipment sector includes 12.5% Hispanic Americans and 6.4% Black Americans (9.7% and 6.0% in 2011), leaving these under-represented communities behind (Supplementary Fig. 28). From the pipeline, Black students, especially Black women, are notably under-represented in science, technology, engineering and mathematics^64^. Unless intentional policy planning recognizes the challenges faced by these under-represented populations and engages them in the process, the existing social inequalities may widen. The lack of workforce demographic data for Indigenous people demonstrates their invisibility regarding the employment benefits possible through centralized low-carbon transitions. The Red Lake Nation’s Tribal Energy Development Organization^65^ sets an example for the much-needed Indigenous ownership of energy initiatives.

## Discussion

The labour demands for decarbonizing the US power systems will be substantial, but no more so than historical technology and energy-based workforce mobilizations. The lowest-skilled workforce may experience more varied gains and losses than those with higher-skilled jobs. However, even with some preparation, many could benefit from job growth. The impacts of automation and other productivity increases are unknown and likely to vary between sectors and skill levels, which may counter this job creation potential. Early-mover states may also gain comparative advantages in skills expertise in the medium term^66^.

### Policy implications

Although most states will benefit from the low-carbon transition, some with energy supply chains unaligned with the low-carbon transition may lose out through reduced employment growth. Targeted compensatory policies^7^ and training programmes would be essential to support the more vulnerable section of the workforce^55^. Learning from the decline of coal in the UK, Germany and Canada, intentional engagement with the affected industries, developing attractive future scenarios with them in mind and deploying targeted policies to aid sectoral restructuring will be key to preventing social conflict^55,67,68^. Although US coal demand has already declined notably^68^, reduced natural gas reliance in a decarbonized power system would imply similar regional challenges requiring policy interventions. Broader and more proactive support packages for affected communities^7,18^ and engaging local stakeholders in the transition could improve distributional and procedural justice^19^. Unaligned states could also leverage support from the Inflation Reduction Act^8^ to expand manufacturing for clean-technology supply chains. Additional investment should support existing programmes aimed at reskilling ex-coal workers and provide federal-level Trade Adjustment Assistance for jobs displaced through the low-carbon transition^69^.

The transition itself will not lead to a disruptive change away from the social status quo unless policymakers, private-sector actors and the education system intervene. For example, leveraging the gender diversity of thought^70^ in political leadership could boost the movement because per capita emissions are lower in countries where women have a higher political status^71–73^. For state workforces at risk of becoming more homogeneous, policies encouraging gender and racial diversity across the board may be crucial^74^. The US government’s commitment to dedicate 40% of federal investments to disadvantaged communities^75^ is a good start. Labour policies targeted at social equity could present additional opportunities in the manufacturing sector due to wage improvements^61^. In the private sector, improving the perception of gender roles and flexibility in the workplace are fundamental for women’s career advancement in the energy sector^37^. Educational practices could improve the experience of Black students, including introducing culturally relevant pedagogy in the curriculum and accommodating students’ psychological needs^76^.

### Paths forward

In a changing employment landscape, continuing this work will provide decision-makers with insights for proactively managing future challenges and embracing new opportunities. The published results may provide additional near-term insights into different temporal, regional and technological aspects. Our methods and open code could enable others to study net-zero labour transitions in other countries, especially once new data are available in currently data-poor regions. Similar analyses could explore the distributional employment impacts of other energy sectors. Several limitations in our work could be addressed in future research. The JEDI input–output method should be more regularly validated with ex post empirical employment analyses^77^. This work evaluates direct and indirect jobs from the power-sector transitions. The employment change comparison here cannot account for labour (im)mobility and might under-represent sectoral unemployment effects^78^. Future work could adopt dynamic models that capture the induced employment impact and account for (im)mobility through state-level economic activities and cross-state trades.

Energy-system models (including ReEDS) can by no means predict energy futures^79^, but the suite of standard scenarios provides robust insights under a range of energy-system assumptions. Further research by the energy and employment modelling communities may bring additional robustness. Introducing workforce mobilization constraints, sociopolitical factors and financing limitations into the energy models could produce interesting alternatives to the least-cost pathways^79–83^. More detailed task- and skill-based occupation analyses could highlight the energy jobs more susceptible to automation^84^ and the viable pathways for reskilling. More in-depth sensitivity analyses of both ex ante model results and ex post studies could uncover the underlying energy-system drivers for employment outcomes and better inform techno-economic assessments.

## Methods

The analytical framework in this study couples three sets of models and data sets (Fig. 1) to convert US state-level energy-system transition pathways to distributional employment impacts. The analysis begins by using a power-sector model to provide new-build capacity (megawatts) and the annual amount of electricity generated (MWh) across different power technologies *i*. These values are used as inputs to an employment impact model, in which construction, installation and manufacturing (CIM) jobs are attributed to the capacity, and operations and maintenance (O&M) jobs are attributed to the power generated across different industry sectors *j*. These jobs are then summarized and mapped onto existing demographic databases giving the labour composition across gender and skill level. This step estimates how these distributional factors change as a function of the future configuration of power systems. Analyses of new opportunities and employment displacements account for the natural retirement of the workforce and the mismatch between the current and the future power-sector workforces in specific industries and skill levels.

### Power-system transition scenarios

The ReEDS model is an open-source power-sector capacity-planning model developed and maintained by the National Renewable Energy Laboratory (NREL)^39^. The power-sector transition scenarios analysed here are the 2021 standard scenarios^41^. The outputs include installed capacity (*C*_*i*,*t*_), annual electricity generation (*G*_*i*,*t*_) and CO_2_ emissions at the state level for the 48 continental states. Here *i* refers to the technology and *t* refers to the year. They are reported in 2-year steps from 2022 to 2050. The data points reported for years in-between are linearly interpolated. These standard scenarios (Fig. 1 and Supplementary Section 1) include 14 assumptions for the future energy system based on a wide range of input sensitivities, including future fuel prices, technology costs and the scale of electrification, to represent various energy futures^39^. The energy-system sensitivities used in this analysis include: (1) reduced renewable energy resources, (2) an extension of the Production Tax Credit and Investment Tax Credit, (3) mid-case, (4) low renewable energy costs, (5) low nuclear and CCS costs, (6) low natural gas prices, (7) low demand growth, (8) high transmission capacity, (9) high renewable energy costs, (10) high natural gas prices, (11) high growth of demand, and (12) hybrid PV and batteries. The sensitivity case with the deployment of DAC was analysed separately. The emission-reduction trajectories include current policies, 95% emission reduction by 2050 compared to 2005 levels (95% by 2050) and 100% reduction by 2035 (100% by 2035), with the first two cases also featuring the absence of nascent technologies^41^.

The ReEDS Standard Scenario Viewer does not explicitly state the amount of new-build capacity in each time step, so this is derived from the change in total installed capacity and our calculation for the capacity that would retire. The capacity of new-build facilities (${\Delta C}_{i,t}$) is used to calculate the associated CIM jobs in the next step. Data on the existing fleet of generators and their retirement schedules were taken from the Generator Database of the National Energy Modeling System developed by the US Energy Information Administration. These data were input into the ReEDS model. Assuming no early retirement, we assigned that generators would either retire in their scheduled retirement year or at the end of their lifetime (Supplementary Table 1), whichever came first. For states with existing fleets of certain technologies, three options of nameplate capacities were used in the next steps of the analysis to incorporate the employment uncertainty for new builds (Supplementary Figs. 5–8). Although the Scenario Viewer does not provide data on the expansion of transmission capacity, these data are available upon request from the ReEDS team.

### Spatial and sectoral employment analysis

The JEDI model is an open-source model developed and maintained by NREL to evaluate the employment and economic impact of constructing and operating power generation technologies^40^ and the associated interregional transmission network. Although decommissioning retired plants also generates job opportunities, this is not considered in this work. The model operates on an input–output basis using the IMPLAN database^87^. The model was selected due to its spatial granularity at the state level and data accessibility. The JEDI framework uses a detailed capital and operational cost breakdown of each technology to calculate the impact of local and state-level job creation in 14 main economic sectors: (1) agriculture, (2) mining, (3) construction, (4) manufacturing, (5) fabricated metals, (6) machinery, (7) electrical equipment, (8) transportation, communications and public utilities, (9) wholesale trade, (10) retail trade, (11) finance, insurance and real estate, (12) professional services, (13) government and (14) miscellaneous services. Direct jobs associated with power generation and indirect jobs associated with the supply chain were used. Although the JEDI models provide information on induced jobs, they are not included in the analysis due to the uncertainties in addressing their microeconomic interactions, especially interstate trading.

The existing suite of models includes technologies such as biopower, coal, concentrated solar power, geothermal, conventional hydro, land-based wind, offshore wind, natural gas combined cycle, PV (both rooftop and utility-scale) and transmission. Additional technologies not provided by the JEDI website include batteries (2 to 10 h), biopower with CCS, DAC, hydrogen combustion turbine, natural gas combined cycle with CCS, nuclear, oil/gas steam-turbine generators and PV with batteries. We created proxy JEDI models for these technologies by modifying the cost components in existing models of similar technologies. Further details on the assumptions made in these modifications can be found in Supplementary Section 3.

The local shares of technology components are primarily based on the JEDI default values. For fuel-consuming technologies, the local shares of the fuel are based on state-level historical fuel production and consumption data from the Energy Information Administration^88,89^, which can be found in Supplementary Section 5.1. Local shares of uranium are assumed to be zero since historically purchased imports have supplied the majority of the fuel^90^. The local shares of biomass feedstocks are assumed to be 100% because the states themselves can generally produce more than is required for power generation. Details of future interstate biomass trades are too uncertain to quantify in the scope of this work (Supplementary Section 5.2).

Although O&M jobs for fuel-consuming technologies depend on the amount of fuels consumed, those for renewable technologies are more fixed. In the JEDI models, O&M jobs (${N}_{i,j,t}$) are assumed to be constant long term and are calculated annually for the operating conditions (that is, the plant capacity factor) in units of jobs. The CIM jobs ($M_{i,j,t}$) are calculated in units of job-years (or full-time equivalents) during the construction period. In simple terms, these jobs are calculated by the JEDI models as a function of generation and capacity:

1 $${N}_{i,j,t}=f\left({G}_{i,t},{C}_{i,t}\right)\quad(\,{\rm{jobs}})$$

2 $${M}_{i,j,t}=f({\Delta C}_{i,t})\quad(\,\mathrm{job-years})$$

The summation of O&M jobs and the shorter-term CIM jobs assumes that the latter (in job-years) span equally over the construction period (T_const_) in years (Supplementary Table 1). Given the long timespan of the analysis, the change in labour productivity is simply estimated as an annual 1% increase compounded over the transition period^12^. An analysis of the robustness of this assumption can be found in Supplementary Section 6.1. The final job impacts were calculated annually for the 14 economic sectors and 48 states:

3 $${X}_{i,j,t}={N}_{i,j,t}+\frac{({M}_{i,j,t}+{M}_{i,j,t+1}+\cdots +{M}_{i,j,t+\mathrm{{T}_{const}}-1})}{{T}_{const}}\quad({\,\rm{jobs}})$$

### Distributional impact analysis of skill levels

The Occupation Information Network introduced the job zone^48^ framework to describe the education, related experience and on-the-job training required for different occupations. The job zones range from ‘little to no preparation needed’ (job zone 1) to ‘extensive preparations needed’ (job zone 5). The job zone designations for occupations are updated by expert analysts from 2004 (for example, telemarketers) to 2021 (for example, chief sustainability officers). We utilized the full set of occupations in the database, assuming that they would still be in the same job zones as when they were designated. This framework was used as a proxy for skill levels and training requirements. The assignment of occupations to industries^91^ and job zones^92^ and their compositions in states^93^ were used to calculate the job zone compositions of the workforce across industries and states (Supplementary Section 6.5).

Assuming that the current compositions of skills apply to the future workforce, the power-sector employment associated with industry sector *j* and job zone *z* in state *s* in year *t* can be calculated as

4 $${X}_{s,j,z,t}=\sum _{i}{P}_{z,s,j}{X}_{s,j,t}\quad({\,\rm{jobs}})$$

where ${P}_{z,s,\,j}$ is the percentage of jobs in job zone *z* in the workforce of industry sector *j* in state *s* and ${X}_{s,j,t}$ is the employment associated with industry sector *j* in year *t* and state *s*.

### Distributional impact analysis of gender

The American Community Survey run by the US Census Bureau^54^ provides the gender distribution of the workforce by state and industry. Given the continuing need to expand nuancedly gendered demographic data^70^, in this work, we interpreted ‘sex’ as ‘gender’ (that is, males were interpreted as men and females as women). The historical trends in the gender distributions (Supplementary Fig. 22) across industry sectors remained relatively steady from 2010 to 2020. Thus, we assumed the gender distributions would remain at 2020 levels (Supplementary Fig. 24) for the planning horizon. The modelled composition of women in the power-sector workforce (modelled employment of women) in year *t* in state *s*, *W*_*s*,*t*_ was calculated as follows:

5 $${W}_{s,t}=\frac{\sum _{j}{W}_{s,j}{X}_{s,j,t}}{\sum _{j}{X}_{s,j,t}}\quad( \% )$$

where *W*_*s*,*j*_ is the percentage of women employed in industry sector *j* in state *s* and ${X}_{s,j,t}$ is the total number of jobs associated with the power-sector workforce disaggregated to state *s* and industry sector *j* in year *t*. This modelled composition does not project the absolute future of women’s employment in the power sector. Instead, it can be used to compare the potential impacts in different transition scenarios and highlight the need for gender-related policy support in certain states, especially if the gender dynamics in the workplace remain static.

### Employment opportunities and challenges

The employment transition indices were calculated on the basis that a portion of the current workforce would naturally retire throughout the nearly 30-year planning horizon. Assuming that the current workforce will retire at age 65, the snapshot of the retired workforce can be calculated from the age distribution of the current workforce across industries^94^, as shown in Supplementary Fig. 25. Due to the lack of state-level data and the small variations (roughly 5%) in age group compositions aggregated over states (Supplementary Fig. 26), we assumed that the same sectoral age distributions apply to all states. The remaining historical workforce can be calculated as

6 $${\Delta X}_{s,j,{t}_{n}}={X}_{s,j,{t}_{0}}(1-{P}_{\mathrm{retire},\,j})\quad( \% )$$

where ${X}_{s,j,{t}_{0}}$ is the workforce attributed to state *s* and industry *j* at the start of the planning horizon (2022) and ${P}_{\mathrm{retire},\,j}$ is the percentage in industry *j* estimated to retire by the end of the period.

The numbers of future jobs by state, sector and job zones are compared against the remaining historical workforce. The employment change (EC_*s*_,_*d*_) is the difference between the number of jobs at the end of the planning horizon and the workforce remaining from the start of the planning horizon. It shows the new opportunities created through the transition. It can be calculated as

7 $$\operatorname{EC}_{s,d}={X}_{s,d,{t}_{n}}-{\Delta X}_{s,{d,t}_{n}}\quad(\text{thousand jobs})$$

where ${X}_{s,d,{t}_{n}}$ is the workforce attributed to state *s* and distributional area *d* (industry *j* or job zone *z*) at the end of the planning horizon. Here we assume that the value at the end of the planning horizon is the average value in the final decade (2041–2050) to minimize the effect of drastic changes in certain years. This indicator is reported in the unit of thousand jobs. When it is positive, it indicates the absolute scale of expansion of the current workforce. For example, a value of 1 means an additional 1,000 new entrants would be needed to deliver the transition. When this value is negative, it indicates the scale of unemployment risks for the current workforce. For example, a value of −1 implies that 1,000 current workers would lose their jobs (in addition to the natural retirement), so that there would be a need to prepare for retraining or migration in the next three decades.

When comparing the employment change across emission-reduction scenarios A and B, the net employment change (NetEC) is defined as

8 $$\begin{array}{l}{\mathrm{NetEC}}_{s,\mathrm{AB}}=\left({X}_{s,{t}_{n},\mathrm{A}}-{\Delta X}_{s,{t}_{n}}\right)-\left({X}_{s,{t}_{n},\mathrm{B}}-{\Delta X}_{s,{t}_{n}}\right)\\ \qquad\qquad\quad={X}_{s,{t}_{n},\mathrm{A}}-{X}_{s,{t}_{n},\mathrm{B}}\quad(\text{thousand jobs})\end{array}$$

where ${X}_{s,{t}_{n},{\mathrm{A}}}$ and ${X}_{s,{t}_{n},{\mathrm{B}}}$ are the total number of jobs in each state in the final decade (2041–2050) for scenarios A and B. ${X}_{s,{t}_{0}}$ is the total number of jobs in each state at the start of the transition. This indicator is reported in thousands of jobs. When it is positive, it indicates that scenario A creates more jobs than scenario B. For example, a value of 1 means that scenario A has created 1,000 more jobs by the final decade than scenario B.

When comparing the sectoral and job zone distributed impacts, we normalized the values by the size of the state electricity workforce to avoid overshadowing the results from smaller states. This indicator is the normalized state employment change (NormSEC):

9 $${\mathrm{NormSEC}}_{s,d}=\frac{{\mathrm{EC}}_{s,d}}{\sum {X}_{s,{t}_{0}}}\quad( \% )$$

where ${\mathrm{EC}}_{s,d}$ is the employment change attributed to state *s* and distributional area *d* (industry *j* or job zone *z*) and $\sum {X}_{s,{t}_{0}}$ is the size of the state electricity workforce at the start of the planning horizon. A value of 50% would indicate that the number of additional workers required to deliver the transition in a given state and sector (for example, agriculture in California) is equivalent to 50% of that state’s current electricity workforce.

Alternatively, when analysing the impact of a specific job zone (or sector) in a specific state, we normalized the value by the size of the job zone workforce in that state. This normalized unit employment change (NormUEC) represents the impact for the designated section of the workforce:

10 $${\mathrm{NormUEC}}_{s,d}=\frac{{\mathrm{EC}}_{s,d}}{\sum {X}_{s,d,{t}_{0}}}\quad( \% )$$

where ${\mathrm{EC}}_{s,d}$ is the employment change attributed to state *s* and distributional area *d* (industry *j* or job zone *z*) and $\sum {X}_{s,{d,t}_{0}}$ is the size of the distributional area and state electricity workforce at the start of the planning horizon. A value of 50% would indicate that an additional subset of the workforce (for example, agriculture in California) would be 50% of the size of the current subset of the workforce (for example, agriculture in California) would be required to deliver the transition.

## Online content

Any methods, additional references, Nature Portfolio reporting summaries, source data, extended data, supplementary information, acknowledgements, peer review information; details of author contributions and competing interests; and statements of data and code availability are available at https://doi.org/10.1038/s41558-023-01802-5.

## Supplementary information

## Data Availability

The information contained herein was obtained using ReEDS, which was developed by and licensed from the Alliance for Sustainable Energy, LLC, which is the manager and operating contractor of the NREL on behalf of the US Department of Energy. The ReEDS model is open access on GitHub upon request through the NREL website (https://www.nrel.gov/analysis/reeds/request-access.html). The JEDI models are open-access Excel sheets available from the NREL website (https://www.nrel.gov/analysis/jedi/models.html). The 2021 ReEDS standard scenarios can be accessed from the Scenario Viewer (https://scenarioviewer.nrel.gov/) along with standard scenarios from previous years. The databases produced by the US Energy Information Administration, Census Bureau and Bureau of Labor Statistics can be found in their relevant sections as they are mentioned throughout the manuscript. The results data sets for the employment calculations can be found on Zenodo (https://doi.org/10.5281/zenodo.7800258).

## Extended data

Extended data are available for this paper at https://doi.org/10.1038/s41558-023-01802-5.

## Code availability

The code used in this work can be accessed from its GitHub repository under the Apache 2.0 Licence (https://github.com/judyjwxie/LoCaTED). The framework is named the Low Carbon Transition Employment Distribution (LoCaTED) model^95^ and is set up to run by inputting additional open-access files from ReEDS and JEDI. Refer to the README file for more instructions.
