# Supplementary material for: Distributional labour challenges and opportunities for decarbonizing the US power system
Source: Nat Clim Chang. 2023 Nov 2;13(11):1203–12. doi: 10.1038/s41558-023-01802-5 (PMC13538061; doi:10.1038/s41558-023-01802-5)
Supplement: Supplementary file 1 [file 41558_2023_1802_MOESM1_ESM.pdf]

nature climate change

Article

https://doi.org/10.1038/s41558-023-01802-5

Distributional labour challenges and opportunities for decarbonizing the US power system

In the format provided by the authors and unedited

Contents

List of Figures 2

List of Tables 4

1 ReEDS Standard Scenarios 6

2 Original JEDI Models 11

3 Modified JEDI Models 17
- 3.1 Nuclear . . . 17
- 3.2 Nuclear SMR . . . 20
- 3.3 Oil-gas-steam . . . 21
- 3.4 NG-CC-CCS . . . 22
- 3.5 Coal-CCS . . . 23
- 3.6 Bioenergy-CCS . . . 25
- 3.7 Batteries and PV-battery . . . 27
- 3.8 Renewable Energy Combustion . . . 29
- 3.9 Direct Air Capture . . . 29

4 Industrial Crosswalks 33

5 Local Share of Fuels 37
- 5.1 Coal and natural gas . . . 37
- 5.2 Biomass feedstock . . . 39

6 Demographic Databases 41
- 6.1 Productivity . . . 41
- 6.2 Gender composition . . . 44
- 6.3 Age distribution . . . 45
- 6.4 Racial composition . . . 46
- 6.5 Job Zones . . . 47

7 Modeled Employment Impacts 50
- 7.1 National Impacts . . . 50
  - 7.1.1 Aggregate Results . . . 50
  - 7.1.2 Technology-level Results . . . 51
- 7.2 State-level Impacts . . . 51
- 7.3 Sector- and Skill-based Impacts . . . 56

Supplementary References 61

List of Figures

S1 Summary of the model uncertainties in each analysis step. 6
S2 The capacities of different power technologies in the US across the 2022 ReEDS Standard Scenario outputs. 8
S3 The electricity generation of different power technologies in the US across the 2022 ReEDS Standard Scenario outputs. 9
S4 The electricity sector emission scenarios in the US in 2022 and 2050 across the 2022 ReEDS Standard Scenario outputs. 10
S5 The nameplate capacities of the existing fleet of battery, biopower, coal, concentrated solar, geothermal, and hydro in each state with an existing fleet. . 13
S6 The nameplate capacities of the existing fleet of land-based wind, natural gas combined cycle, natural gas combustion turbine, oil gas steam turbine, and photovoltaics in each state. 14
S7 The CIM and O&M employment output from the JEDI models for biopower, hydro, land-based wind, and natural gas combined cycle. 15
S8 The CIM and O&M employment output from the JEDI models for coal, geothermal, photovoltaics (utility), and concentrated solar. 16
S9 Modelled electricity system employment during 2022-2050 in the United States under the 100% reduction by 2035 with Low Nuclear and CCS Costs sensitivity. 18
S10 Modelled sectoral employment during 2022-2050 in the United States under the 100% reduction by 2035 with Low Nuclear and CCS Costs sensitivity. . . 19
S11 Modelled electricity system employment during 2022-2050 in the US under the 100% reduction by 2035 with DAC sensitivity. 31
S12 Modelled electricity system employment during 2022-2050 in Texas under the 100% reduction by 2035 with DAC sensitivity. 32
S13 The historical consumption (left) and production (right) of coal across several regions from 2001 to 2020. 37
S14 The historical consumption (left) and production (right) of natural gas across the top consuming and producing states from 2001 to 2020. 37
S15 The historical local shares of coal (left) and natural gas (right) in the top 6 consuming states in 2020. 38
S16 The flow of coal across US states. 38
S17 The flow of natural gas across US states. 38
S18 Biopower generation (left) and the percentage of generation over the state's total biopower feedstock production (right) using the ReEDS Mid Case Standard Scenario. 39
S19 Biopower feedstock production by feedstock type and state. 40
S20 US national labor productivity trends between 2007-2021 with regression between 2012-2018 (left) and 2012-2021 (right). 41
S21 US state-level labor productivity trends between 2007-2021 with regression between 2012-2021. 42

S22 Trends of US national female employment percentage from 2010 to 2020 across 14 JEDI industry sectors. 44
S23 Female employment percentage in 2010 across 14 JEDI industry sectors and 52 states. 44
S24 Female employment percentage in 2020 across 14 JEDI industry sectors and 52 states. 45
S25 Employment distribution by age group in 2021 across 14 JEDI industry sectors. 45
S26 Employment distribution by age group in 2019 across 52 states. 46
S27 National employment racial distribution in 2021 across 14 JEDI industry sectors. 46
S28 National employment racial distribution between 2003-2022 across 14 JEDI industry sectors. 47
S29 The compositions of 14 JEDI industry sectors by occupation Job Zones in US States (1 of 2). 48
S30 The compositions of 14 JEDI industry sectors by occupation Job Zones in US States (2 of 2). 49
S31 State-level Employment Change under the 100% by 2035 emission trajectory. 57
S32 Distribution of Employment Change and Normalized Unit Employment Change in the Current Policies trajectory between the 2020s and 2040s. 58
S33 Distribution of Employment Change and Normalized Unit Employment Change in the 95% by 2050 trajectory between the 2020s and 2040s. 59
S34 Distribution of Employment Change and Normalized Unit Employment Change in the 100% by 2035 trajectory between the 2020s and 2040s. 60

List of Tables

S1 Lifetime and construction time assumptions of technologies in the ReEDS model. 7
S2 Original JEDI models used for ReEDS technologies and their respective versions. 11
S3 Project descriptive data for the modified nuclear JEDI model. 17
S4 Annual operating and maintenance costs for the modified nuclear JEDI model. 18
S5 Construction costs for the modified nuclear JEDI model. 19
S6 Project descriptive data for the modified nuclear SMR JEDI model. 20
S7 Annual operating and maintenance costs for the modified nuclear SMR JEDI model. 20
S8 Construction costs for the modified nuclear SMR JEDI model. 21
S9 System descriptive data for the modified NG-CC-CCS JEDI model. 22
S10 Annual operating and maintenance costs for the modified NG-CC-CCS JEDI model. 23
S11 Retrofit construction costs for the modified NG-CC-CCS JEDI model. 23
S12 System descriptive data for the modified Coal-CCS JEDI model. 24
S13 Annual operating and maintenance costs for the modified Coal-CCS JEDI model. 24
S14 Retrofit construction costs for the modified Coal-CCS JEDI model. 24
S15 System descriptive data for the modified bioenergy-CCS JEDI model. 25
S16 Construction cost for the modified bioenergy-CCS JEDI model. 25
S17 Annual operating and maintenance cost for the modified bioenergy-CCS JEDI model. 26
S18 Project descriptive data for the modified battery (2h, 4h, 6h, 8h, and 10h) and PV-battery JEDI models. 27
S19 Intallation costs (Cost per kW) of for the modified battery (2h, 4h, 6h, 8h, and 10h) and PV-battery JEDI models. 28
S20 Annual operating and maintenance costs (Cost Per kW)in the modified battery (2h, 4h, 6h, 8h, and 10h) and PV-battery JEDI models. 29
S21 Technology and economic data for the modified hydrogen model. 29
S22 Electric capacities of DAC technologies. 30
S23 Construction costs for the modified DAC model. 30
S24 Annual operating and maintenance costs for the modified DAC model. . . . 31
S25 Industry crosswalk between the O*NET industry categories based on the North American Industry Classification System (NAICS) and the 14 JEDI industry sectors. 33
S26 Industry crosswalk between the Census industry categories and the 14 JEDI industry sectors. 34
S27 Industry crosswalk between the 22 industry sectors in the JEDI photovoltaics module and the 14 main JEDI industry sectors. 35
S28 Industry crosswalk between the Bureau of Labor Statistics (BLS) industry categories and the 14 JEDI industry sectors. 36

S29 Effect of the national productivity assumption on the national net employment in the 2040s compared to the 2020s across emission trajectories. 43
S30 Effect of the state-level productivity assumption on the national net employment in the 2040s compared to the 2020s across emission trajectories. . . . 43
S31 Comparing the effect of the state-level versus national productivity assumption on the national net employment in the 2040s compared to the 2020s across emission trajectories. 43
S32 National Employment Change in the 2040s compared to the 2020s across emission trajectories. 50
S33 National Normalized Employment Change in the 2040s compared to the 2020s across emission trajectories. 50
S34 National employment across emission trajectories. 51
S35 National employment by technology in the 2040s in three emission trajectories. 52
S36 T-test statistics on the national employment by technology in the 2040s. . . 53
S37 Modeled state-level employment under the Mid case 100% reduction by 2035 scenario contextualized by historical energy system employment and future employment projections (Part 1/2). 54
S38 Modeled state-level employment under the Mid case 100% reduction by 2035 scenario contextualized by historical energy system employment and future employment projections (Part 2/2). 55

1 ReEDS Standard Scenarios

This study uses the 2022 ReEDS Standard Scenario release from the scenario viewer [1]. Figure S1 outlines the levels of uncertainties accounted for in the analysis. In the power system, there are 70 scenarios including a suite of 5 technology sets and emission reduction trajectories and 14 energy system sensitivities. The emission reduction trajectories include Current Policies, 95% emission reduction from 2005 levels by 2050, and 100% reduction by 2035. The first two trajectories also are explored without nascent technologies, which include "enhanced geothermal systems, floating offshore wind, coal CCS, gas CCS, biopower CCS, nuclear small modular reactors, and renewable energy combustion generators" [2]. Further descriptions of the uncertainties accounted for in the employment impact and demographic databases can be found in Sections 3 and 6.

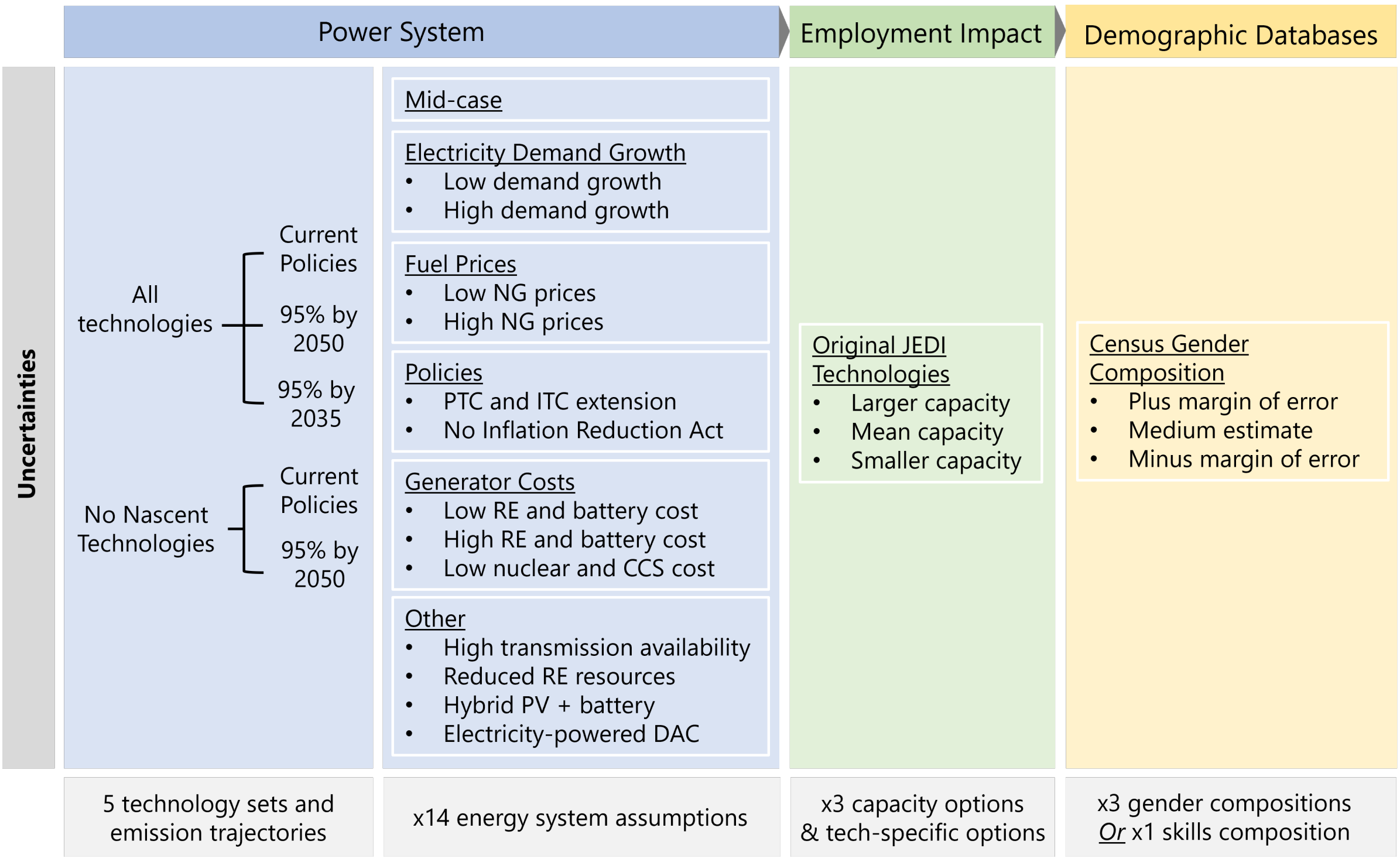

Fig. S1: Summary of the model uncertainties in each analysis step. The 70 power sector scenarios are from the ReEDS 2022 Standard Scenarios. RE is renewable energy. CCS is carbon capture and storage. PTC is the production tax credit. ITC is the investment tax credit. The employment impact uncertainties are mainly accounted for through the economy of scale. The gender composition uncertainties are accounted for through the Census measurement margin of errors. Partially adapted from [2].

Figures S2 and S3 show the snapshots of the capacities and generations, respectively, of the start and three decennial time points (2030, 2040, and 2050) from the 2022 Standard Scenarios [2]. Figure S4 represents the emission outcomes of those scenarios.

Table S1 shows the lifetime and construction time assumption in the ReEDS model Documentation [3]. Since the 15-year lifetime of batteries is shorter than the planning horizon of this work (2022-2050), those built before 2036 were rebuilt after the lifetime if their capacity was needed. The construction time for DAC is assumed to be 2 years based on ClimeWorks data [4].

Table S1: Lifetime and construction time assumptions of technologies in the ReEDS model. The values in the parentheses show the lifetimes for units larger than 100MW. The time unit is year. Data Source: [3]

Technology	Lifetime	Construction Time
Land-based wind	30	3
Offshore wind	30	3
Solar PV	30	1
Concentrating Solar Power	30	3
Geothermal	30	4
Hydropower	100	3
Biopower	50	4
Marine hydrokinetic	20	3
Battery	15	1
Natural gas combustion turbine	50	3
Natural gas combined cycle and CCS	60	3
Coal, all technologies, including cofired	65 (75)	6
Oil-gas-steam (OGS)	50 (75)	6
Nuclear	80	6

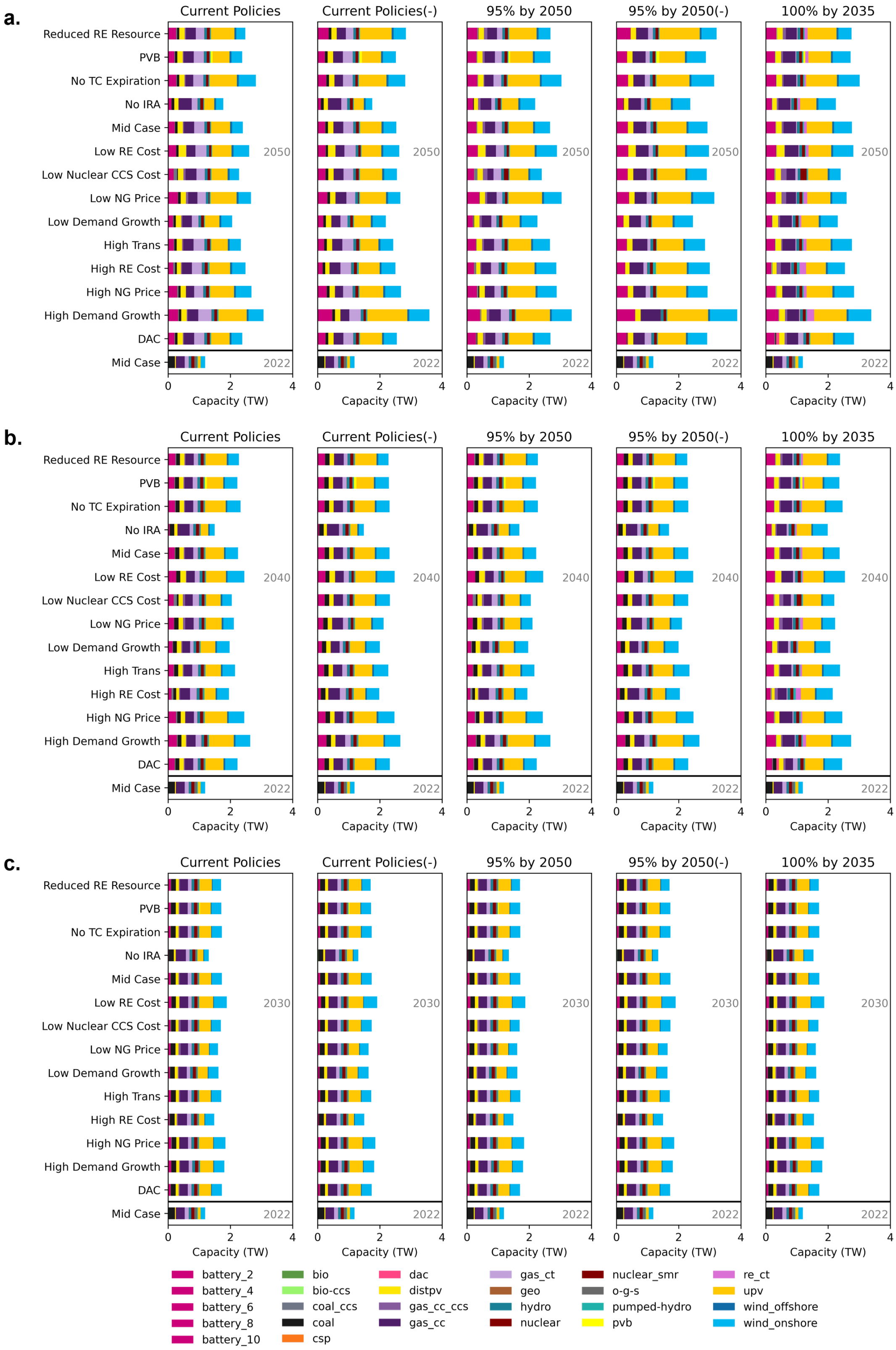

Fig. S2: The capacities of different power technologies in the US across the 2022 ReEDS Standard Scenario outputs. The decennial time points including 2050 (a), 2040 (b), and 2030 (c) are compared to 2022. The trajectories denoted (-) refer to those without nascent technologies. The Data source: [2].

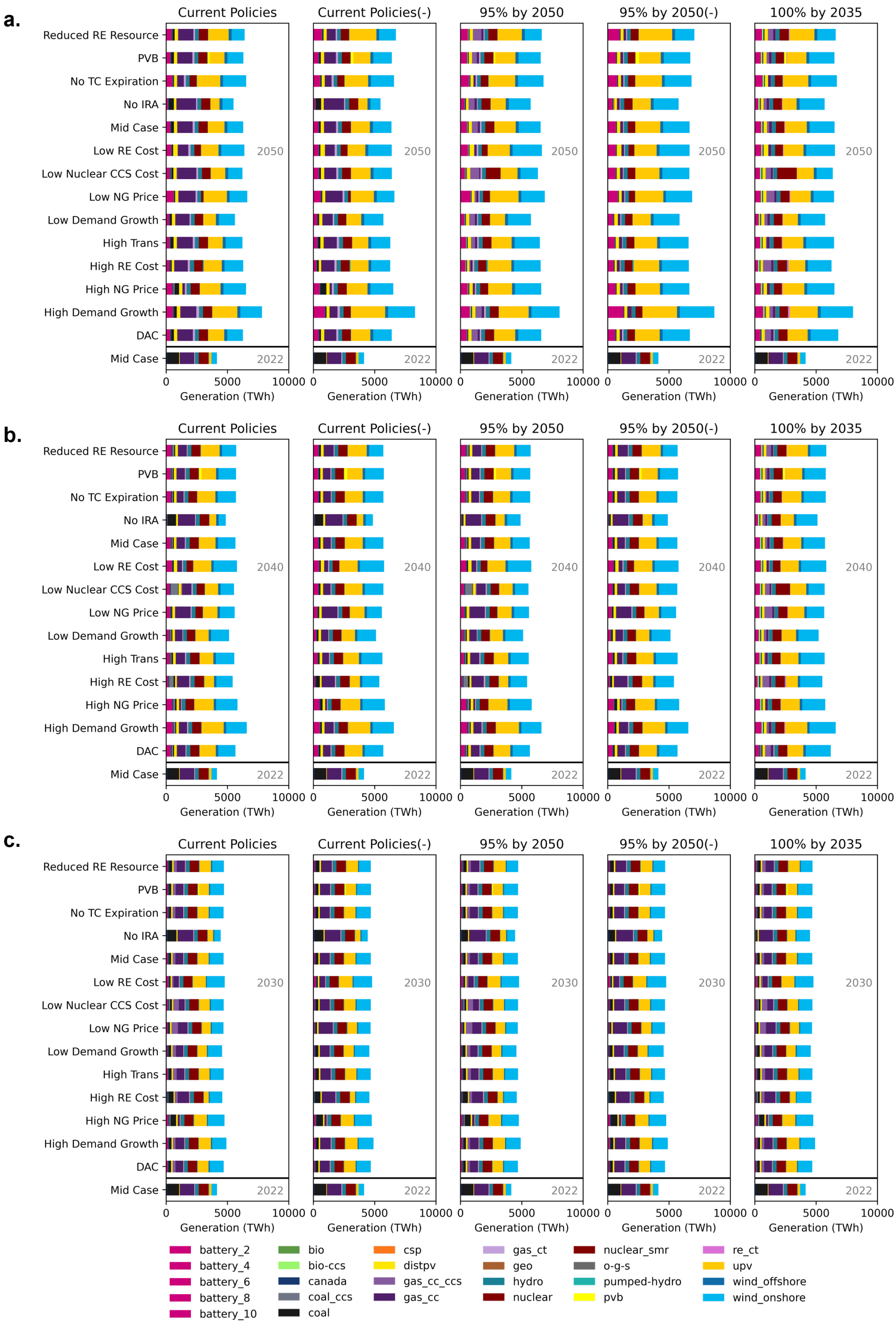

Fig. S3: The electricity generation of different power technologies in the US across the 2022 ReEDS Standard Scenario outputs. The decennial time points including 2050 (a), 2040 (b), and 2030 (c) are compared to 2022. The trajectories denoted (-) refer to those without nascent technologies. Data source: [2].

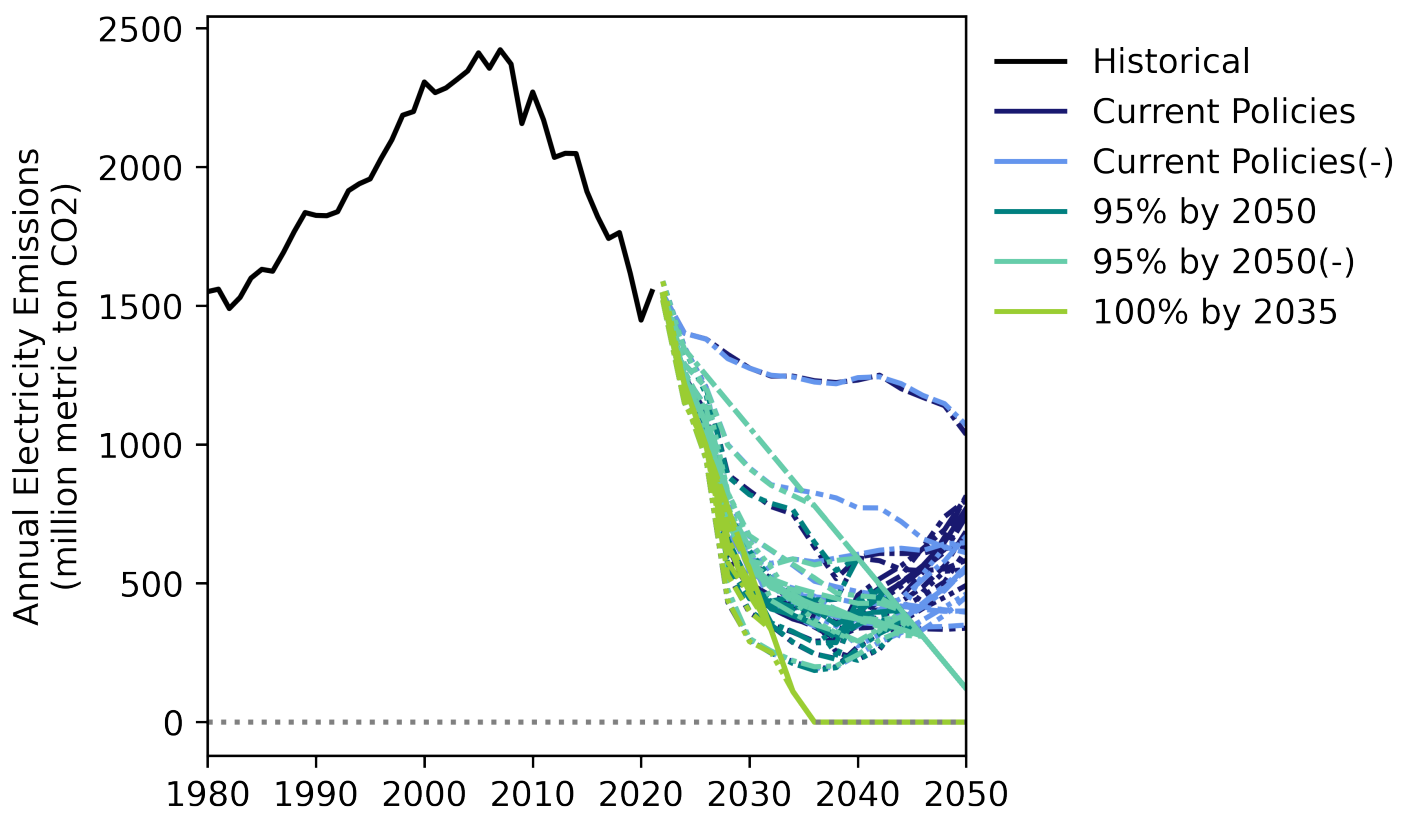

Fig. S4: The electricity sector emission scenarios in the US in 2022 and 2050 across the 2022 ReEDS Standard Scenario outputs. Data source: [1].

2 Original JEDI Models

The suite of JEDI models used in this analysis includes biopower, coal, concentrated solar power (csp), geothermal, hydropower, land-based wind, natural gas combined cycle (ng-cc), offshore wind, photovoltaics (both rooftop and utility), and transmission. The generation and capacity expansion of the power technologies were from the ReEDS Standard Scenario Viewer [1]. The transmission line data is from the assumptions in the ReEDS model Documentation [3]. The model versions used can be found in Table S2.

Table S2: Original JEDI models used for ReEDS technologies and their respective versions.

Technology	JEDI Model
biopower	01d-jedi-biopower-model-rel-b12-23-16
coal	01d-jedi-coal-model-rel-c12-23-16
csp	01d-jedi-csp-trough-model-rel-csp12-23-16
geothermal	01d-jedi-geothermal-model-rel-gt12-23-16
hydro	01d-jedi-chydro-model-rel-ch12-23-16
land-based wind	01d-jedi-land-based-wind-model-rel-w6-28-19
ng-cc	01d-jedi-ngas-model_rel-ng4-17-17
offshore wind	01d-jedi-offshore-wind-model-rel-osw10-24-17
rooftop pv	01D_JEDI_PV_Model_rel._PV05.20.21
utility pv	01D_JEDI_PV_Model_rel._PV05.20.21
transmission	01d-jedi-transmission-line-model_rel-tl12-23-16

Based on the Energy Information Administration's Form 860 [5], the nameplate capacities of the existing generator fleet can vary across different states (Figures S5 and S6). The nameplate capacity of technology could determine the job implications, especially in the construction, installation, and fixed O&M components. The effect of the economy of scale could result in lower job creation per unit of capacity with a larger generator. The output of the ReEDS model, however, does not specify the detailed capacity information of the new builds. For the simplicity of calculation, we assume a uniform generator nameplate capacity for each technology in each state. This uniform name capacity is based on the lower quartile, median, and upper quartile of the existing fleet built after 1990 (the past 30 years). For those without an existing fleet, the nameplate capacity was assumed to be the median of the existing national fleet. Note that some of the largest facilities were excluded as outliers in the statistical analysis. These include,

- a battery facility in CA (250MW)
- a geothermal facility in CA (1163MW)
- hydropower facilities in WA (6809MW – Grand Coulee Dam, 2456.2MW – Boundary Dam, and 1250MW), TN (1713.6MW - Raccoon Mountain pumped hydro), VA (2862MW - Bath County pumped hydro), NY (2429.1MW - Robert Moses Niagara dam), OR (2160MW - John Day Dam), and MI (1978.8MW - Ludington pumped hy-

dro)

- Oil gas steam in NY (1803.6MW - Oswego Harbor Power) and MA (1165MW - Canal)
- Solar PV in CA (585.9MW - Topaz Solar Farm)

Figures S7 and S8 below show the CIM and O&M job creation of each JEDI model in each state. The nameplate capacities evaluated include the three orders of magnitude from the lowest capacity currently installed, and from there, three data points linearly spaced until the largest capacity currently installed. The capacity factors for combustion-based technologies were assumed to be 100%.

Natural gas combined cycle, coal, and photovoltaics showed the smallest job creation dependency on nameplate capacity. The former two also showed the most variations in the capacities of the existing technology fleet. Biopower, hydro, land-based wind, and geothermal showed the most variations in the lower capacity range and plateaus towards larger capacities. Concentrated solar showed continuously increasing productivity in larger capacities.

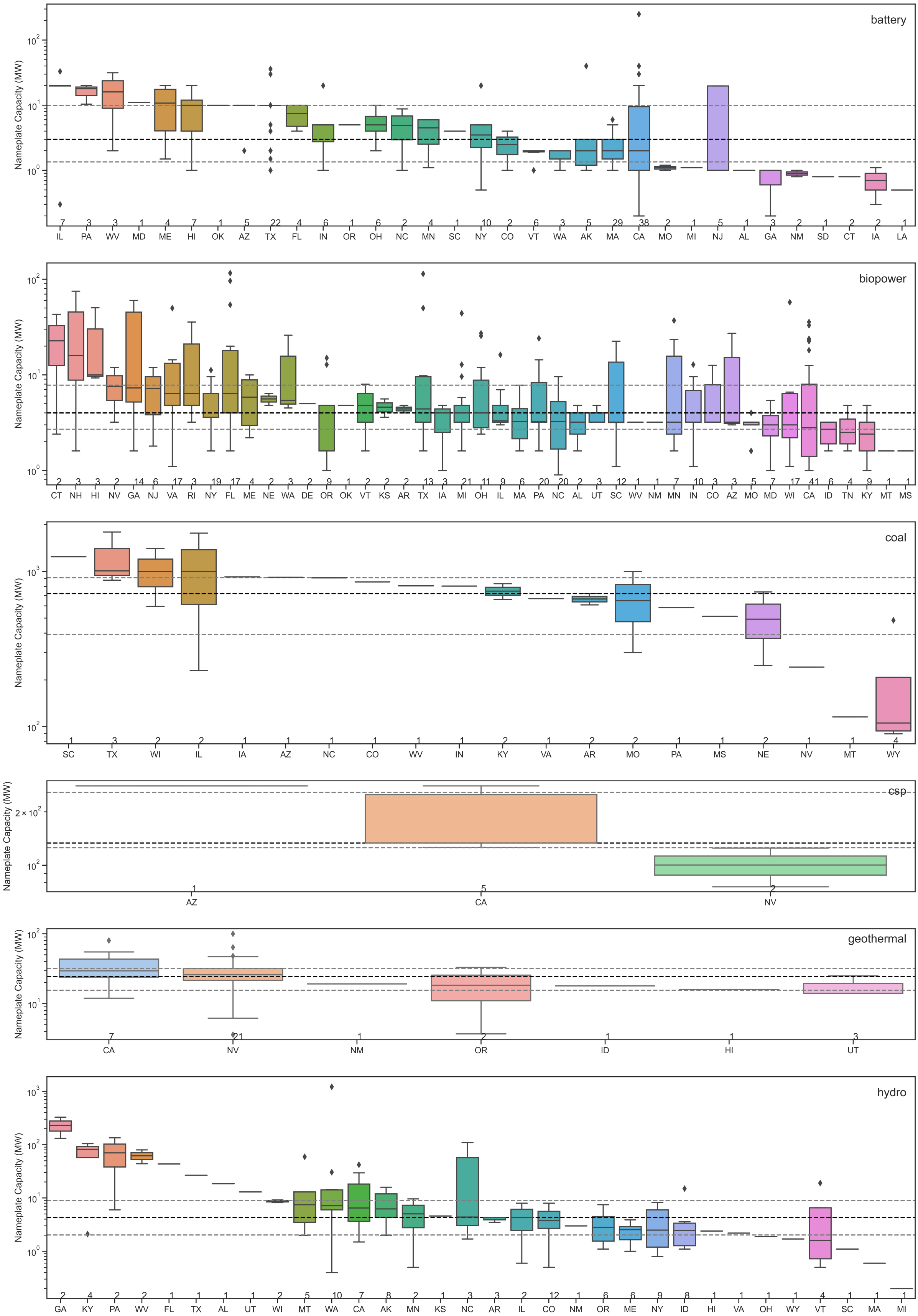

Fig. S5: The nameplate capacities of the existing fleet of battery, biopower, coal, concentrated solar, geothermal, and hydro in each state with an existing fleet. The box plots show the median, the 25-75% range (box), and data 1.5 times the interquartile range above and below the box (whiskers). The dashed lines represent the median and the 25-75% range at the national level. The sample size (n) of each technology and state is listed above the state names. Data source: [5].

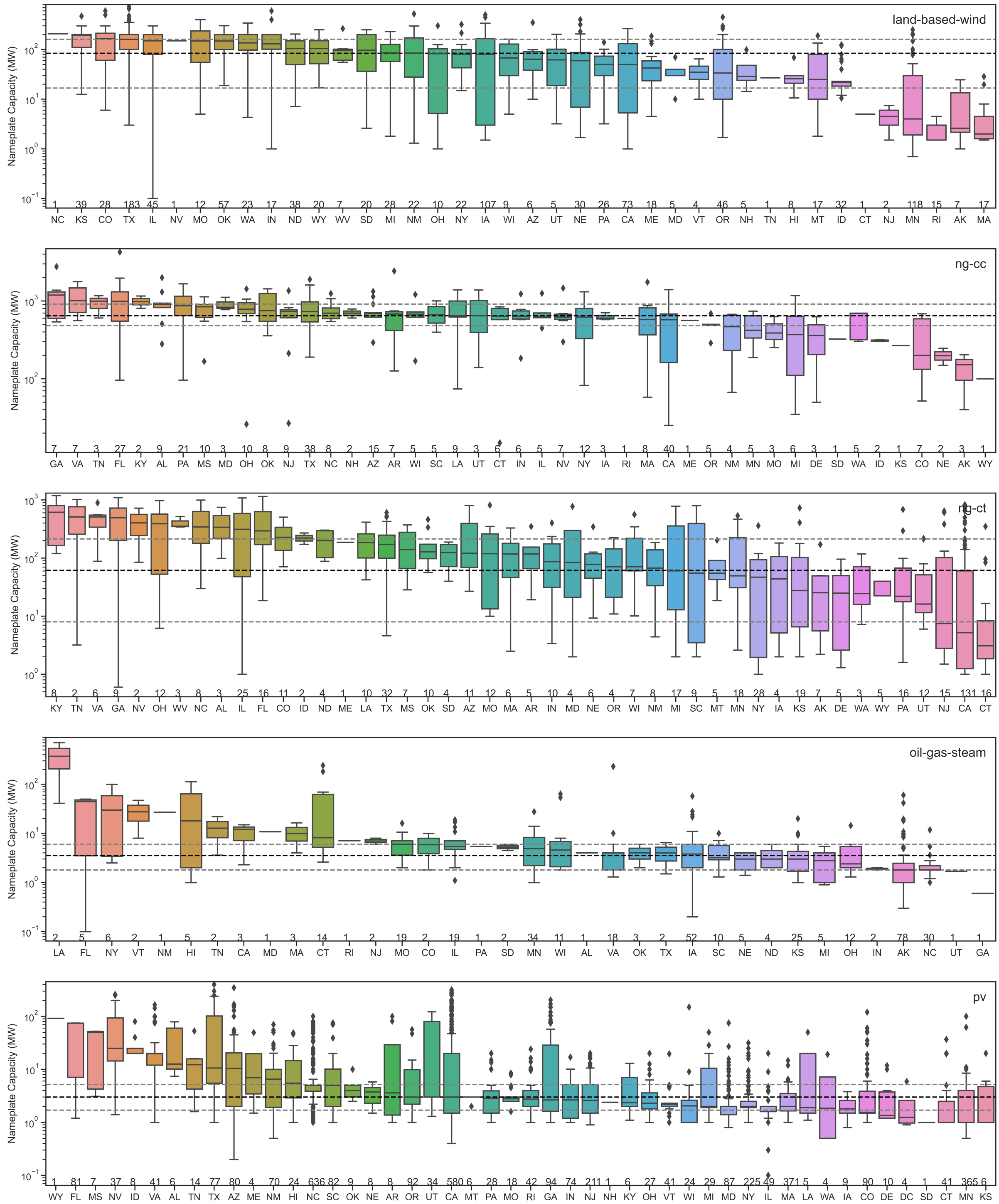

Fig. S6: The nameplate capacities of the existing fleet of land-based wind, natural gas combined cycle, natural gas combustion turbine, oil gas steam turbine, and photovoltaics in each state. The box plots show the median, the 25-75% range (box), and data 1.5 times the interquartile range above and below the box (whiskers). The dashed lines represent the median and the 25-75% range at the national level. The sample size (n) of each technology and state is listed above the state names. Data source: [5].

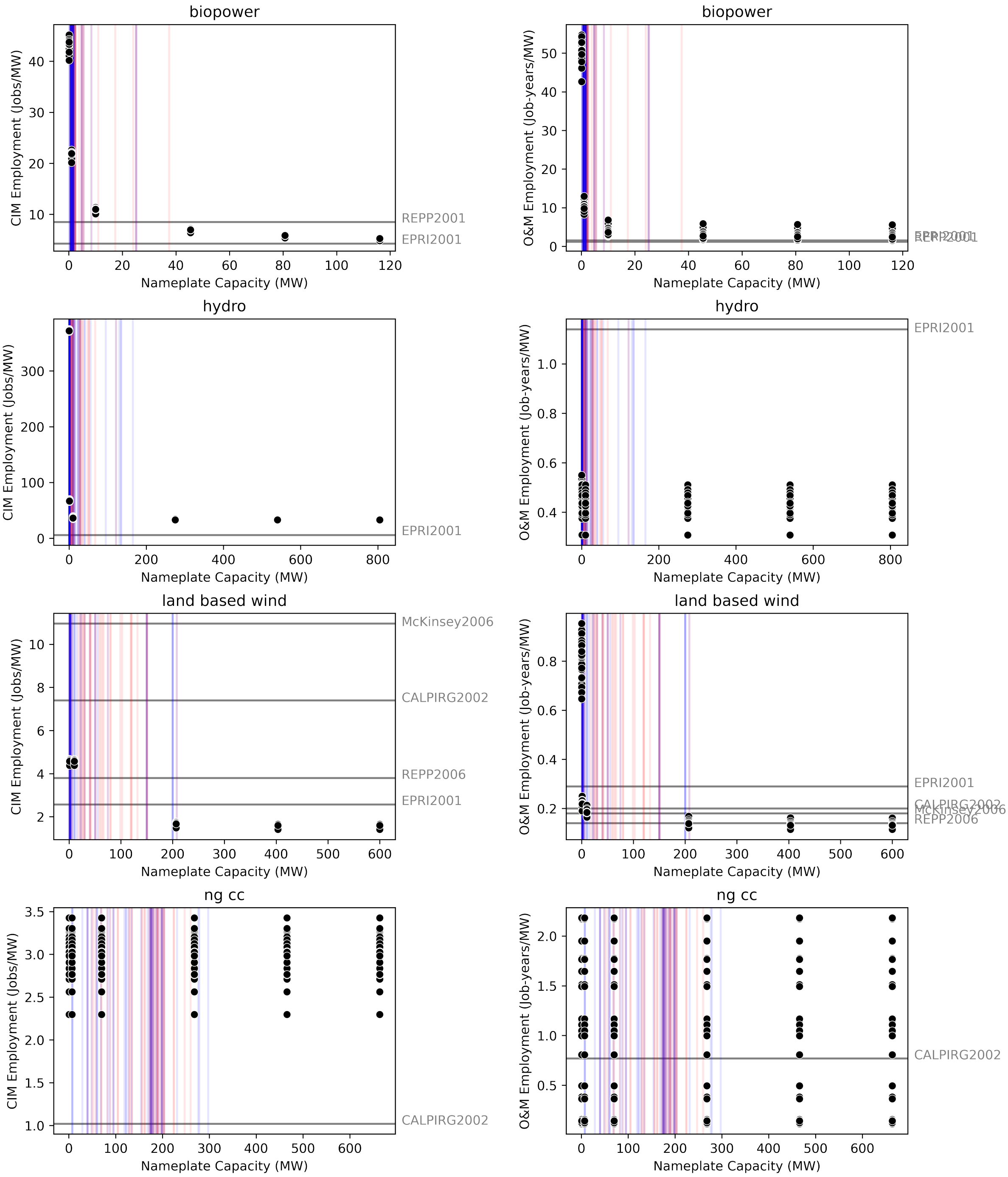

Fig. S7: The CIM and O&M employment output from the JEDI models for biopower, hydro, land-based wind, and natural gas combined cycle. The vertical lines represent the median (red) and mode (blue) of the 50 state-level capacity data. The horizontal lines represent the job creation data in the literature [6].

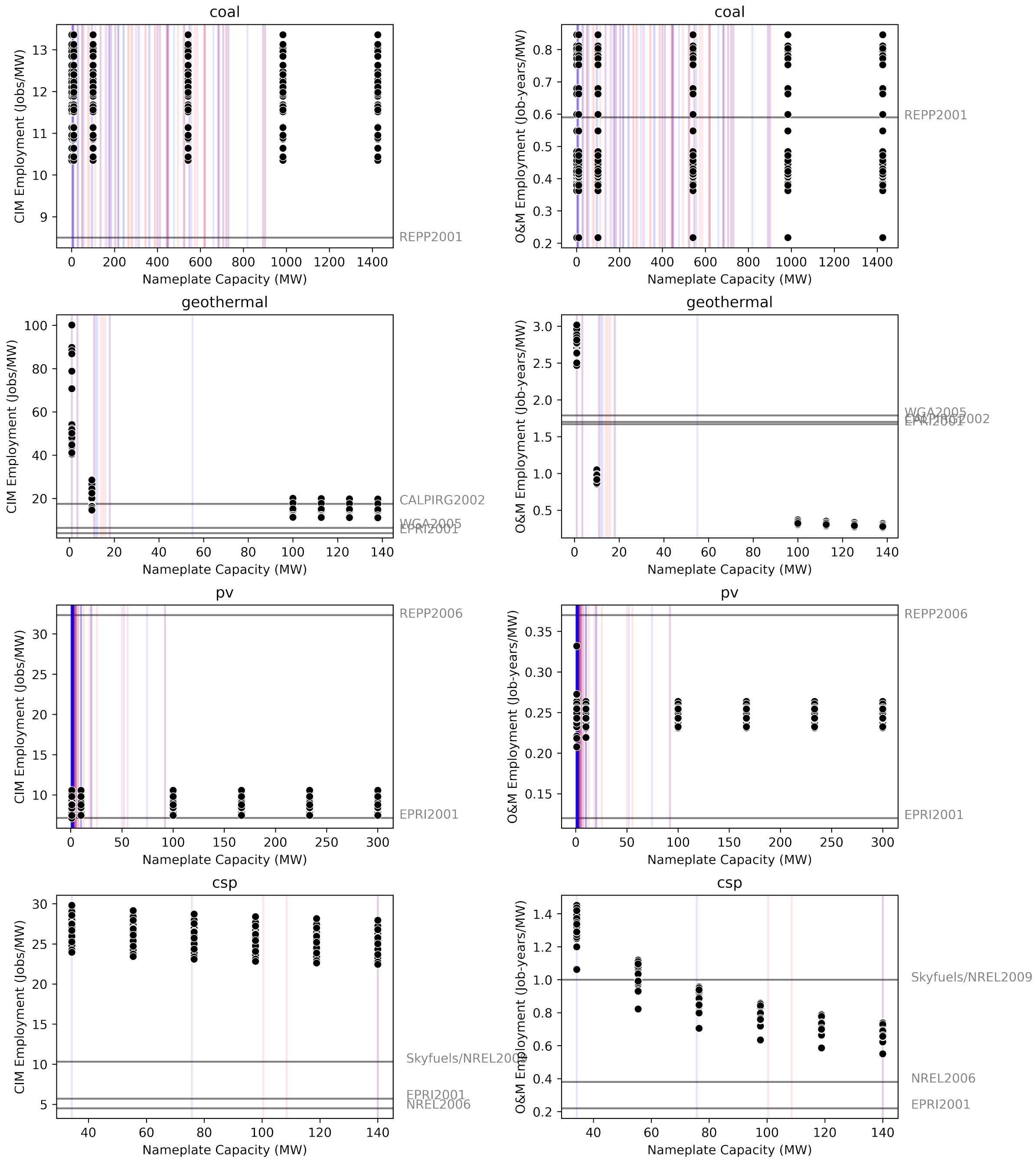

Fig. S8: The CIM and O&M employment output from the JEDI models for coal, geothermal, photovoltaics (utility), and concentrated solar. The vertical lines represent the median (red) and mode (blue) of the 50 state-level capacity data. The horizontal lines represent the job creation data in the literature [6].

3 Modified JEDI Models

The technologies modified include nuclear, oil-gas-steam, natural gas combined cycle with carbon capture (ng-cc-ccs), bioenergy with carbon capture (bio-ccs), batteries, photovoltaic battery (PV battery), pumped storage, hydrogen, and direct air capture (dac). Specific model modifications and corresponding assumptions can be found below.

3.1 Nuclear

The Institute for Sustainable Futures' Calculating Global Energy Sector Jobs report [7] was commonly cited for the employment impacts of nuclear technologies. It is less relevant for this study because its data were sourced from studies in the UK, South Korea, Switzerland, and France. Compiling data from Bechtel Power Corporation, Nuclear Energy Institute, and Dominion Energy, Kenley et al. conducted an employment impact analysis for achieving the "50000 MWe production goal" in the US [8]. This study assumed that all equipment requirements were fulfilled by US suppliers. The Nuclear Energy Institute's employment reports from Illinois in 2014 [9], Ohio [10], New York [11], and Texas [12] in 2015, Connecticut [13] and Minnesota [14] in 2017, and Washington [15] and Nebraska [16] in 2018 are considered. These results (excluding the Ohio report which also included induced jobs) are used to calculate the average job multipliers in job-year per MW. Although they are reported in sectoral details, these reports do not separate jobs created during the construction and operation phases. Some reports did not specify which of the direct, indirect, or induced jobs are included in the results.

Alternatively, the coal JEDI model was used due to the similarity in producing steam to power turbine generators. The overall supply chain assumptions for the power generation facilities were assumed to follow the coal model, although constructing a nuclear power plant may require more jobs in higher-skilled sectors, e.g., engineering, and professional services. The default capacity, capital costs, and non-fuel operational costs (Tables S3-S5) were based on the 2156W Advanced Nuclear (Case 11) in the EIA Capital Cost and Performance Characteristic Estimates for Utility Scale Electric Power Generating Technologies 2020 report ("EIA Technology Report") [17]. The cost of uranium was based on the EIA 2020 Annual Energy Outlook [18] and was assumed to not be produced locally in any state [19].

Table S3: Project descriptive data for the modified nuclear JEDI model.

**Project Descriptive Data**	
Project Size - Nameplate Capacity (MW)	2156
Heat Rare (Btu per kWh)	10608
Plant Construction Cost (\$/kW)	\$ 6,041.00
Fixed Operations and Maintenance Cost (\$/kW)	\$ 121.64
Variable Operations and Maintenance Cost (\$/MWh)	\$ 2.37
Money Value (Dollar Year)	2019

Table S4: Annual operating and maintenance costs for the modified nuclear JEDI model.

**Fixed Costs**	**Cost Per kW**
Labor	$ 13.80
Materials	$ 67.26
Services	$ 40.58
Fixed Subtotal	$ 121.64
**Variable Costs**	**Cost Per MWh**
Ash/sludge disposal	$ 0.17
Water	$ 1.81
Catalysts & chemicals	$ 0.39
Variable Subtotal	$ 2.37
Fuel Cost	$ 7.75

The sensitivity analysis on nuclear employment is based on the Low Nuclear and CCS Costs 100% by 2035 ReEDS Standard Scenario [2] because of its larger scale nuclear deployment. The employment numbers used in this sensitivity analysis do not account for productivity increases. Figure S9 shows the employment outcome using the JEDI and NEI methods by technology. The NEI method reported 1.83 $\pm$ 0.55 times ($P<0.001$) the jobs in the JEDI method. The former is unable to capture the temporal nature of construction jobs and thus might overestimate the amount. In 2050, nuclear jobs take up 8.7% of total jobs with the JEDI method, while it is 18.4% with the NEI method. Figure S10 shows the sectoral breakdown of such employment impacts. Notably, the NEI method resulted in more miscellaneous services jobs, which would ultimately skew the gender impact towards women. This is complicated by the lack of information in NEI reports regarding the different scopes of analysis and the separation of construction versus operation jobs. For simplicity's sake and considering the consistency of this analysis, the JEDI model was used.

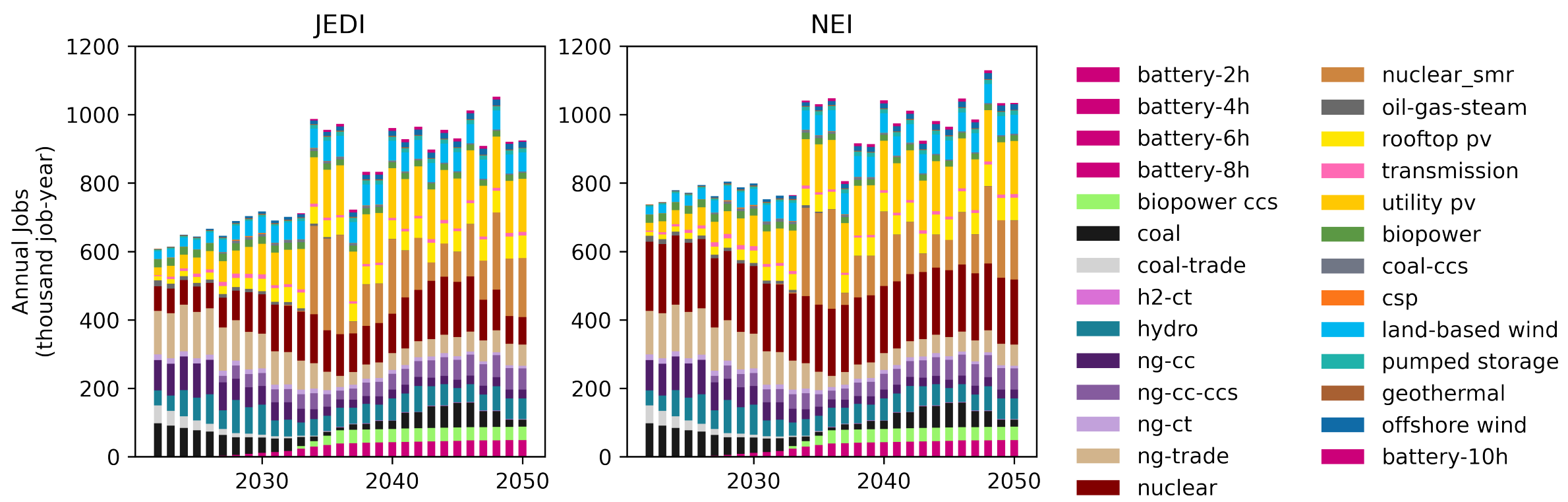

Fig. S9: Modelled electricity sector employment during 2022-2050 in the United States under the 100% reduction by 2035 with Low Nuclear and CCS Costs sensitivity. The nuclear costs from the modified JEDI model and the NEI reports are shown. Data source: own calculations.

Table S5: Construction costs for the modified nuclear JEDI model.

**Facility and Equipment**	
Power Generation	$ 2,709,850,000.00
General facilities	$ 3,577,002,000.00
Plant Equipment	$ 1,517,516,000.00
Facility and Equipment Subtotal	$ 7,804,368,000.00
**Labor**	
Construction Labor	$ 1,956,512,000.00
Project management	$ 102,974,000.00
Labor Subtotal	$ 2,059,486,000.00
Construction Subtotal	$ 9,863,854,000.00
**Other Costs**	
Engineering, Procurement, Construction Management	$ 986,385,000.00
Land	$ 1,980,000.00
Grid intertie	$ 2,772,000.00
Owner costs	$ 2,170,048,000.00
Other Subtotal	$ 3,161,185,000.00
Total	$ 13,025,040,000.00

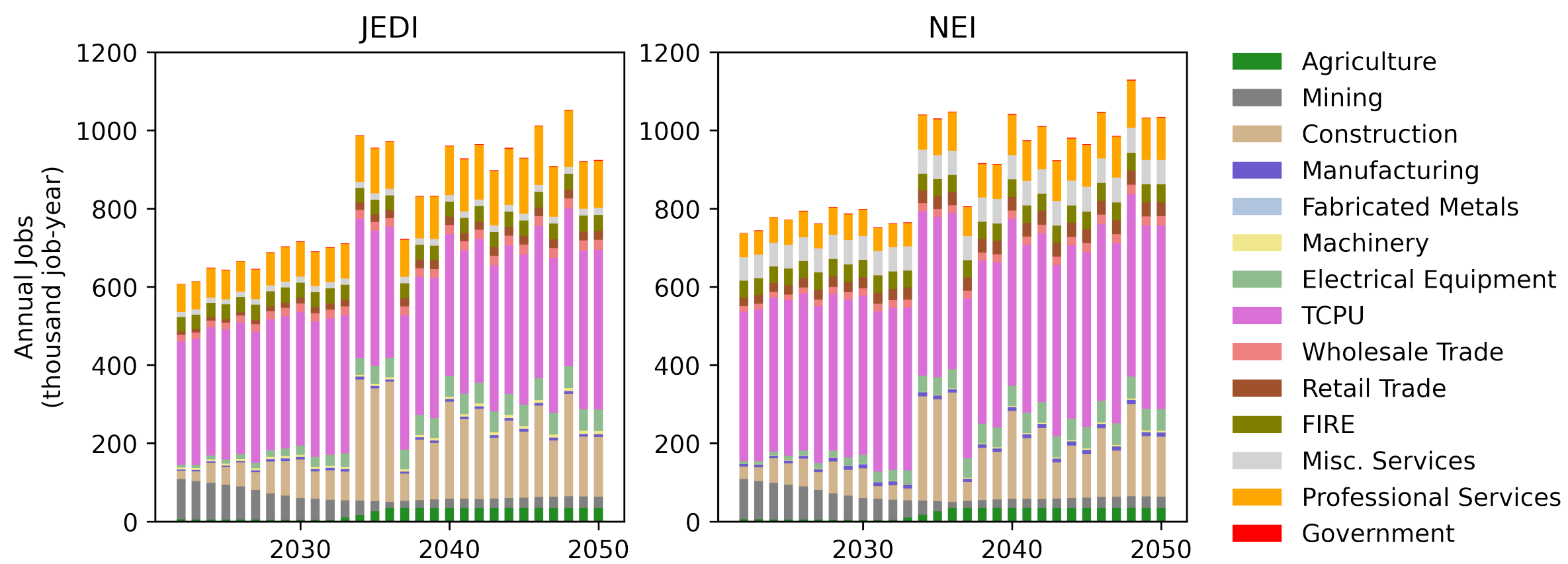

Fig. S10: Modelled sectoral employment during 2022-2050 in the United States under the 100% reduction by 2035 with Low Nuclear and CCS Costs sensitivity. The nuclear costs from the modified JEDI model and the NEI reports are shown. Data source: own calculations.

3.2 Nuclear SMR

The employment for nuclear SMR was calculated similarly to the nuclear JEDI model. The capital and operational cost data of nuclear SMR are based on the 12 x 50MW Small Modular Reactor (Case 12) in the EIA Capital Cost and Performance Characteristic Estimates for Utility Scale Electric Power Generating Technologies 2020 report ("EIA Technology Report") [17]. The default capacity, capital costs, and non-fuel operational costs are in Tables S6-S8.

Table S6: Project descriptive data for the modified nuclear SMR JEDI model.

**Project Descriptive Data**	
Project Size - Nameplate Capacity (MW)	600
Heat Rare (Btu per kWh)	10608
Plant Construction Cost ($/kW)	$ 6,191.00
Fixed Operations and Maintenance Cost ($/kW)	$ 95.00
Variable Operations and Maintenance Cost ($/MWh)	$ 3.00
Money Value (Dollar Year)	2019

Table S7: Annual operating and maintenance costs for the modified nuclear SMR JEDI model.

**Fixed Costs**	**Cost Per kW**
Labor	$ 13.80
Materials	$ 50.64
Services	$ 30.56
Fixed Subtotal	$ 95.00
**Variable Costs**	**Cost Per MWh**
Ash/sludge disposal	$ 0.21
Water	$ 2.30
Catalysts & chemicals	$ 0.49
Variable Subtotal	$ 3.00
Fuel Cost	$ 7.75

Table S8: Construction costs for the modified nuclear SMR JEDI model.

**Facility and Equipment**	
Power Generation	$ 713,196,000.00
General facilities	$ 1,355,072,000.00
Plant Equipment	$ 463,577,000.00
Facility and Equipment Subtotal	$ 2,531,846,000.00
**Labor**	
Construction Labor	$ 575,795,000.00
Project management	$ 30,305,000.00
Labor Subtotal	$ 606,100,000.00
Construction Subtotal	$ 3,137,946,000.00
**Other Costs**	
Engineering, Procurement, Construction Management	$ 313,794,000.00
Land	$ 1,155,000.00
Grid intertie	$ 2,772,000.00
Owner costs	$ 258,881,000.00
Other Subtotal	$ 576,602,000.00
Total	$ 3,714,548,000.00

3.3 Oil-gas-steam

The general cost data for oil-gas-steam was assumed to resemble the coal JEDI model. The cost of fuel was calculated as the average of distilled fuel oil and residual fuel oil [18]. The local share of oil was based on the historical crude oil production at the state level (Table 14, Production of Crude Oil by PAD District and State) [20]. Due to the small percentage (¡1%) of oil consumed for power generation [21] in the US, any state that produced more than 100 thousand barrels in 2020 was assumed to provide 100% of the fuel locally. Otherwise, the local share was assumed to be zero. The heat rate of oil as power generating fuel is calculated from data provided by the EIA [22, 23],

$$HeatRate_{heatingoil} = \frac{137381Btu}{1gallon} \times \frac{1gallon}{12.76kWh} = 10767\frac{Btu}{kWh} \quad (1)$$

3.4 NG-CC-CCS

The NGCC-CCS model is built as a modified version of the natural gas JEDI model. The capital and non-fuel operational cost data are based on the 430MW Combustion Turbine H Class, Combined-Cycle Single Shaft with 90% CO_2 Capture (Case 9, EIA Technology Report) [17]. The post-combustion CO_2 is assumed to be captured with an amine-based sorbent. The cost for the additional CCS facilities was added under the category of "power generation" facilities. The additional fixed O&M cost (13.50 $/kW) of this technology compared to the one without CCS (Case 8, EIA Technology Report) was attributed to the "fixed payment portion of a long-term service agreement for the carbon capture system equipment". Since the ReEDS model creates new gas-CCS technologies through the retrofitting of gas power plants, the construction costs in this analysis are based on the CCS facilities. According to the ReEDS Standard Scenario report [2], an additional 20% was added to such facility costs. The additional variable O&M cost (3.29 $/kWh) was attributed to the cost of amine sorbents. Details of the costs can be found in Tables S9, S10, and S11.

Table S9: System descriptive data for the modified NG-CC-CCS JEDI model.

**System Descriptive Data**	
Project Size - Nameplate Capacity (MW)	430
Heat Rate (Btu per kWh)	7124
Construction Period (Months)	30
Plant Construction Cost ($/KW)	$ 1415.00
Fixed Operations and Maintenance Cost ($/kW)	$ 16.15
Variable Operations and Maintenance Cost ($/MWh)	$ 6.19
Money Value (Dollar Year)	2020

Table S10: Annual operating and maintenance costs for the modified NG-CC-CCS JEDI model.

**Fixed Costs**	**Cost Per KW**
Labor	$ 6.75
Materials	$ 0.98
Services	$ 8.42
Fixed Subtotal	$ 16.15
**Variable Costs**	**Cost Per MWh**
Routine Turbine Maintenance	$ 2.15
Water	$ 0.15
Catalysts & chemicals	$ 3.59
Variable Subtotal	$ 6.19

3.5 Coal-CCS

The Coal-CCS model is built as a modified version of the coal JEDI model. The capital and non-fuel operational cost data are based on the 650MW Ultra-Supercritical Coal with 90% CO_2 Capture (Case 3, EIA Technology Report) [17]. The post-combustion CO_2 is assumed to be captured with an amine-based sorbent. The cost for the additional CCS facilities was added under the category of "power generation" facilities. The additional fixed O&M cost (18.96 $/kW) of this technology compared to the one without CCS (Case 1, EIA Technology Report) was attributed to the "fixed payment portion of a long-term service agreement for the carbon capture system equipment". Since the ReEDS model creates new coal-CCS technologies through the retrofitting of coal power plants, the construction costs in this analysis are based on the CCS facilities. According to the ReEDS Standard Scenario report [2], an additional 20% was added to such facility costs. The additional variable O&M cost (6.48 $/kWh) was attributed to the cost of amine sorbents. Details of the costs can be found in Tables S12, S13, and S14.

Table S11: Retrofit construction costs for the modified NG-CC-CCS JEDI model.

**Facility and Equipment**	
Plant Equipment	$ 478,244,000.00
**Labor and Management**	
Construction Labor	$ 13,200,000.00
Project management (construction and owner's)	$ 37,842,000.00
**Other Costs**	
Permitting Fees	$ 49,144,000.00
Sales Tax (Materials & Equipment Purchases)	$ 29,890,000.00
Total Project Cost	$ 608,321,000.00

Table S12: System descriptive data for the modified Coal-CCS JEDI model.

**System Descriptive Data**	
Project Size - Nameplate Capacity (MW)	650
Heat Rate (Btu per kWh)	12507
Construction Period (Months)	55
Plant Construction Cost ($/KW)	$ 2639.00
Fixed Operations and Maintenance Cost ($/kW)	$ 42.06
Variable Operations and Maintenance Cost ($/MWh)	$ 10.14
Money Value (Dollar Year)	2019

Table S13: Annual operating and maintenance costs for the modified Coal-CCS JEDI model.

**Fixed Costs**	**Cost Per KW**
Labor	$ 19.18
Materials	$ 12.29
Services	$ 10.58
Fixed Subtotal	$ 42.06
**Variable Costs**	**Cost Per MWh**
Ash/sludge disposal	$ 0.26
Water	$ 2.80
Catalysts & chemicals	$ 7.08
Variable Subtotal	$ 10.14

Table S14: Retrofit construction costs for the modified Coal-CCS JEDI model.

**Facility and Equipment**	
Power Generation	$ 47,675,000.00
General facilities	$ 214,938,000.00
Plant Equipment	$ 1,175,275,000.00
**Labor and Management**	
Construction Labor	$ 96,794,000.00
Project management (construction and owner's)	$ 27,232,000.00
**Other Costs**	
Legal and Advisory	$ 153,168,000.00
Total Project Cost	$ 1,715,383,000.00

3.6 Bioenergy-CCS

The bioenergy model was used to create the bioenergy-CCS model. There's limited cost and performance information on a bioenergy-CCS unit with a high capture rate. This work uses data from the Techno-economic Cycle Analysis of Bio-Energy with Carbon Capture and Storage (BECCS) Baseline report developed by NETL, which uses the Cansolv amine-based flue gas carbon capture technology [24]. The additional cost of a CCS unit is added to the bioenergy JEDI model with a unit of 650MW. The cost of a CCS unit is calculated from the net of the facility with CCS (PA3) and the facility without CCS (PN3). While the NETL report currently only provides cost data for bioenergy generation with a mixed feedstock, the PA3 and PN3 facilities are the ones with the highest biomass composition. Tables S15, S16, and S17 show the cost data used in the final bioenergy-CCS model.

Table S15: System descriptive data for the modified bioenergy-CCS JEDI model.

**System Descriptive Data**	
Project Size (MW)	650
Heat Rate (Btu/kWh)	16759
Money Value (Dollar Year)	2018

Table S16: Construction cost for the modified bioenergy-CCS JEDI model.

**Facility and Equipment**	
Plant Equipment	
Feedstock Handling and Preparation	$ 65,632,000.00
Steam Turbine and Auxiliaries	$ 191,676,000.00
Boiler and Air Quality Control	$ 804,821,000.00
General Facilities	$ 269,781,000.00
Equipment Subtotal	$ 1,331,910,000.00
**Labor**	
Construction Labor	$ 157,413,000.00
Labor Subtotal	$ 157,413,000.00
Construction Subtotal	$ 1,489,323,000.00
**Other Costs**	
Balance of Plant	$ 118,490,000.00
Construction Insurance	$ 265,497,000.00
Permitting, Legal, Commissioning and Development	$ 484,765,000.00
Other Subtotal	$ 868,753,000.00
Total	$ 2,358,076,000.00

Table S17: Annual operating and maintenance cost for the modified bioenergy-CCS JEDI model.

**Fixed Costs**	**Per kW**
Labor	
Plant Management	$ 1.82
Administration	$ 3.59
Equipment/Parts	$ 12.00
Insurance	$ 44.60
Fixed Subtotal	$ 62.01
**Variable Costs**	**Per MWh**
Labor	
Operators	$ 0.33
Fuel Handlers	$ 0.05
Maintenance Staff	$ 0.25
Labor Subtotal	$ 0.63
Maintenance Materials	$ 0.92
Variable Subtotal	$ 1.55
Consumables Costs	
Chemicals	$ 0.44
Water	$ 0.05
Solids/Ash Disposal	$ 0.18
Ammonia	$ 0.36
Consumables Subtotal	$ 1.03
Subtotal O&M costs	
Feedstock Cost	$ 40.77

3.7 Batteries and PV-battery

The photovoltaic JEDI model was assumed to be a close fit for the batteries and PV-battery modifications due to the modular nature of manufacturing and the automated operational processes. While the battery industry has followed the cost reduction trajectory of the photovoltaics industry, the former can be more difficult due to its more complex material composition compared to the more widely available silicon [25]. This may also require higher-skilled labor in sectors such as chemicals. The capital and non-fuel operational costs of the PV battery were based on the reported values for the 150 MWAC Solar Photovoltaic with Battery Energy Storage System (Case 25, EIA Technology Report) [17]. Those costs for the batteries were based on the Lithium-ion LFP (Appendix 1, pg 87) data in the Pacific Northwest National Laboratory's (PNNL) 2020 Grid Energy Storage Technology Cost and Performance Assessment report [26]. Details of these values can be found in Tables S18, S19, and S20.

Table S18: Project descriptive data for the modified battery (2h, 4h, 6h, 8h, and 10h) and PV-battery JEDI models.

Project Descriptive Data	2h	4h	6h	8h	10h	PV
System Application	Utility	Utility	Utility	Utility	Utility	Utility
Total Project Size - DC Nameplate Capacity (kW)	10000	10000	10000	10000	10000	150000
Base Installed System Cost ($/kWDC)	$ 922.00	$ 1,643.00	$ 2,355.00	$ 3,063.00	$ 3,767.00	$ 1,755.00
Annual Direct Operations and Maintenance Cost ($/kW)	$ 12.50	$ 24.55	$ 36.58	$ 48.60	$ 60.61	$ 32.17
Money Value (Dollar Year)	2020	2020	2020	2020	2020	2019

Table S19: Intallation costs (Cost per kW) of for the modified battery (2h, 4h, 6h, 8h, and 10h) and PV-battery JEDI models.

**Materials & Equipment**	**2h**	**4h**	**6h**	**8h**	**10h**	**PV**
Mounting (rails, clamps, fittings, etc.)	$ 86.00	$ 160.00	$ 234.00	$ 312.00	$ 380.00	$ 359.91
Modules	$ 352.00	$ 696.00	$ 1,032.00	$ 1,368.00	$ 1,700.00	$ 481.00
Electrical (wire, connectors, breakers, etc.)	$ 81.00	$ 81.00	$ 81.00	$ 81.00	$ 81.00	$ 605.94
Inverter	$ 104.00	$ 188.00	$ 270.00	$ 352.00	$ 430.00	$ 96.39
Subtotal	$ 623.00	$ 1,125.00	$ 1,617.00	$ 2,113.00	$ 2,591.00	$ 1,543.25
**Labor**						
Installation	$ 25.00	$ 25.00	$ 25.00	$ 25.00	$ 25.00	$ 28.01
Subtotal	$ 25.00	$ 25.00	$ 25.00	$ 25.00	$ 25.00	$ 28.01
Total	$ 648.00	$ 1,150.00	$ 1,642.00	$ 2,138.00	$ 2,616.00	$ 1,571.26
**Other Costs**						
Permitting, Fees, and Taxes	$ 124.00	$ 224.00	$ 324.00	$ 424.00	$ 520.00	$ 54.51
Other Costs	$ -	$ -	$ -	$ -	$ -	$ 45.79
Business Overhead	$ 150.00	$ 268.00	$ 390.00	$ 504.00	$ 620.00	$ 83.58
Subtotal	$ 274.00	$ 492.00	$ 714.00	$ 928.00	$ 1,140.00	$ 183.89
Subtotal	$ 922.00	$ 1,642.00	$ 2,356.00	$ 3,066.00	$ 3,756.00	$ 1,755.15
Total	$ 922.00	$ 1,642.00	$ 2,356.00	$ 3,066.00	$ 3,756.00	$ 1,755.15

Table S20: Annual operating and maintenance costs (Cost Per kW)in the modified battery (2h, 4h, 6h, 8h, and 10h) and PV-battery JEDI models.

**Labor**	**2h**	**4h**	**6h**	**8h**	**10h**	**PV**
Technicians	$ 2.24	$ 4.03	$ 5.80	$ 7.56	$ 9.31	$ 11.07
Subtotal	$ 2.24	$ 4.03	$ 5.80	$ 7.56	$ 9.31	$ 11.07
**Materials and Services**						
Materials & Equipment	$ 10.25	$ 20.50	$ 30.75	$ 41.00	$ 51.25	$ 20.21
Services	$ 0.01	$ 0.02	$ 0.03	$ 0.04	$ 0.05	$ 0.89
Subtotal	$ 12.50	$ 24.55	$ 36.58	$ 48.60	$ 60.61	$ 32.17
Total	$ 12.50	$ 24.55	$ 36.58	$ 48.60	$ 60.61	$ 32.17

3.8 Renewable Energy Combustion

Renewable energy combustion generators were simply assumed to be hydrogen generators. The hydrogen JEDI model was modified from the natural gas model. Given the potential of converting natural gas power plants into hydrogen facilities, the majority of the model set up remains the same and the efficiency is based on the Hydrogen Power Generation – Challenges and Prospects for 100% Hydrogen Gas Turbines report for the UK CCS Research Centre [27]. The heat rate of hydrogen was based on the GE Hydrogen for Power Generation report [28]. The cost of hydrogen in the US is based on the DOE Cost of Electrolytic Hydrogen Production with Existing Technology report [29]. Table S21 shows the modifications.

Table S21: Technology and economic data for the modified hydrogen model.

**System Descriptive Data**	
Project Size - Nameplate Capacity (MW)	2200
Heat Rate (Btu per kWh)	5686.9
Cost of Fuel ($/mmbtu)	$ 52.77
Money Value (Dollar Year)	2020

3.9 Direct Air Capture

Given the lack of at-scale DAC plant data, detailed cost information (both early and Nth) from Carbon Engineering's report [30] is in the structure of a natural gas JEDI model. This assumes that the economic structure of a developed carbon dioxide supply chain may be similar to the natural gas supply chain. The ReEDS model 2022 Standard Scenarios deploys electricity-powered DAC facilities [2]. Since the detailed cost data from the Carbon Engineering study [30] only describes the partially electricity-powered DAC, the cost data of fully electricity-powered DAC are derived from [31], which was also used by the ReEDS analysis.

The electricity capacity differences between the two high-temperature aqueous solution-based DAC technologies are reported in Table S22 and the 175MW capacity is adapted in this analysis to account for the specifications of the ReEDS model. The amounts of carbon dioxide removed through DAC reported by ReEDS (dac_co2_capture_mt) are calculated into electricity demand, using electricity demand data from Fasihi et al. 2019 [31] Table 7 (HT DAC (electrified)), which accounts for technology learning. Although the Fasihi study estimates the CAPEX cost reduction until 2050, we do not include these data because further information is needed to identify the plant components experiencing more cost reduction. Thus, we acknowledge that DAC jobs in this analysis are overestimated. The 2-year construction time is rounded from the 15-month build time of the ClimeWorks Orca plant [4]. Tables S23 and S24 show the assumptions made to the JEDI model.

Table S22: Electric capacities of DAC technologies.

**Parameter**	**Unit**	**Keith 2018**[30]	**Fasihi 2019**[31]
Capacity	ton/a	1000000	1000000
Capacity	ton/h	114.16	114.16
Electricity Demand	kWh/ton	366	1535
Heat Demand	kWh/ton	1460	0
Electricity Capacity	MW	41.8	175.2

Table S23: Construction costs for the modified DAC model.

**Facility and Equipment**	**Early**	**Nth**
Power Generation	$ 385,440,000.00	$260,100,100.00
General facilities	$ 151,900,000.00	$125,800,000.00
Plant Equipment	$ 153,600,000.00	$118,500,000.00
Subtotal	$ 690,940,000.00	$504,400,000.00
**Labor and Management**		
Construction Labor	$ 6,700,000.00	$5,800,000.00
Construction Utilities	$ 88,500,000.00	$68,800,000.00
Subtotal	$ 95,200,000.00	$74,600,000.00
Construction Subtotal	$ 786,140,000.00	$579,000,000.00
**Other Costs**		
Engineering/Design	$ 135,200,000.00	$78,000,000.00
Construction insurance	$ 157,200,000.00	$86,800,000.00
Permitting Fees	$ 48,200,000.00	$35,800,000.00
Other Subtotal	$ 340,600,000.00	$200,600,000.00
Subtotal All Costs (without sales tax)	$ 1,126,740,000.00	$779,600,000.00
Sales Tax (Materials & Equipment Purchases)	$ 43,184,000.00	$31,525,000.00
Total Project Cost	$ 1,169,924,000.00	$811,125,000.00

The sensitivities of DAC employment between the early and Nth plants are explored for the

Table S24: Annual operating and maintenance costs for the modified DAC model.

**Fixed Costs**	**Per kW**
Labor	\$ 48.25
Materials	\$ 6.99
Services	\$ 60.13
Fixed Subtotal	\$ 115.36
**Variable Costs**	**Per MWh**
Routine Turbine Maintenance	\$ 2.45
Water	\$ 3.02
Catalysts & chemicals	\$ 3.02
Variable Subtotal	\$ 8.49

100% by 2035 trajectory and DAC sensitivity case reported by ReEDS. It is the scenario with the most significant DAC deployment. Figure S11 shows the comparison of national electricity job results using early and Nth plant costs. These results are original outputs without productivity considerations. The early plant DAC employment is 1.05 ± 0.07 times (P=0.46) Nth plant employment nationally, thus indicating no significant difference. In 2036 where most DAC jobs are placed, early plant DAC jobs take up 28.68% of total jobs and Nth plant DAC jobs take up 26.56% of total jobs. Figure S12 shows the employment results in Texas, which is the state deploying most of the DAC capacities. The early plant DAC employment is 1.05 ± 0.07 times (P=0.47) the Nth plant jobs in Texas. In 2036, early plant jobs would take up 77.5% of electricity jobs in Texas, while the Nth plant would take up 75.6%. For simplicity's sake, the Nth plant cost is used in this study given it doesn't present significant differences in employment numbers and to account for the technology learning that is expected to occur in the next decade before DAC is deployed in the modeled electricity system.

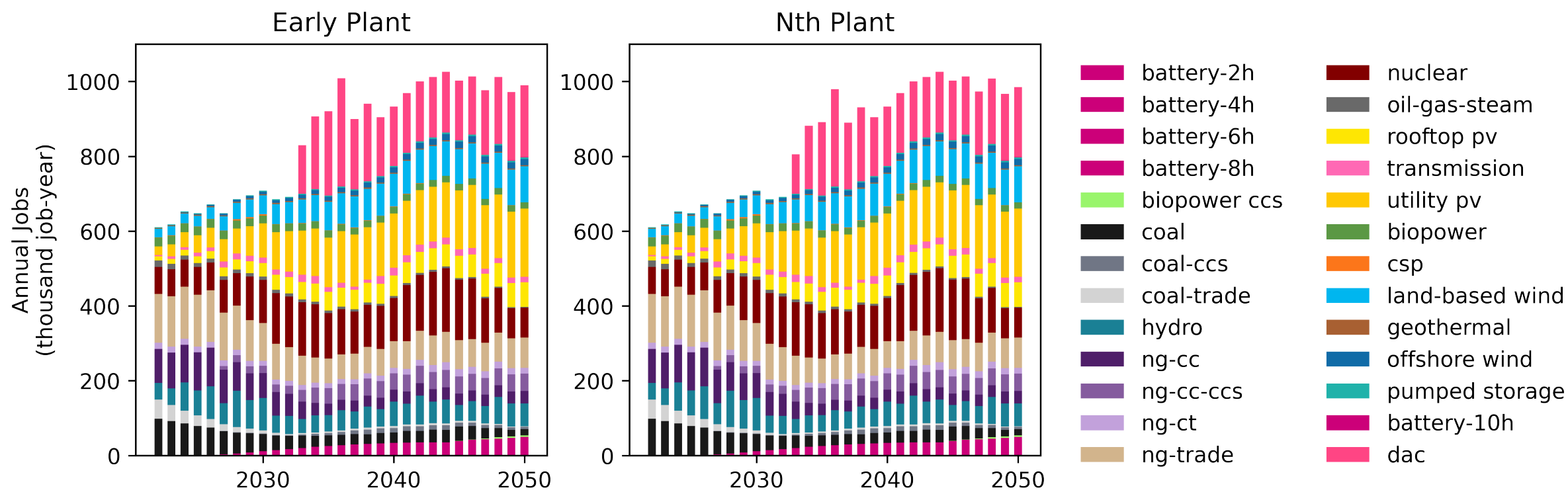

Fig. S11: Modelled absolute electricity sector employment during 2022-2050 in the US under the 100% reduction by 2035 with DAC sensitivity. The early and nth plant DAC costs are shown. Data source: own calculations.

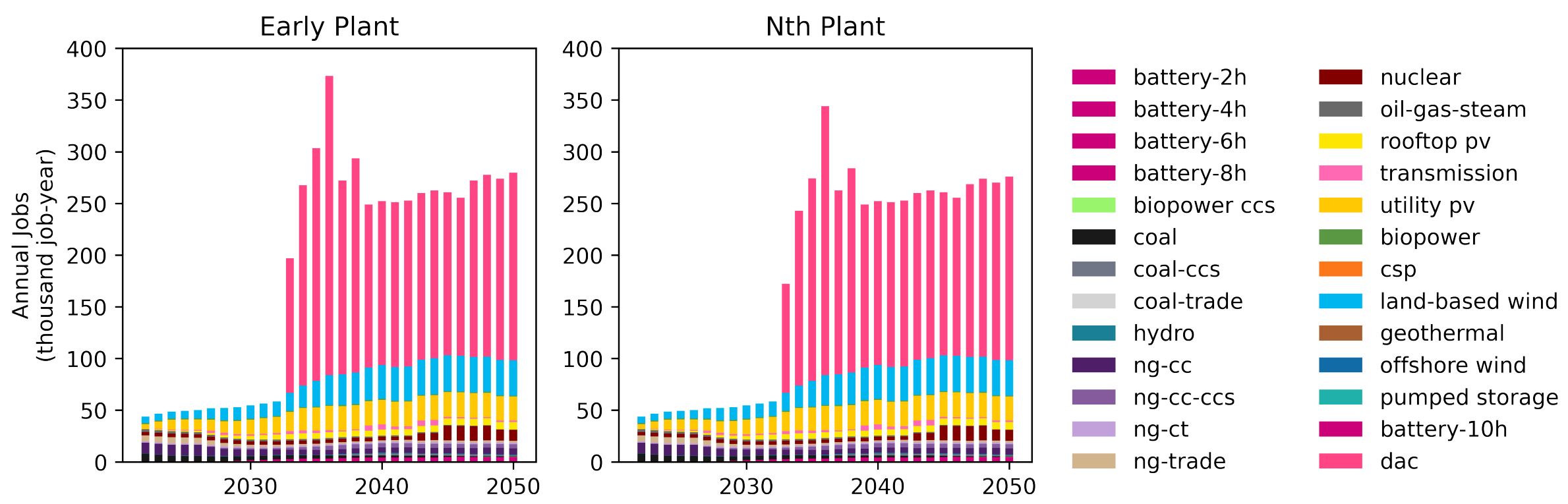

Fig. S12: Modelled absolute electricity sector employment during 2022-2050 in Texas under the 100% reduction by 2035 with DAC sensitivity. The early and nth plant DAC costs are shown. Data source: own calculations.

4 Industrial Crosswalks

Table S25: Industry crosswalk between the O*NET industry categories based on the North American Industry Classification System (NAICS) and the 14 JEDI industry sectors.

JEDI Index	JEDI Industries	NAICS Industries
1	Agriculture	Agriculture_Forestry_Fishing_and_Hunting
2	Mining	Mining_Quarrying_and_Oil_and_Gas_Extraction
3	Construction	Construction
4	Manufacturing	Manufacturing
8	TCPU	Utilities
8	TCPU	Transportation_and_Warehousing
8	TCPU	Information
9	Wholesale Trade	Wholesale_Trade
10	Retail Trade	Retail_Trade
11	FIRE	Finance_and_Insurance
11	FIRE	Real_Estate_and_Rental_and_Leasing
12	Misc. Services	Administrative_and_Support_Services
12	Misc. Services	Educational_Services
12	Misc. Services	Health_Care_and_Social_Assistance
12	Misc. Services	Arts_Entertainment_and_Recreation
12	Misc. Services	Accommodation_and_Food_Services
12	Misc. Services	Other_Services_Except_Public_Administration_
13	Professional Services	Professional_Scientific_and_Technical_Services
13	Professional Services	Management_of_Companies_and_Enterprises
14	Government	

Table S26: Industry crosswalk between the Census industry categories and the 14 JEDI industry sectors.

JEDI Index	JEDI Industries	Census Index	Census
1	Agriculture	3	Agriculture, forestry, fishing and hunting
2	Mining	4	Mining, quarrying, and oil and gas extraction
3	Construction	5	Construction
4	Manufacturing	6	Manufacturing
5	Fabricated Metals	6	Manufacturing
6	Machinery	6	Manufacturing
7	Electrical Equipment	6	Manufacturing
8	TCPU	9	Transportation and warehousing, and utilities:
8	TCPU	12	Information
9	Wholesale Trade	7	Wholesale trade
10	Retail Trade	8	Retail trade
11	FIRE	13	Finance and insurance, and real estate and rental and leasing:
12	Misc. Services	20	Educational services, and health care and social assistance:
12	Misc. Services	23	Arts, entertainment, and recreation, and accommodation and food services:
12	Misc. Services	26	Other services, except public administration
13	Professional Services	16	Professional, scientific, and management, and administrative and waste management services:
14	Government	27	Public Administration

Table S27: Industry crosswalk between the 22 industry sectors in the JEDI photovoltaics module and the 14 main JEDI industry sectors.

PV Industry	PV Index	JEDI Industry
Ag, Forestry, Fish & Hunting	1	Agriculture
Mining	2	Mining
Construction	3	Construction
Construction/Installations - Non Residential	4	Construction
Construction/Installation Residential	5	Construction
Manufacturing	6	Manufacturing
Fabricated Metals	7	Fabricated Metals
Machinery	8	Machinery
Electrical Equip	9	Electrical Equipment
Battery Manufacturing	10	Electrical Equipment
Energy Wire Manufacturing	11	Electrical Equipment
Wholesale Trade	12	Wholesale Trade
Retail trade	13	Retail Trade
TCPU	14	TCPU
Insurance and Real Estate	15	FIRE
Finance	16	FIRE
Other Professional Services	17	Professional Services
Office Services	18	Professional Services
Architectural and Engineering Services	19	Professional Services
Other services	20	Misc. Services
Government	21	Government
Semiconductor (solar cell/module) manufacturing	22	Electrical Equipment

Table S28: Industry crosswalk between the Bureau of Labor Statistics (BLS) industry categories and the 14 JEDI industry sectors.

JEDI Index	JEDI Industries	BLS Industries
1	Agriculture	Agriculture, forestry, fishing, and hunting
2	Mining	Mining, quarrying, and oil and gas extraction
3	Construction	Construction
4	Manufacturing	Nonmetallic mineral products manufacturing
4	Manufacturing	Transportation equipment manufacturing
4	Manufacturing	Wood products manufacturing
4	Manufacturing	Furniture and related product manufacturing
4	Manufacturing	Miscellaneous manufacturing
4	Manufacturing	Nondurable goods manufacturing
5	Fabricated Metals	Primary metals and fabricated metal products manufacturing
6	Machinery	Machinery manufacturing
7	Electrical Equipment	Computers and electronic products manufacturing
7	Electrical Equipment	Electrical equipment and appliances manufacturing
8	TCPU	Transportation and utilities
8	TCPU	Information
9	Wholesale Trade	Wholesale trade
10	Retail Trade	Retail trade
11	FIRE	Financial activities
12	Misc. Services	Education and health services
12	Misc. Services	Leisure and hospitality
12	Misc. Services	Other services
13	Professional Services	Professional and business services
14	Government	Public Administration

5 Local Share of Fuels

5.1 Coal and natural gas

The local shares (Figure S15) of natural gas and coal in each state are calculated from historical ratios of consumption and production data (Figures S13 and S14) from the Energy Information Administration.

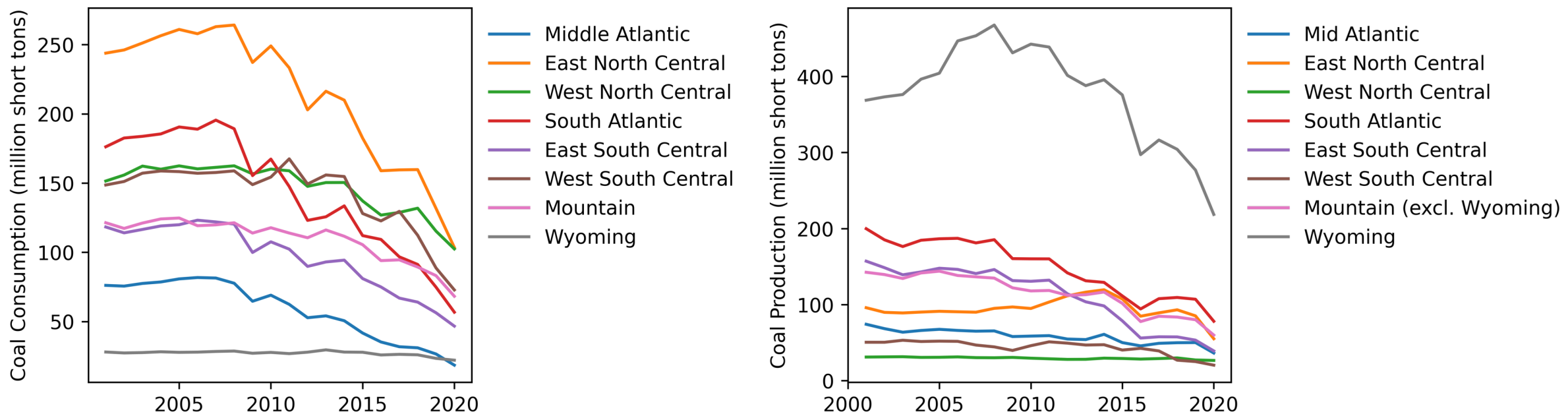

Fig. S13: The historical consumption (left) and production (right) of coal across several regions from 2001 to 2020. Wyoming was the largest producer of coal in the United States. Data source: [32].

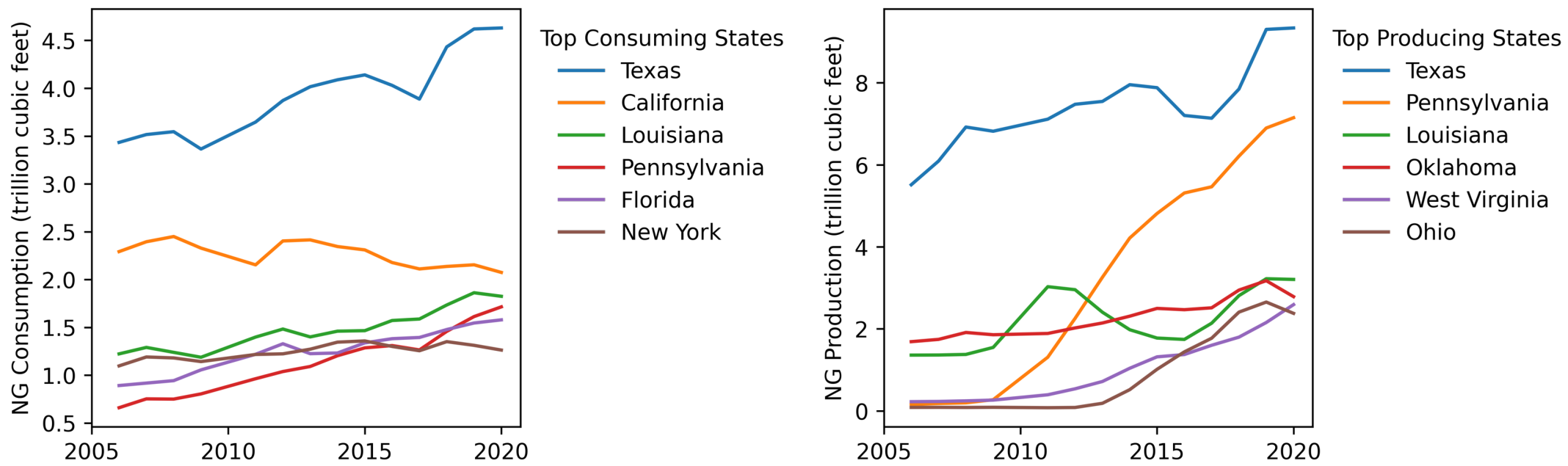

Fig. S14: The historical consumption (left) and production (right) of natural gas across the top consuming and producing states from 2001 to 2020. Texas is the top producing and consuming state in the United States. Data source: [33], Table 2. Natural Gas Production, Transmission, and Consumption by State.

For the share of fuels not produced locally, the supplier states were estimated based on the US Census Commodity Flow Survey [34]. For example, if a state produces 30% of its coal consumption locally, the remaining 70% is attributed to jobs in supplier states in the ratio of its historical coal imports. Figures S16 and S17 below show the flows of coal (15 – Coal) and natural gas (19 - Other coal and petroleum products, not elsewhere classified) in 2017 respectively.

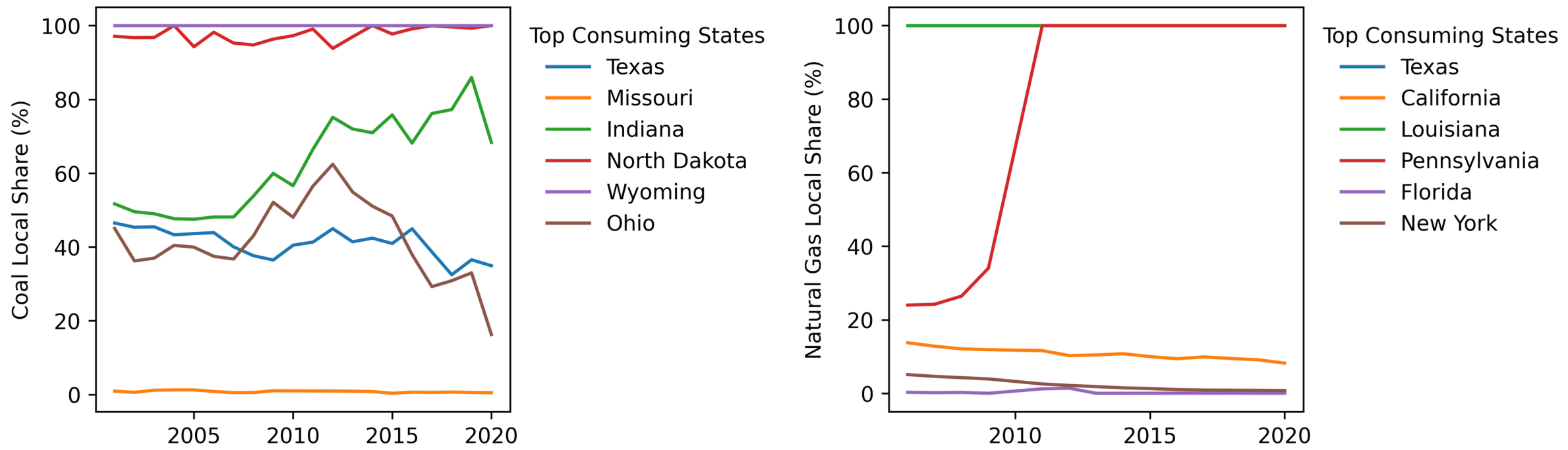

Fig. S15: The historical local shares of coal (left) and natural gas (right) in the top 6 consuming states in 2020. If the state outproduced its consumption, the local share was assumed to be 100%. If the state did not produce the fuel, the local share was zero. Otherwise, the local share was estimated to be the percentage of consumption in production in 2020. Data source: own calculations.

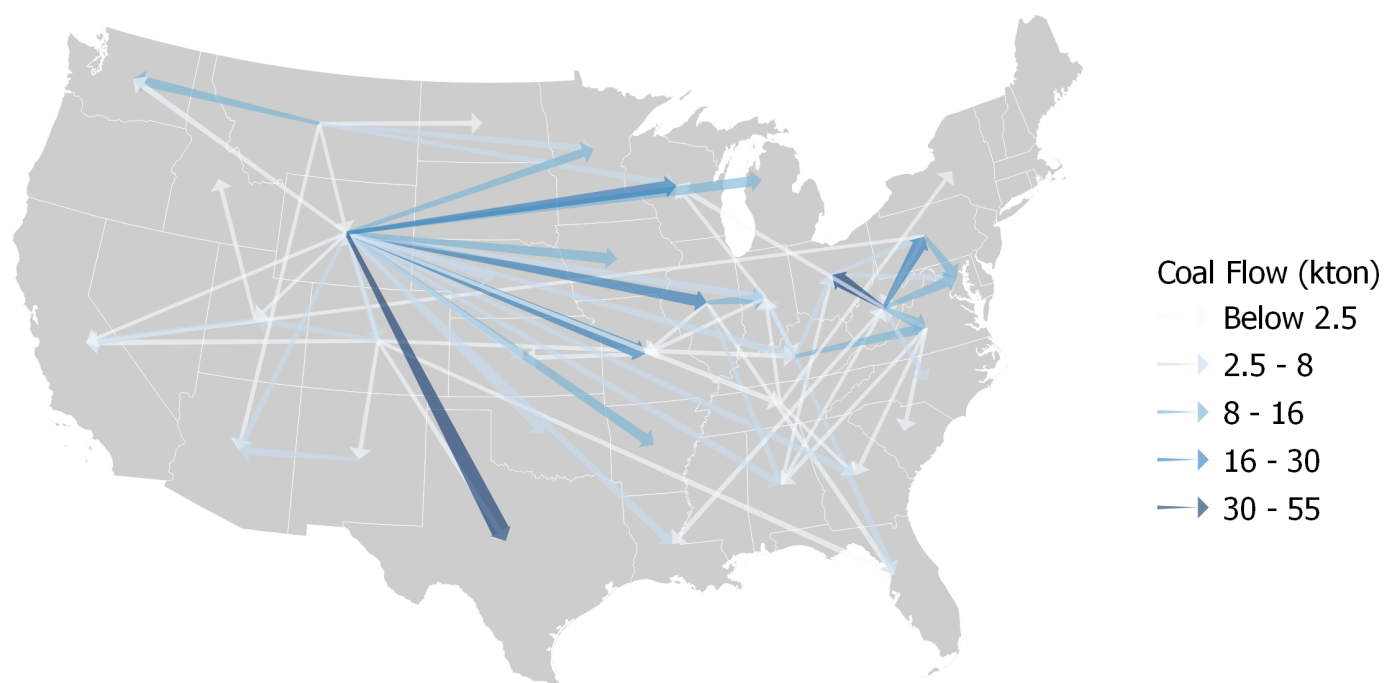

Fig. S16: The flow of coal across US states.

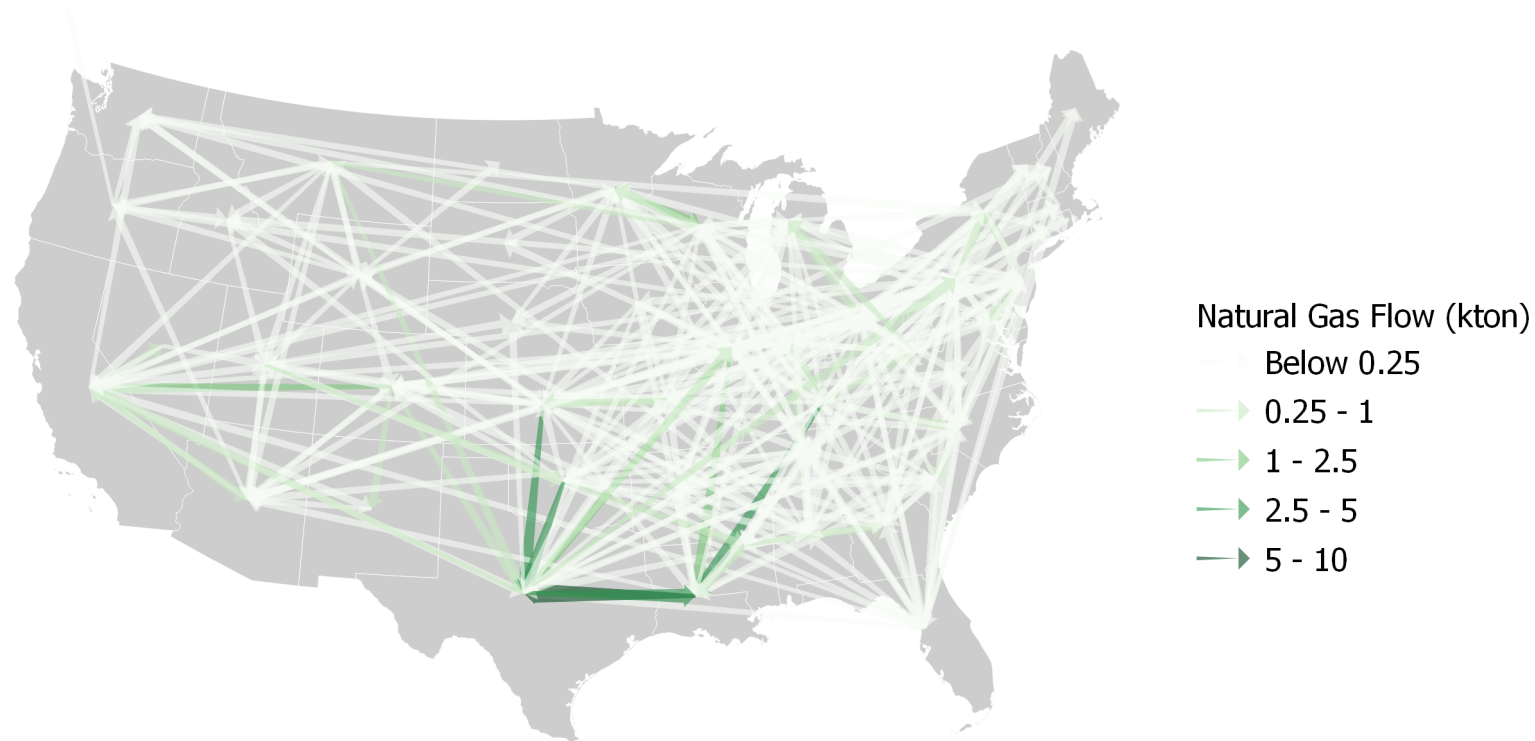

Fig. S17: The flow of natural gas across US states.

5.2 Biomass feedstock

The NREL Biopower Atlas [35] was used to extract the state- and county-level feedstock production (Figure S19) estimate data from the 2016 billion-ton study [36]. The study provided data on the resource availabilities (tonne per year) of several different biopower feedstocks including barley straw, sorghum, and corn stover. The Higher Heating Values (HHV) of feedstocks are based on the Biopower Technical Assessment Table A3-4 Ultimate Analysis Data for Selected Solid Fuels and Biomass Materials [37]. The power generation ability of a feedstock was calculated as follows,

$$Generation[kWh] = \frac{Production_{feedstock}[lb] \cdot HHV_{feedstock}[\frac{Btu}{lb}]}{HeatRate[\frac{Btu}{kWh}]} \quad (2)$$

where the heat rate was 14000 Btu/kWh (based on the JEDI model).

In the ReEDS Mid Case Standard Scenario, the calculated usages of biopower feedstocks generally took up less than 10% of the total amounts produced by each state (Figure S18 - right). Thus, the local shares of the biopower generators were assumed to be 100% across all states. Although this assumption neglects the dynamic merit order mechanism of the market, it simplifies the analysis before more accurate local share data becomes available.

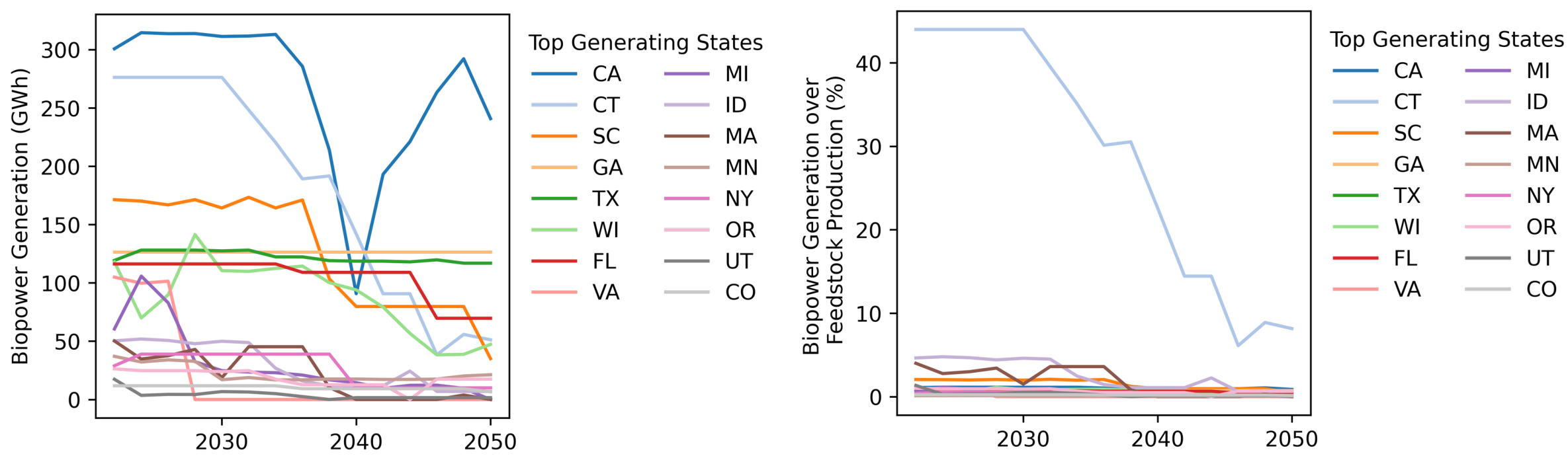

Fig. S18: Biopower generation (left) and the percentage of generation over the state's total biopower feedstock production (right) using the ReEDS Mid Case Standard Scenario. (Data source: [38] and own calculations)

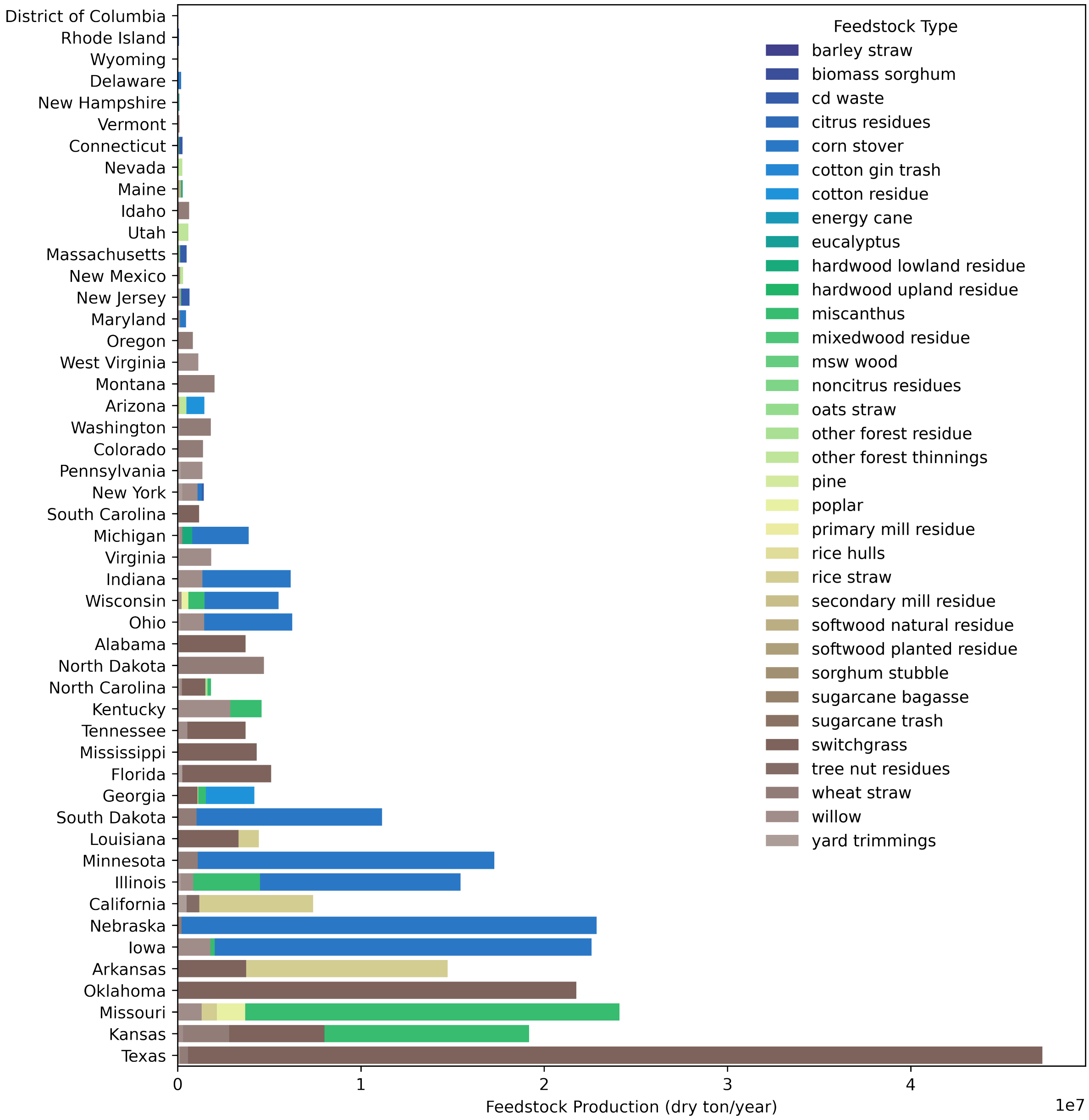

Fig. S19: Biopower feedstock production by feedstock type and state.

6 Demographic Databases

6.1 Productivity

Because the JEDI model results are presented in a static manner, a simple annual 1% productivity increase was used [39]. This assumption is contextualized by historical data from the Bureau of Labor Statistics, which identify an economy-wide labor productivity slowdown from 2010 to 2018 [40]. In order to utilize historical productivity data for this analysis, we conducted a non-linear least square regression with the function,

$$P_t = P_o(1+c)^{t-t_o}$$

where c is the annual productivity increase coefficient, P is the labor productivity index (2012=100), and t is the year.

Figure S20 shows the regression results nationally from 2012 to 2018 (a) and 2012 to 2021 (b). The historical data are from the "Labor Productivity and Real Hourly Compensation, Nonfarm Business Sector, 1973-2022" table [41]. The regression from 2012 to 2018 shows slowed growth, while the regression from 2012 to 2021 indicates faster growth, especially from 2019. Given the uncertainty during such a short period, we adopted the 1% value used in literature [39].

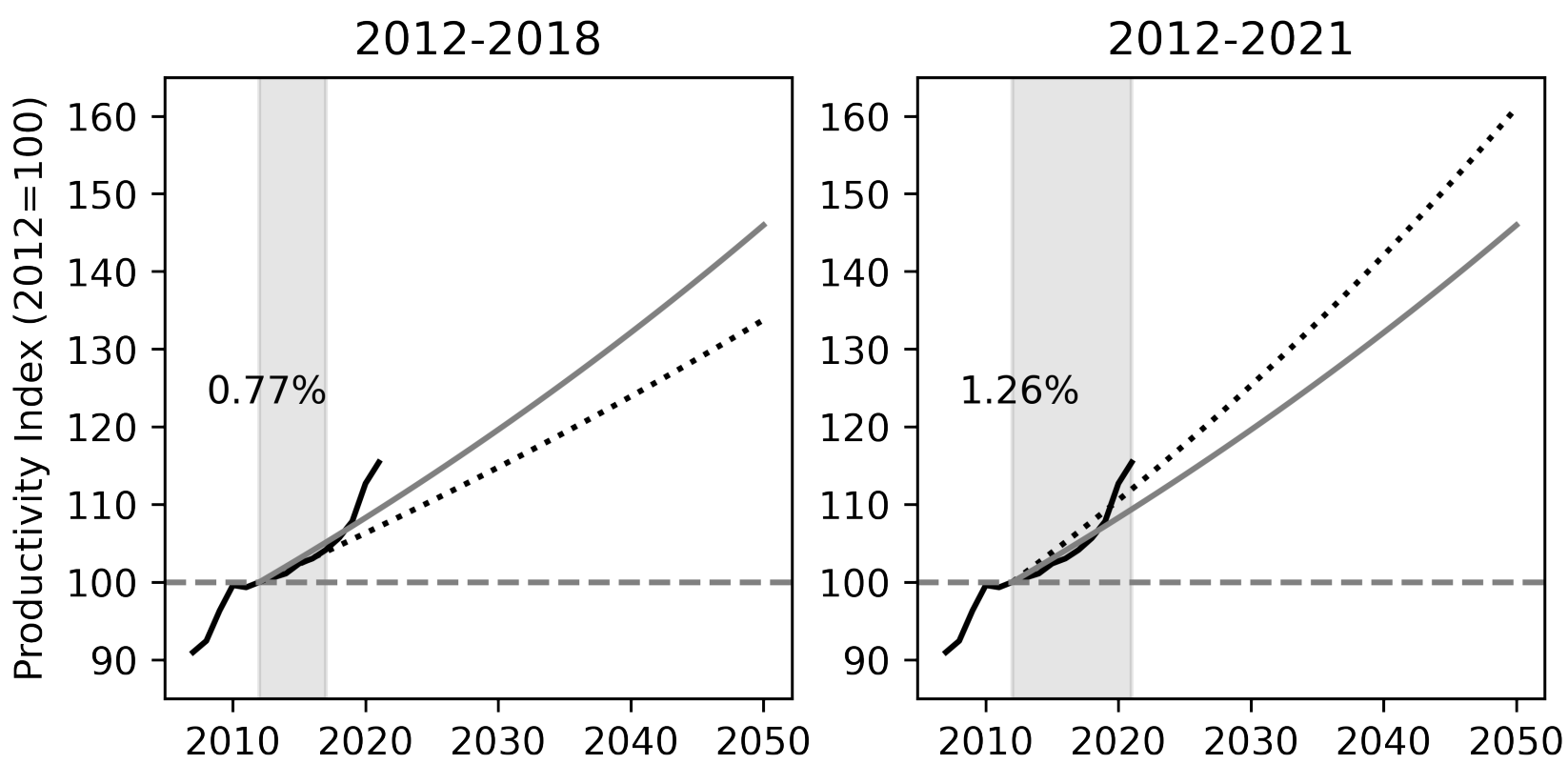

Fig. S20: US national labor productivity trends between 2007-2021 with regression between 2012-2018 (left) and 2012-2021 (right). The black lines are historical data. The gray line projections assume a 1% annual productivity increase. The gray area highlights the time frame when the non-linear least square regression is conducted. The value shown in the figure is the annual productivity increase calculated from the regression. The dotted black lines are future productivity projections using the calculated coefficient. Data source: [41] and own calculations.

Figure S21 shows the state-level productivity historical and regression data. The historical data is from the "Private nonfarm productivity and costs by state and region - May 26, 2022" table [41]. West coast states show faster productivity increases than most. Nevada, Louisiana, Missouri, and Delaware showed a productivity decline.

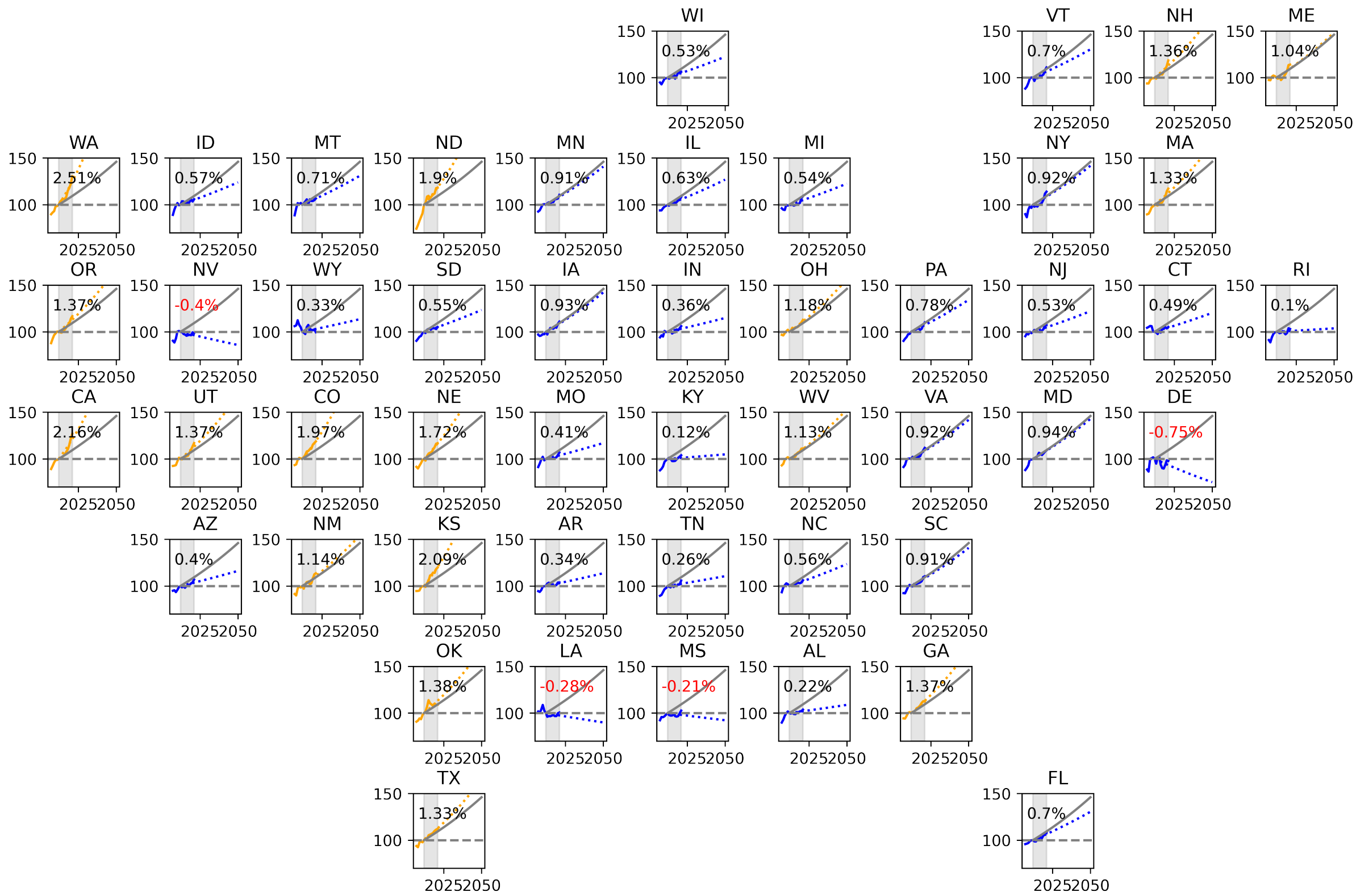

Fig. S21: US state-level labor productivity trends between 2007-2021 with regression between 2012-2021. The productivity values are the Productivity Index (2012=100). The gray line projections assume a 1% annual productivity increase. The gray area highlights the time frame when the non-linear least square regression is conducted. The value shown in the figure is the annual productivity increase calculated from the regression. Negative values marked in red indicate productivity decline. The solid blue or orange lines are historical data and the dotted lines are future productivity projections using the calculated coefficient. Blue lines indicate states with slower growth than the 1% assumption and orange lines indicate faster. Data source: [41] and own calculations.

Tables S29 and S30 compare the effects of the national and state-level productivity increase on the modeled national net employment, respectively. Both assumptions would reduce the results by roughly 25% and show significant differences. Table S31 shows no significant differences while comparing the state-level method with the national method, especially in the emission reduction trajectories. Using the state-level assumption may result in slightly higher employment values. To avoid over-fitting the model with small temporal data sets without delving into the fundamental mechanisms of such productivity changes, we chose the simple 1% national assumption, which shows some robustness compared to a more complex method.

Table S29: Effect of the national productivity assumption on the national net employment in the 2040s compared to the 2020s across emission trajectories.

Emission trajectory	t	p	mean	SD
Current Policies	16.551	3.70E-27	0.729	0.014
Current Policies (-)	17.334	2.09E-28	0.727	0.016
95% by 2050	12.298	6.38E-20	0.728	0.016
95% by 2050 (-)	16.457	4.85E-27	0.725	0.017
100% by 2035	13.271	1.12E-21	0.731	0.017

The (-) indicates the emission trajectories without nascent technologies. The t statistics and p values are calculated from one-sided Welch's t-tests on the national net employment with and without the national productivity assumption. The mean and standard deviations (SD) are from the same two sets of assumptions.

Table S30: Effect of the state-level productivity assumption on the national net employment in the 2040s compared to the 2020s across emission trajectories.

Emission trajectory	t	p	mean	SD
Current Policies	14.676	3.46E-24	0.757	0.013
Current Policies (-)	15.535	1.23E-25	0.753	0.015
95% by 2050	10.965	1.62E-17	0.754	0.015
95% by 2050 (-)	14.975	9.37E-25	0.746	0.018
100% by 2035	12.134	1.25E-19	0.754	0.017

The (-) indicates the emission trajectories without nascent technologies. The t statistics and p values are calculated from one-sided Welch's t-tests on the national net employment with and without the state-level productivity assumption. The mean and standard deviations (SD) are from the same two sets of assumptions.

Table S31: Comparing the effect of the state-level versus national productivity assumption on the national net employment in the 2040s compared to the 2020s across emission trajectories.

Emission trajectory	t	p	mean	SD
Current Policies	-1.956	0.054	1.039	0.021
Current Policies (-)	-1.878	0.064	1.036	0.024
95% by 2050	-1.344	0.183	1.037	0.024
95% by 2050 (-)	-1.438	0.154	1.030	0.028
100% by 2035	-1.288	0.201	1.032	0.028

The (-) indicates the emission trajectories without nascent technologies. The t statistics and p values are calculated from one-sided Welch's t-tests on the national net employment with the state-level and national productivity assumption. The mean and standard deviations (SD) are from the same two sets of assumptions.

6.2 Gender composition

Figure S22 shows the national gender composition of 14 JEDI sectors. The industrial crosswalk from Census sectors to JEDI sectors is in Table S26. Figures S23 and S24 are state-level data in 2010 and 2020 respectively. Because such gender composition remained relatively static in the past decade, we assume that the future gender composition to remain at 2020 levels, especially without any policy interventions.

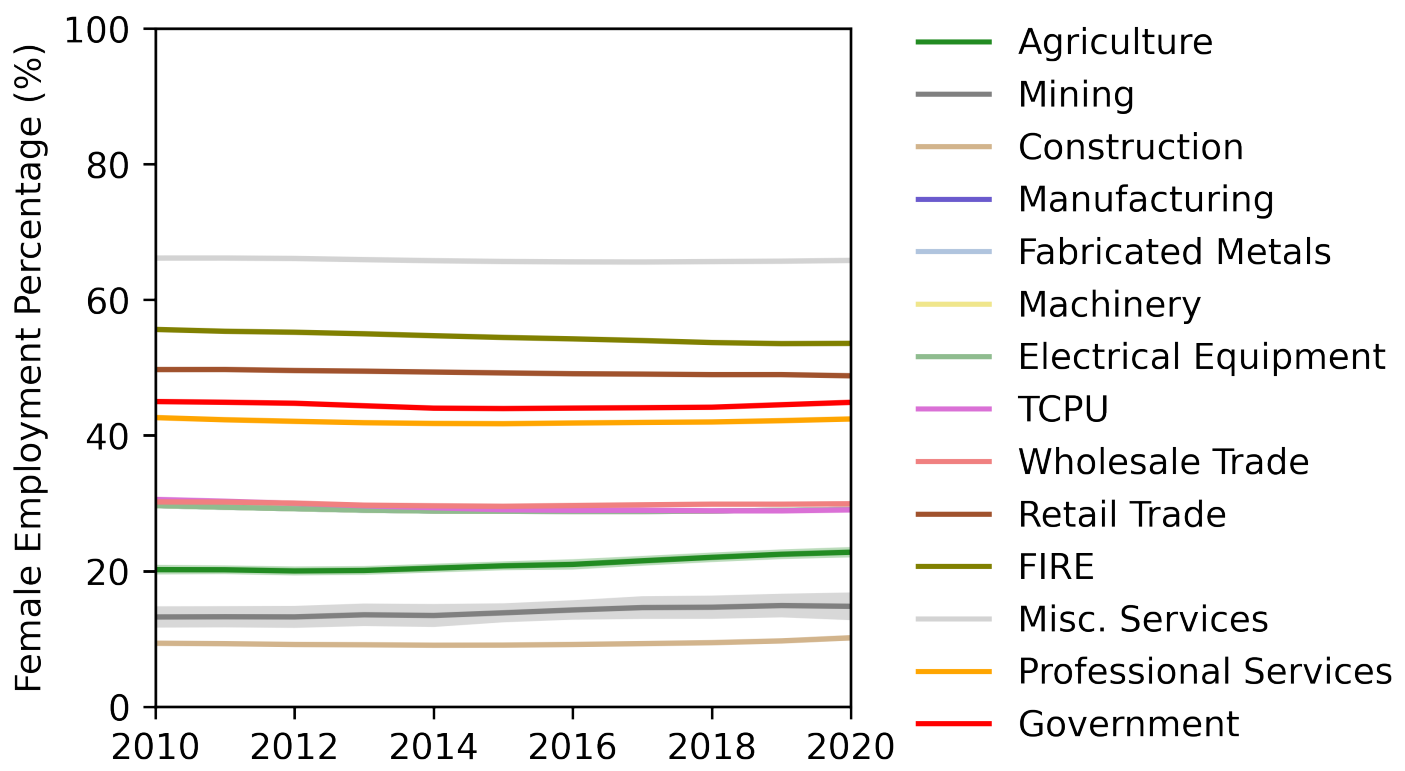

Fig. S22: Trends of US national female employment percentage from 2010 to 2020 across 14 JEDI industry sectors. TCPU stands for Transportation, Communications, and Public Utilities. FIRE stands for Finance, Insurance, and Real Estate. Miscellaneous services include sectors such as education, healthcare, and the arts. Data are presented as the American Community Survey estimates +/- the margins of errors. Data source: [42].

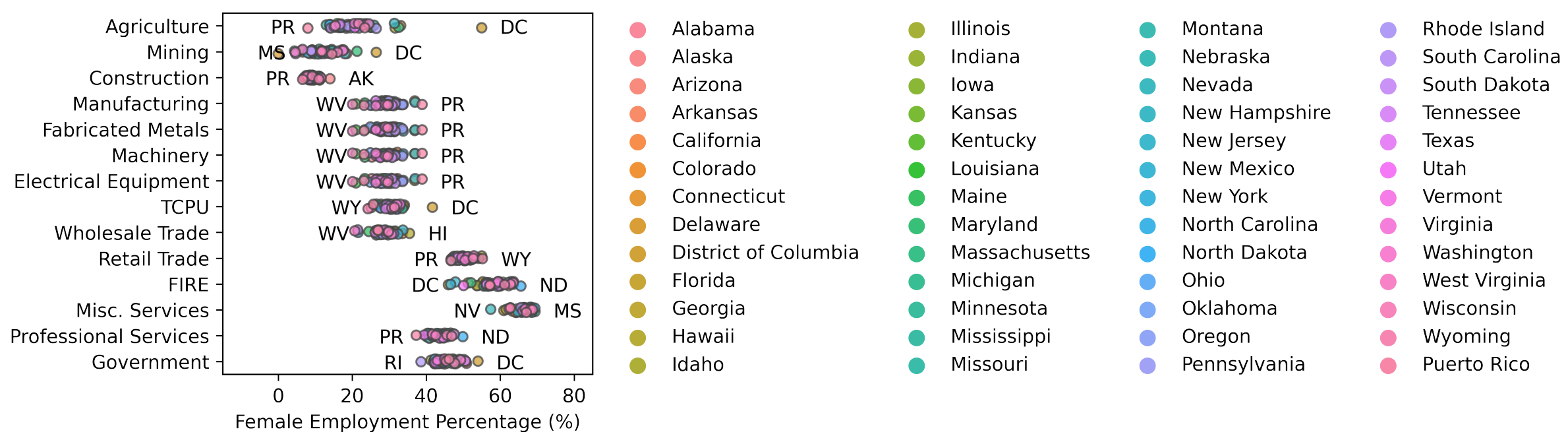

Fig. S23: Female employment percentage in 2010 across 14 JEDI industry sectors and 52 states. The states with the maximum and minimum percentages of each industry sector are denoted. Data source: [42].

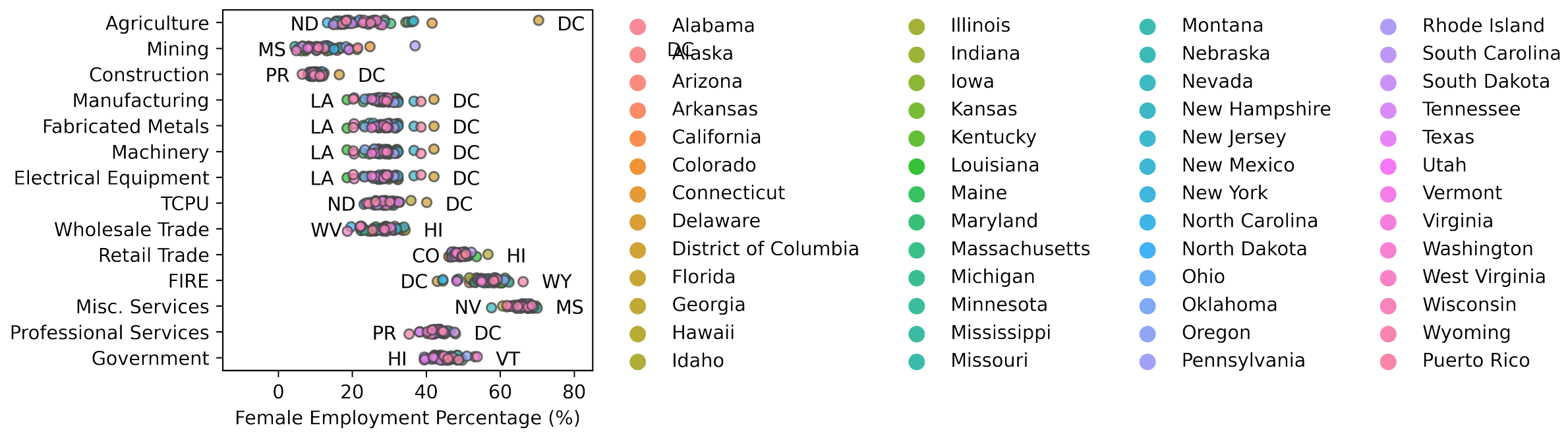

Fig. S24: Female employment percentage in 2020 across 14 JEDI industry sectors and 52 states. The states with the maximum and minimum percentages of each industry sector are denoted. Data source: [42].

6.3 Age distribution

Figure S25 shows the age distribution across JEDI sectors nationally. The variations by industry sector can be large. For example, nearly 20% of the agriculture workforce is 65 years and older, while the compositions in the other sectors are less than 10%. Figure S26 shows the age distribution across states. The variations across the states are roughly at 5%. The industrial crosswalk from BLS to JEDI sectors is in Table S28.

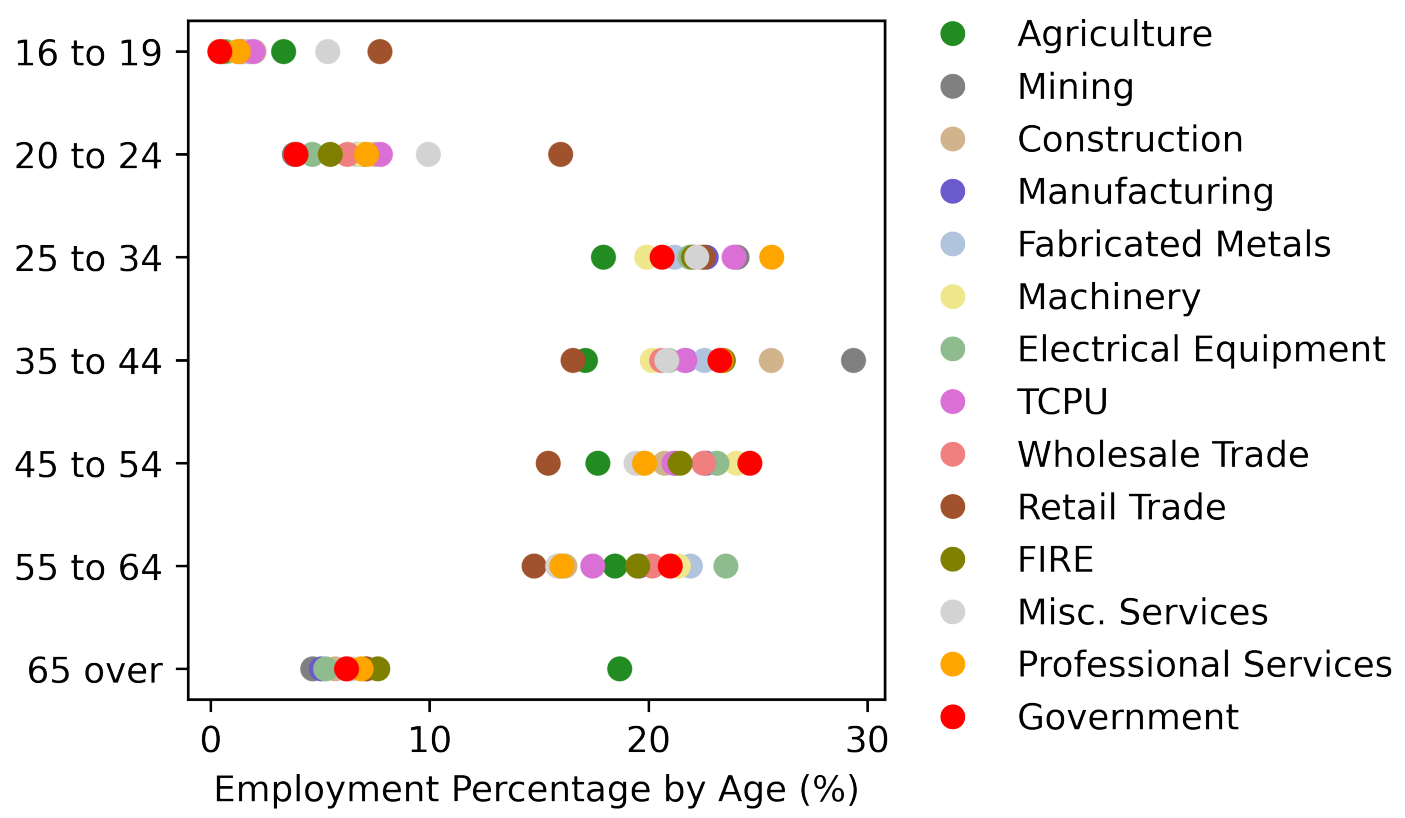

Fig. S25: Employment distribution by age group in 2021 across 14 JEDI industry sectors. Data source: [40] and own calculations.

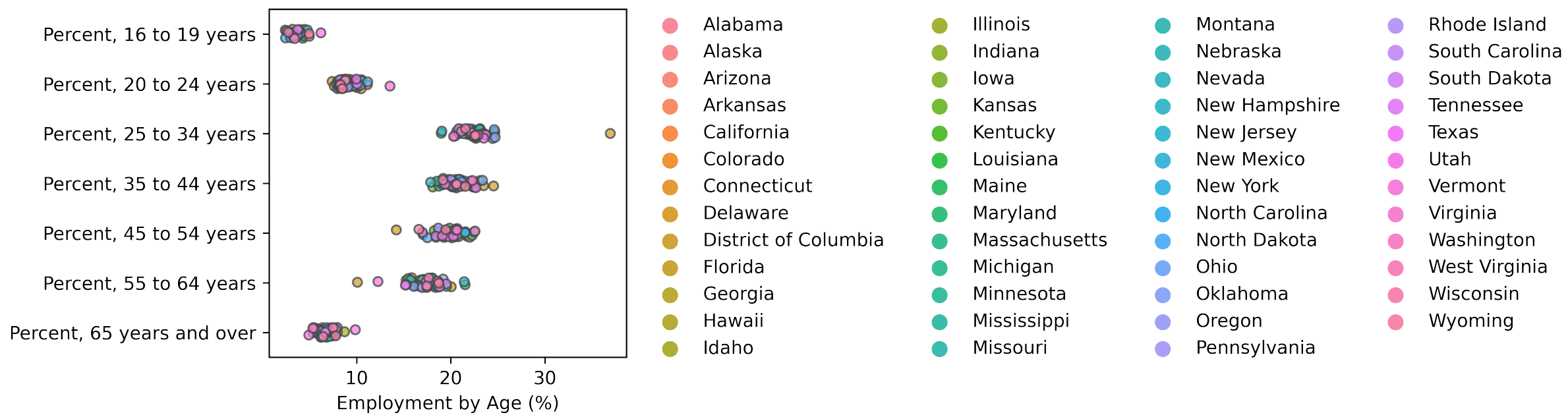

Fig. S26: Employment distribution by age group in 2019 across 52 states. Data source: [43].

6.4 Racial composition

Figure S27 shows the racial composition across 14 JEDI sectors in 2021. The demographic data is from Table 18 "Employed persons by detailed industry, sex, race, and Hispanic or Latino ethnicity" of the Current Population Survey of the BLS [44]. The industry crosswalk from BLS to JEDI sectors is in Table S28. Sectors employing more Asian Americans than the population average include Electrical Equipment, FIRE, Manufacturing, and Misc Services. The sector employing more Black or African Americans than the population average is Government. Sectors employing more Hispanic or Latinx Americans include Agriculture and Construction.

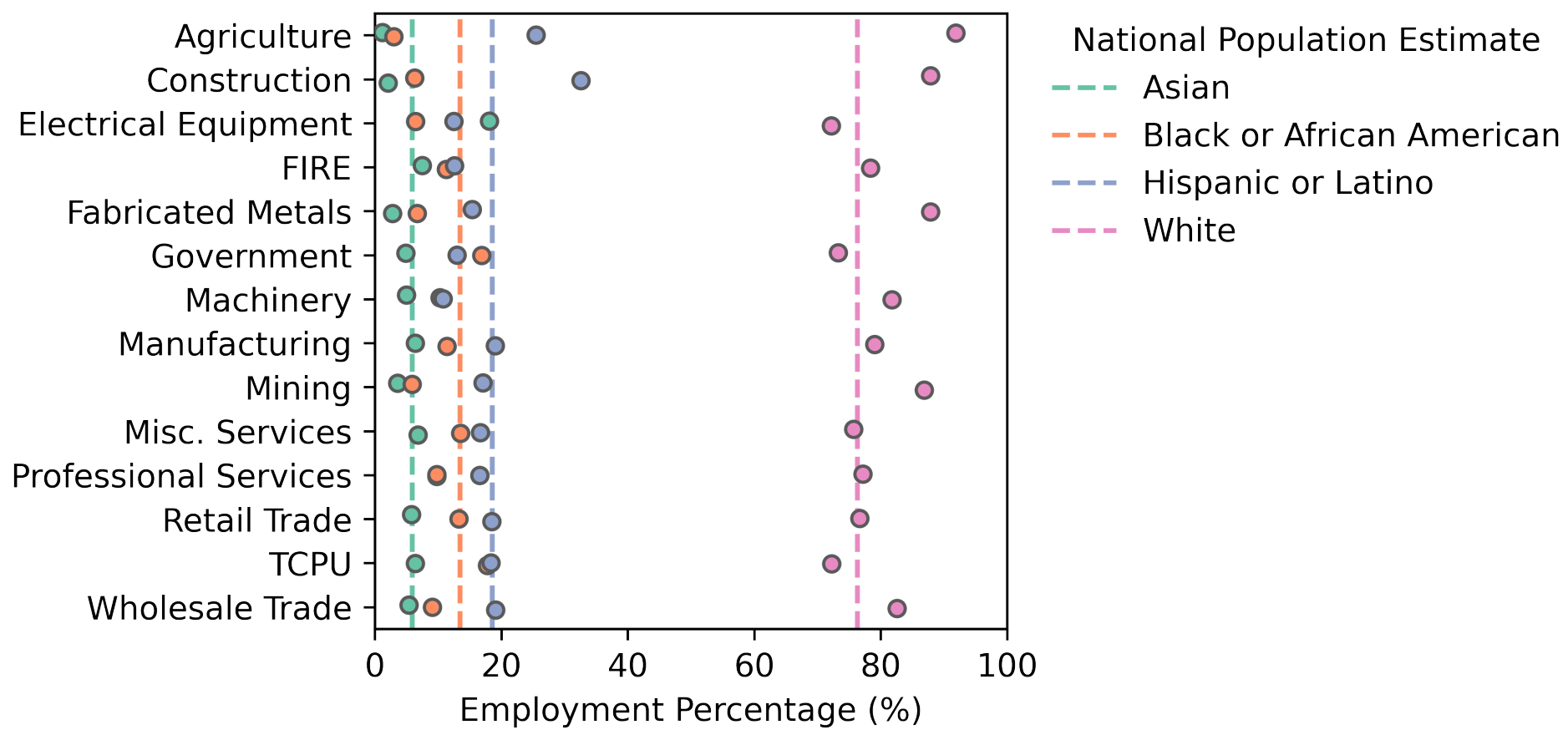

Fig. S27: National employment racial distribution in 2021 across 14 JEDI industry sectors. The dotted vertical lines represent the national population estimates. Data source: [44].

Figure S28 shows historical racial composition data between 2003-2022. Construction workers from Hispanic or Latinx backgrounds have increased the most in the past two decades. The compositions of people of color workers have shown increases in most sectors. Given the complexity of the racial dynamics in the workforce and the relatively large changes in the past, this work does not model or assume any future trends of racial compositions in the power sector. Instead, we discuss the sectoral opportunities created with respect to the 2022 racial composition, acknowledging that it would not be static during the period of transition.

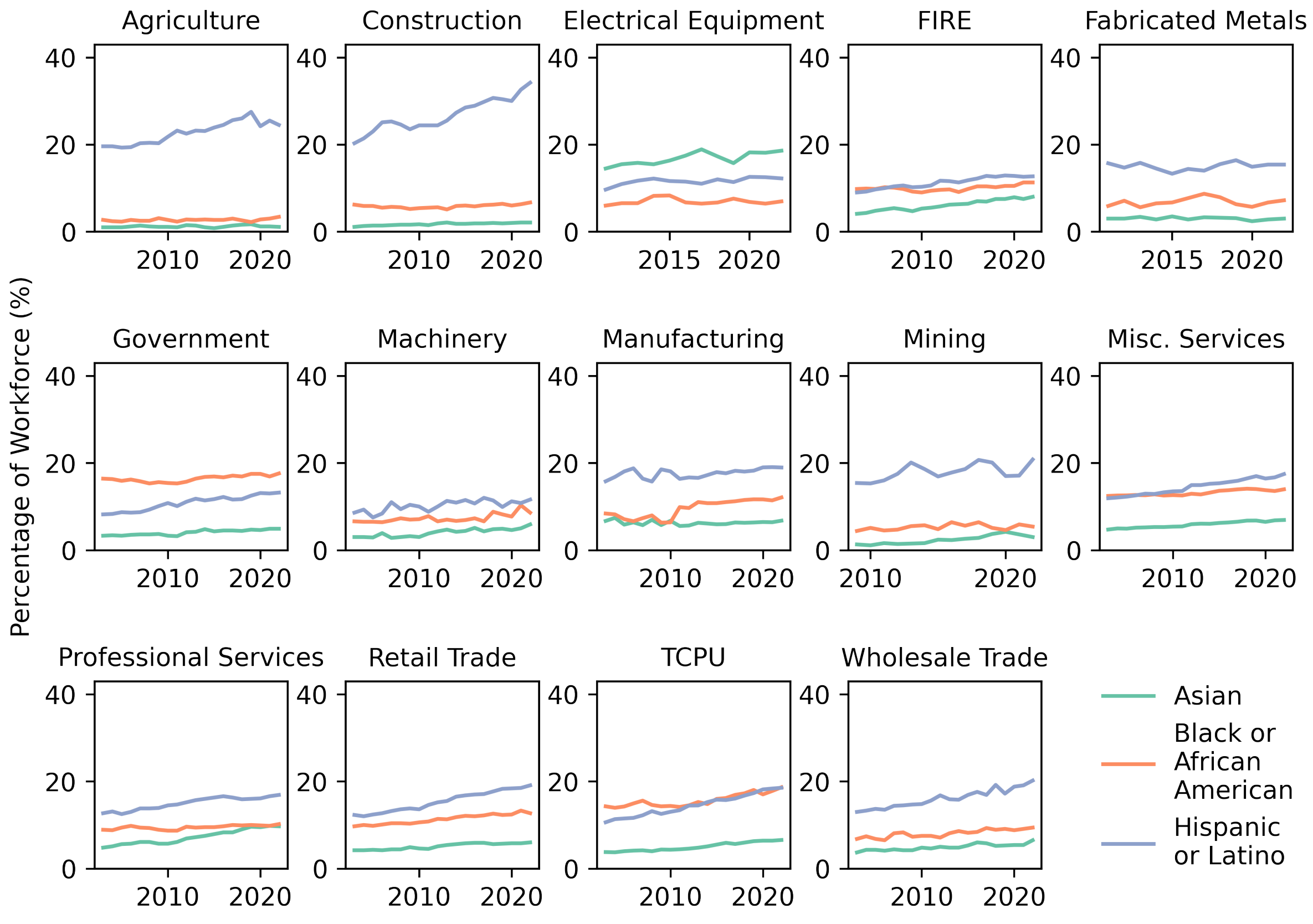

Fig. S28: National employment racial distribution between 2003-2022 across 14 JEDI industry sectors. Data source: [44].

6.5 Job Zones

Figures S29 and S30 show the Job Zone compositions in the 48 continental states across JEDI sectors. The industrial crosswalk from BLS to JEDI sectors used here are in Table S28.

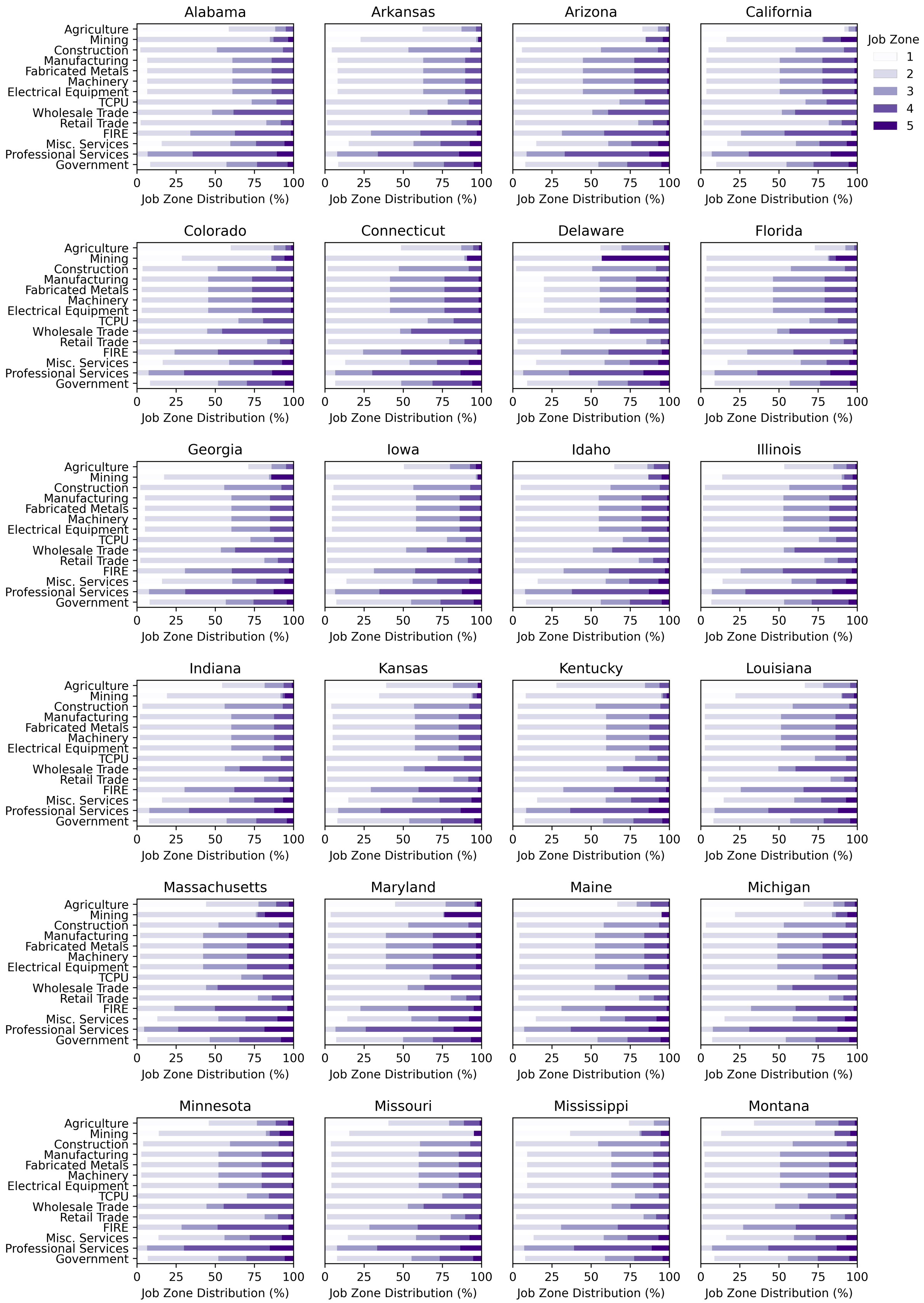

Fig. S29: The compositions of 14 JEDI industry sectors by occupation Job Zones in US States (1 of 2). Data source: [45, 46], and own calculations.

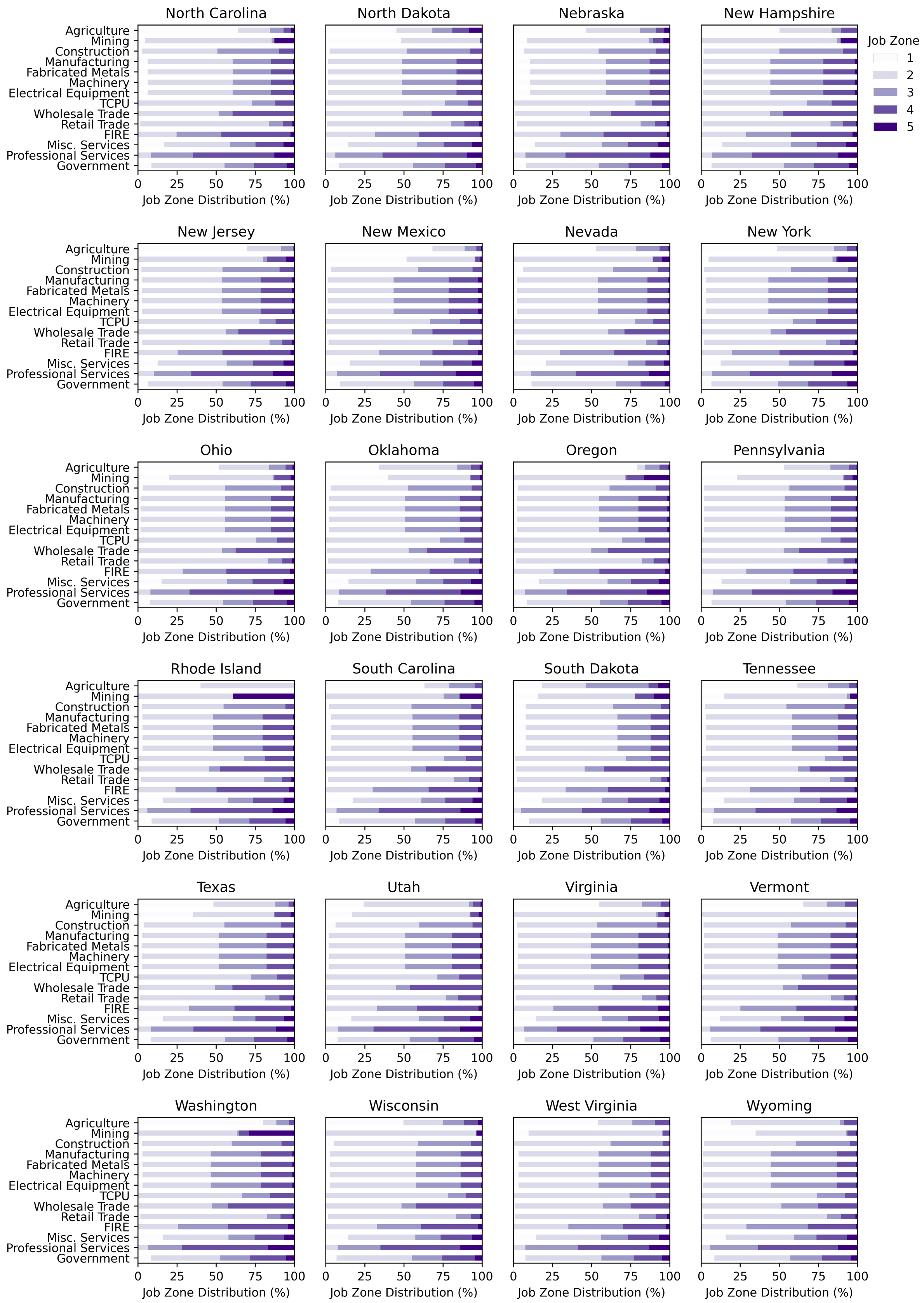

Fig. S30: The compositions of 14 JEDI industry sectors by occupation Job Zones in US States (2 of 2). Data source: [45, 46], and own calculations.

7 Modeled Employment Impacts

7.1 National Impacts

7.1.1 Aggregate Results

Tables S32 and S33 show the national Employment Change in the 2040s compared to the 2020s and the Normalized Employment Change (normalized to the size of the electricity sector in 2022), respectively. Under absolute terms, the 100% by 2035 and 95% reduction by 2050 without nascent technology trajectories show the most significant differences compared to the Current Policies trajectory. The 100% by 2035 trajectory demonstrates slightly more jobs created than the Current Policies, while the other trajectories demonstrate fewer jobs. Table **??** shows the absolute employment across emission trajectories.

Table S32: National Employment Change in the 2040s compared to the 2020s across emission trajectories.

Emission trajectory	Mid case	min	max	t	p	mean	SD
Current Policies	458.37	384.51	577.26				
Current Policies (-)	447.38	367.30	568.40	1.277	0.205	0.974	0.025
95% by 2050	452.89	370.28	582.75	0.096	0.924	0.996	0.051
95% by 2050 (-)	437.63	369.53	566.01	1.832	0.071	0.961	0.033
100% by 2035	438.81	346.69	554.26	0.847	0.400	0.980	0.049

The (-) indicates the emission trajectories without nascent technologies. The units are in thousand jobs. The t statistics and p values are calculated from one-sided Welch's t-tests on the indicated trajectory and Current Policies. The mean and standard deviations (SD) are based on the comparison between the indicated trajectory and Current Policies.

Table S33: National Normalized Employment Change in the 2040s compared to the 2020s across emission trajectories.

Emission trajectory	Mid case	min	max	t	p	mean	SD
Current Policies	0.77	0.66	0.94				
Current Policies (-)	0.74	0.64	0.94	0.933	0.354	0.982	0.026
95% by 2050	0.75	0.61	1.02	0.292	0.771	0.991	0.073
95% by 2050 (-)	0.73	0.62	0.91	2.792	0.007	0.944	0.025
100% by 2035	0.72	0.58	0.90	1.841	0.070	0.959	0.043

The (-) indicates the emission trajectories without nascent technologies. The units are in thousand jobs. The t statistics and p values are calculated from one sides Welch's t-tests on the indicated trajectory and Current Policies. The mean and standard deviations (SD) are based on the comparison between the indicated trajectory and Current Policies.

Table S34: National employment across emission trajectories.

	2022			2050		
**Emission trajectory**	**Mid case**	**min**	**max**	**Mid case**	**min**	**max**
NNP	597.36	557.24	627.88	615.32	539.53	762.83
NNP (-)	600.88	557.24	627.83	596.19	513.62	750.23
95% by 2050	601.72	560.69	628.33	596.57	503.54	840.48
95% by 2050 (-)	598.55	589.12	628.32	580.97	527.70	752.12
100% by 2035	612.82	594.72	626.71	595.45	496.22	724.18

The (-) indicates the emission trajectories without nascent technologies. The units are in thousand jobs.

7.1.2 Technology-level Results

Table S35 shows the national employment by technology in the 2040s across three emission reduction trajectories. Bioenergy CCS was not deployed under Current Policies and renewable fuel was not deployed under Current Policies and 95% by 2050, thus not showing employment in those cases. The offshore wind deployment is constant across trajectories, thus generating the same number of jobs. Table S36 shows Welch's t-test statistics comparing emission reduction trajectories and Current Policies. More battery, hydro, natural gas CCS, pumped storage, transmission, and onshore wind jobs are generated in the 100% by 2035 trajectory. Less natural gas and coal jobs are created in the same trajectory. Other technologies such as solar do not show significant differences across trajectories.

7.2 State-level Impacts

Tables S37 and S38 show the modeled employment under the Mid case 100% reduction by 2035 scenario, which is the trajectory with the most employment expansion. Compared to historical energy employment data compiled by the Department of Energy [47], the modeled electricity system jobs are in the same magnitudes. The model jobs also include intra-regional transmission, the inter-state trade of fossil fuels, and other supply chain factors. Considering the Bureau of Labor Statistics' employment projection between 2021-2031 [48], most states' modeled electricity system jobs would outpace the overall employment. Since the future employment projection by BLS partially attributed the population and employment slowdown to reduced international migration, immigration policies could fill the gap. Domestic migrations and reskilling of other declining workforce areas may also contribute to new opportunities. Since this work only accounts for electricity system jobs, we cannot specify the mechanisms enabling labor transiency.

Table S35: National employment by technology in the 2040s in three emission trajectories.

	**100% by 2035**			**95% by 2050**			**Current Policies**		
**Technology**	**Mid**	**mean**	**SD**	**Mid**	**mean**	**SD**	**Mid**	**mean**	**SD**
Battery	46.49	43.11	9.93	32.90	32.92	10.51	29.38	28.77	10.62
Bioenergy	13.45	13.47	0.11	13.63	13.54	0.12	13.52	13.47	0.09
Bioenergy CCS	30.27	30.27	0.61	1.45	1.19	0.61	0.00	0.00	0.00
Coal	0.02	0.14	0.37	24.78	27.86	6.57	33.49	38.57	12.67
Coal CCS	0.39	1.04	1.51	7.97	10.33	10.59	5.99	9.79	10.67
Geothermal	2.25	2.31	0.40	2.12	2.28	0.48	2.07	2.34	0.48
RE fuel	9.45	9.57	5.92	0.00	0.00	0.00	0.00	0.00	0.00
Hydro	47.08	46.86	0.73	46.68	46.35	0.79	46.38	45.98	0.69
NG	66.56	70.40	12.37	132.11	119.92	21.24	167.15	161.89	45.22
NG CCS	15.07	22.22	18.09	19.12	15.45	9.46	5.21	5.28	3.62
Nuclear	108.30	108.16	0.38	108.21	102.43	9.45	108.24	98.89	16.82
OGS	0.43	0.44	0.03	3.64	3.87	1.30	3.65	3.89	1.36
Pumped storage	9.38	9.34	5.12	3.26	3.96	3.74	3.00	3.32	2.62
Solar	195.80	186.76	31.37	176.56	180.07	33.07	169.06	167.44	36.66
Transmission	12.58	11.74	3.37	10.82	11.32	2.75	5.84	6.53	1.57
Wind offshore	13.03	13.03	0.00	13.03	13.03	0.00	13.03	13.03	0.00
Wind onshore	66.51	65.60	11.19	51.32	55.69	8.98	45.66	48.00	11.57

The units are in thousand jobs. Mid refers to the outcome under Mid case energy system assumptions. The mean and standard deviations (SD) are calculated from the employment data across energy systems assumptions. CCS means carbon capture and storage. RE fuel means renewable fuels. NG means natural gas. OGS means oil gas steam.

Table S36: T-test statistics on the national employment by technology in the 2040s.

	95% by 2050			100% by 2035		
**Technology**	**t**	**p**	**Sig**	**t**	**p**	**Sig**
Battery	-1.002	0.326		-3.554	0.002	**
Biopower	-1.586	0.127		0.193	0.849	
Coal	2.703	0.015	*	10.929	1.34E-7	***
Coal CCS	-0.130	0.898		2.927	0.012	*
Geothermal	0.306	0.763		0.147	0.885	
Hydro	-1.252	0.223		-3.168	0.004	**
NG	3.029	0.008	**	7.036	6.42E-6	***
NG CCS	-3.623	0.002	**	-3.312	0.006	**
Nuclear	-0.660	0.517		-1.985	0.07	
OGS	0.043	0.966		9.144	9.27E-7	***
Pumped storage	-0.503	0.620		-3.773	0.001	**
Solar	-0.922	0.366		-1.443	0.162	
Transmission	-5.447	2.92E-5	***	-5.039	1.02E-4	***
Wind onshore	-1.890	0.072		-3.940	6.15E-4	***

The t statistics and p values are calculated from one-sided Welch's t-tests on the indicated trajectory and Current Policies. CCS means carbon capture and storage. RE fuel means renewable fuels. NG means natural gas. OGS means oil gas steam.
Significance: *p<0.05; **p<0.01; ***p<0.001

Table S37: Modeled state-level employment under the Mid case 100% reduction by 2035 scenario contextualized by historical energy system employment and future employment projections (Part 1/2).

	**USEER 2021**		**Modeled jobs**				**Projected jobs**
Unit	%	%	thousand jobs		%	%	thousand jobs
State	EPG	Energy	2022	2030	2022	2030	2030
Alabama	0.19	1.29	18.14	23.92	0.36	0.47	18.88 ×
Arizona	0.32	1.14	9.94	12.81	0.14	0.18	10.34 ×
Arkansas	0.12	1.17	4.86	7.1	0.16	0.23	5.06 ×
California	0.44	1.65	40.77	40.83	0.1	0.1	42.43
Colorado	0.39	1.86	14.72	10.06	0.25	0.17	15.32
Connecticut	0.18	1.52	4.93	5.22	0.14	0.14	5.13 ×
Delaware	0.13	1.62	0.55	0.42	0.05	0.04	0.57
Florida	0.22	1.03	26.69	39.6	0.12	0.18	27.78 ×
Georgia	0.15	0.99	12.14	11.4	0.11	0.11	12.63
Idaho	0.11	0.94	2.47	2.5	0.13	0.13	2.57
**Illinois**	0.24	1.37	42.09	42.32	0.33	0.33	43.8
Indiana	0.25	1.4	14.03	12.29	0.21	0.18	14.6
Iowa	0.3	1.45	4.57	6.39	0.14	0.2	4.76 ×
Kansas	0.44	1.83	6.06	8.71	0.21	0.3	6.31 ×
Kentucky	0.1	1.11	30.84	23.38	0.68	0.52	32.1
Louisiana	0.19	2.55	8.29	9.85	0.18	0.21	8.63 ×
Maine	0.23	1.19	1.59	2.1	0.12	0.15	1.65 ×
Maryland	0.24	1.57	7.77	9.33	0.13	0.15	8.09 ×
Massachusetts	0.46	1.97	3.82	5.75	0.05	0.08	3.98 ×
Michigan	0.26	1.3	12.28	15.27	0.12	0.15	12.78 ×
Minnesota	0.26	1.5	8.38	13.05	0.15	0.23	8.72 ×
Mississippi	0.14	1.2	9.53	11.45	0.32	0.39	9.92 ×
Missouri	0.15	1.25	6.67	8.95	0.11	0.15	6.94 ×
**Montana**	0.11	1.73	10.47	6.05	0.95	0.55	10.9

The historical jobs from the U.S. Energy & Employment Jobs Report [47] are calculated as a percentage of the state population in 2021. EPG stands for electric power generation. Energy jobs include EPG, transmission, distribution storage, fuels, energy efficiency, and motor vehicles. The modeled jobs are from this analysis and as percentages of the state population in 2021. The projected jobs in 2030 are calculated from the modeled jobs in 2022, assuming a 0.5% annual workforce expansion [48]. Those marked with a cross (×) indicate that the projected jobs in 2030 do not satisfy the scale of growth modeled in 2030. The states marked in bold are one of the five major coal-producing states.

Table S38: Modeled state-level employment under the Mid case 100% reduction by 2035 scenario contextualized by historical energy system employment and future employment projections (Part 2/2).

	**USEER 2021**		**Modeled jobs**				**Projected jobs**
Unit	%	%	thousand jobs		%	%	thousand jobs
State	EPG	Energy	2022	2030	2022	2030	2030
Nebraska	0.34	1.67	2.99	5.62	0.15	0.29	3.11 ×
Nevada	0.39	1.42	2.69	4.26	0.09	0.14	2.8 ×
New Hampshire	0.4	1.53	2	2.53	0.14	0.18	2.08 ×
New Jersey	0.2	0.92	11.07	10.74	0.12	0.12	11.52
New Mexico	0.24	2.2	4.42	4.07	0.21	0.19	4.6
New York	0.2	1.2	13.92	18.21	0.07	0.09	14.49 ×
North Carolina	0.18	1.18	19.54	24.51	0.19	0.23	20.34 ×
North Dakota	0.38	5.21	2.75	2.61	0.35	0.34	2.86
Ohio	0.21	1.3	16.69	15.14	0.14	0.13	17.37
Oklahoma	0.17	2.47	8.95	12.52	0.22	0.31	9.31 ×
Oregon	0.24	1.54	6.45	7.09	0.15	0.17	6.71 ×
**Pennsylvania**	0.16	1.31	44.16	41.47	0.34	0.32	45.96
Rhode Island	0.24	1.56	0.4	0.82	0.04	0.07	0.42 ×
South Carolina	0.49	1.35	12.76	21.88	0.25	0.42	13.28 ×
South Dakota	0.35	1.71	1.41	2.08	0.16	0.23	1.47 ×
Tennessee	0.17	1.33	11.55	13.6	0.17	0.19	12.02 ×
Texas	0.2	2.17	43.91	51.71	0.15	0.18	45.7 ×
Utah	0.35	1.71	7.15	6.39	0.21	0.19	7.44
Vermont	0.39	2.72	1.05	1	0.16	0.15	1.09
Virginia	0.18	1.34	22.02	18.71	0.25	0.22	22.92
Washington	0.18	1.36	12.06	13.4	0.16	0.17	12.55 ×
**West Virginia**	0.17	3.24	15.42	10.17	0.86	0.57	16.05
Wisconsin	0.18	1.5	7.96	10.79	0.14	0.18	8.28 ×
**Wyoming**	0.23	5.76	39.92	11.01	6.9	1.9	41.55

The historical jobs from the U.S. Energy & Employment Jobs Report [47] are calculated as a percentage of the state population in 2021. EPG stands for electric power generation. Energy jobs include EPG, transmission, distribution storage, fuels, energy efficiency, and motor vehicles. The modeled jobs are from this analysis and as percentages of the state population in 2021. The projected jobs in 2030 are calculated from the modeled jobs in 2022, assuming a 0.5% annual workforce expansion [48]. Those marked with a cross (×) indicate that the projected jobs in 2030 do not satisfy the scale of growth modeled in 2030. The states marked in bold are one of the five major coal-producing states.

Figure S31 shows the distributions of state-level employment change across all model runs by the NNP and 95% by 2050 emission trajectories. Employment change here indicates new opportunities created (positive) or reskilling needs (negative) in the 2040s compared to the size of the state-electricity workforce in 2020s.

7.3 Sector- and Skill-based Impacts

Figures S32-S34 are heatmaps showing the Normalized Unit Employment Change under the three core emission reduction trajectories. TCPU stands for Transportation, Communications, and Public Utilities. FIRE stands for Finance, Insurance, and Real Estate. Miscellaneous services include sectors such as education, healthcare, and the arts.

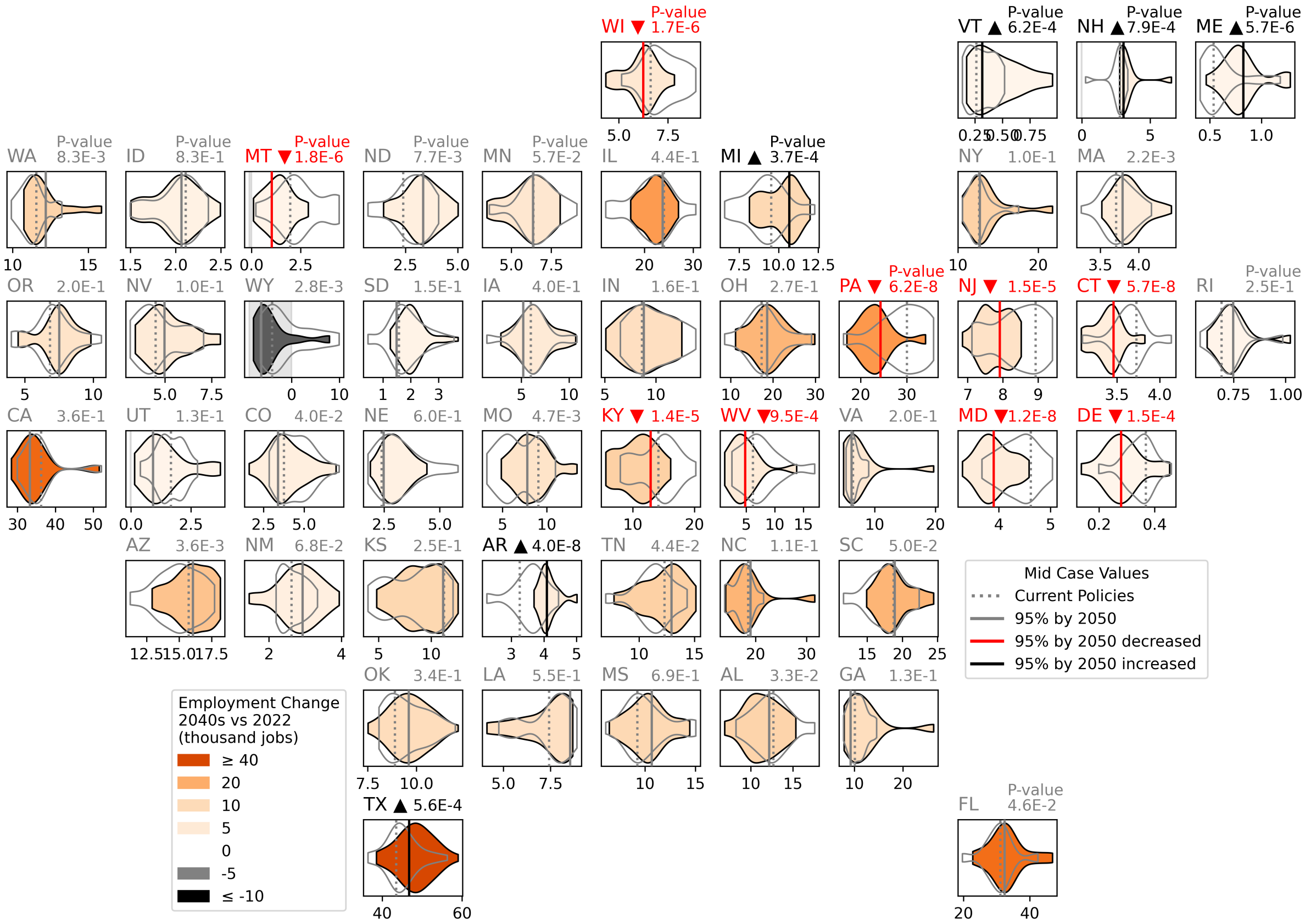

Fig. S31: State-level Employment Change under the 100% by 2035 emission trajectory. Employment Change (Equation 7) describes the difference in the workforce in the decade before mid-century compared to today while accounting for natural retirement. The empty violin plots (minima to maxima) with gray outlines represent the results from all systems assumptions under Current Policies (n=39); the dotted gray lines represent the mid-case system assumption. The filled violin plots represent all results from the 100% by 2035 trajectory (n=39); the solid lines represent their mid-case system assumption. The gray line means the emission reduction shows no significant difference from Current Policies (P>0.001) across system assumptions. The red lines indicate that the emission reduction trajectory leads to significantly fewer jobs (P<0.001); the black lines indicate significantly more jobs (P<0.001). The state labels are marked accordingly with black upward and red downward arrows. The P values to the right of state labels are calculated from one-sided Welch's t-tests. The negative areas are shaded gray.

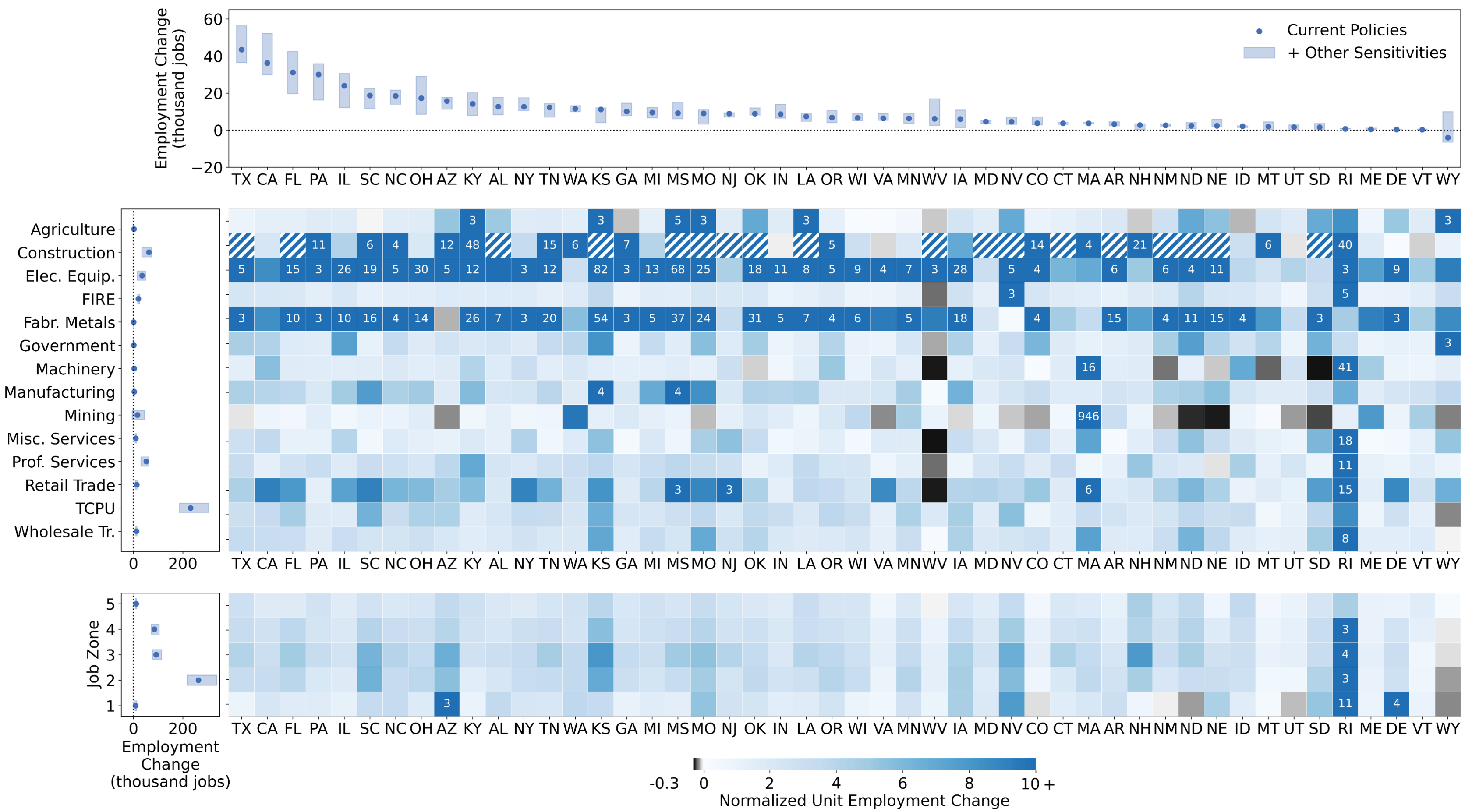

Fig. S32: Distribution of Employment Change and Normalized Unit Employment Change in the Current Policies trajectory between the 2020s and 2040s. The bar graphs show the Employment Change (in thousand jobs) of the 2040s compared to the 2020s aggregated by state (top), economic sector (middle left), and job zone (bottom left) across all power sector transition scenarios. In the top panel, the scatters represent the mid-case, and the transparent bars represent the full suite of energy system assumptions. States are sorted in descending order of their state-level employment growth. Heatmaps represent the disaggregated employment change normalized to the state's electricity system job numbers (in percentage) in 2022 in the mid-case scenario. The Normalized Unit Employment Change shows the magnitudes of employment growth (blue color scheme) and job losses (gray color scheme). Cells with hatches are units with small 2020s starting values.

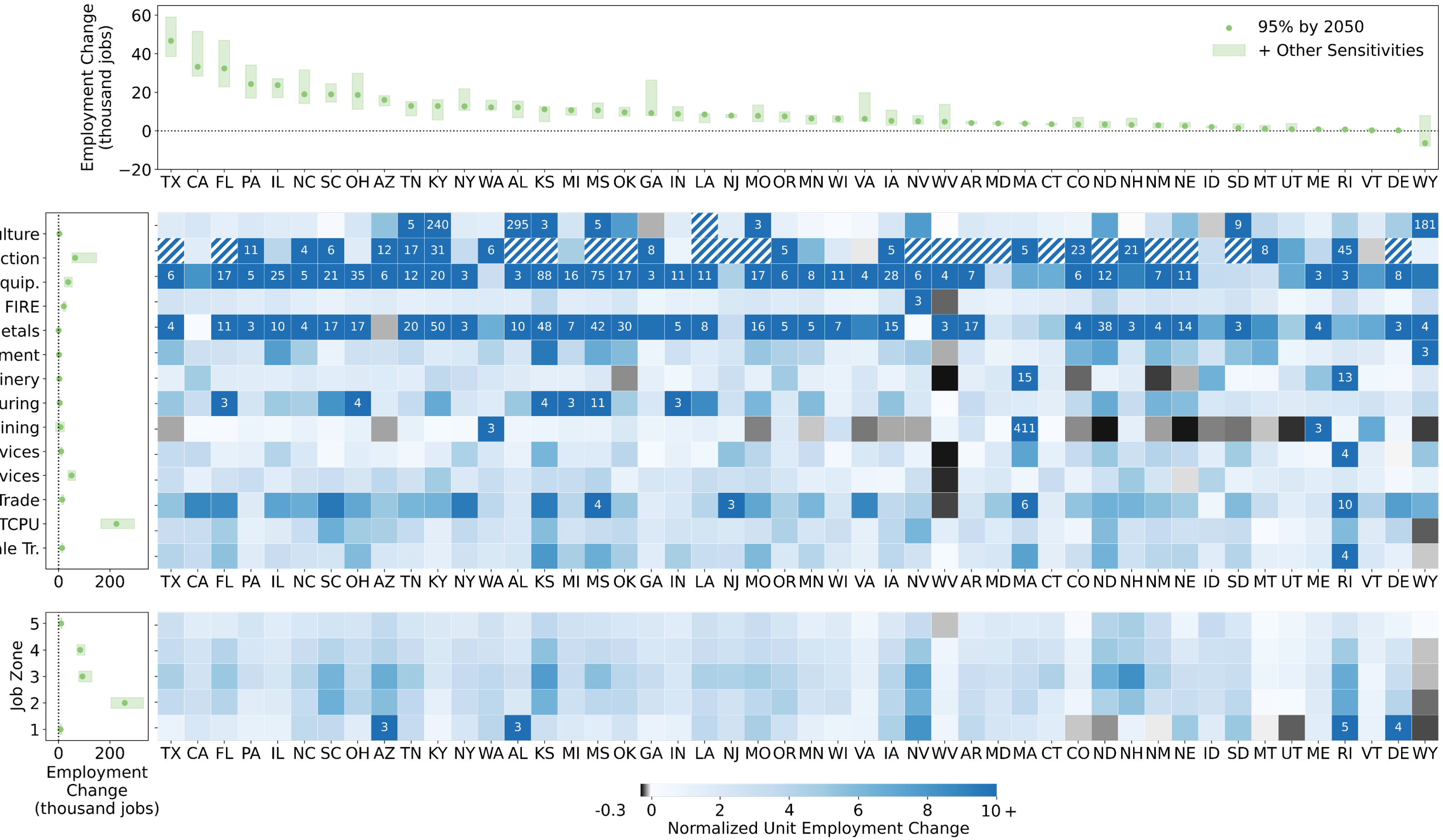

Fig. S33: Distribution of Employment Change and Normalized Unit Employment Change in the 95% by 2050 trajectory between the 2020s and 2040s. The bar graphs show the Employment Change (in thousand jobs) of the 2040s compared to the 2020s aggregated by state (top), economic sector (middle left), and job zone (bottom left) across all power sector transition scenarios. In the top panel, the scatters represent the mid-case, and the transparent bars represent the full suite of energy system assumptions. States are sorted in descending order of their state-level employment growth. Heatmaps represent the disaggregated employment change normalized to the state's electricity system job numbers (in percentage) in 2022 in the mid-case scenario. The Normalized Unit Employment Change shows the magnitudes of employment growth (blue color scheme) and job losses (gray color scheme). Cells with hatches are units with small 2020s starting values.

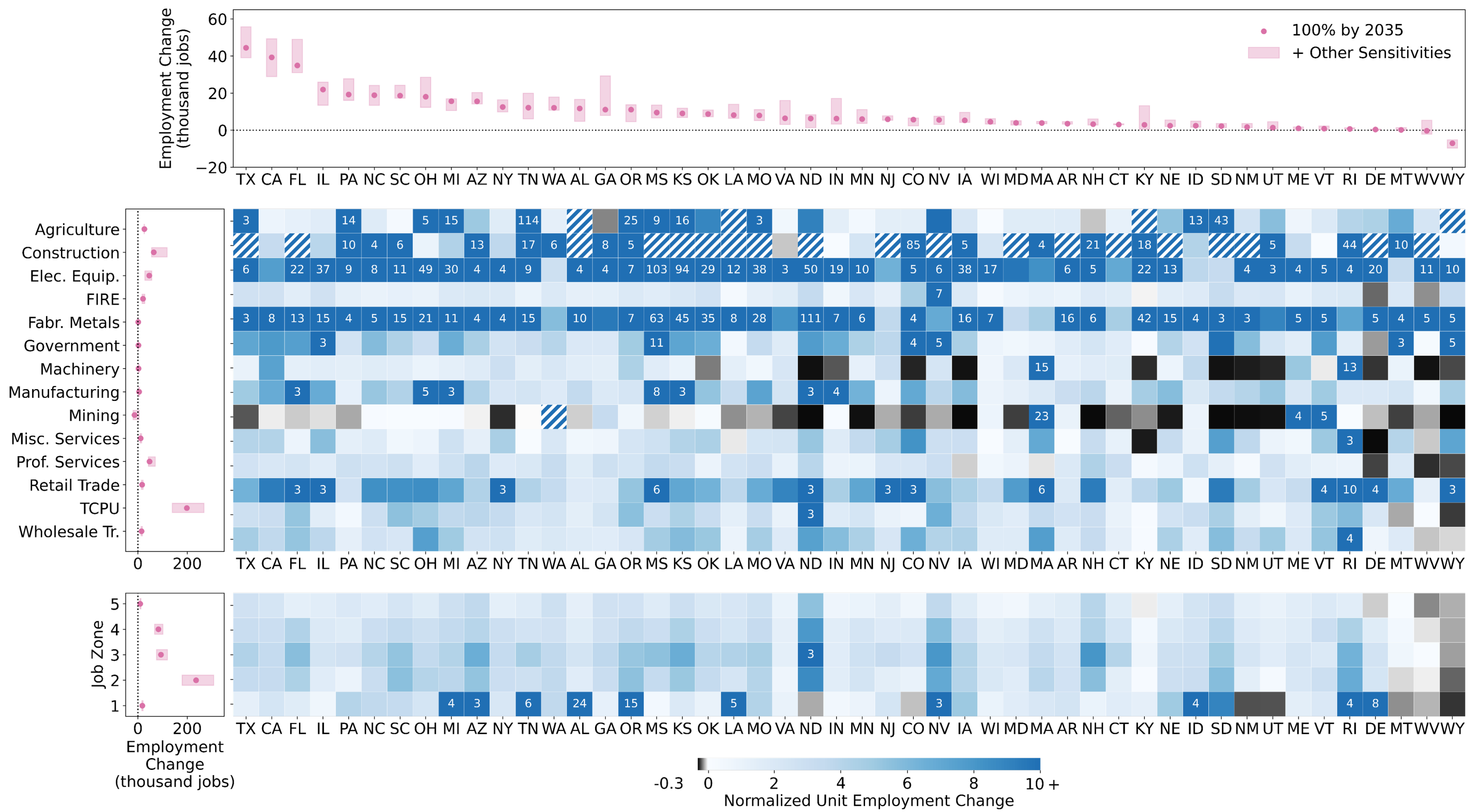

Fig. S34: Distribution of Employment Change and Normalized Unit Employment Change in the 100% by 2035 trajectory between the 2020s and 2040s. The bar graphs show the Employment Change (in thousand jobs) of the 2040s compared to the 2020s aggregated by state (top), economic sector (middle left), and job zone (bottom left) across all power sector transition scenarios. In the top panel, the scatters represent the mid-case, and the transparent bars represent the full suite of energy system assumptions. States are sorted in descending order of their state-level employment growth. Heatmaps represent the disaggregated employment change normalized to the state's electricity system job numbers (in percentage) in 2022 in the mid-case scenario. The Normalized Unit Employment Change shows the magnitudes of employment growth (blue color scheme) and job losses (gray color scheme). Cells with hatches are units with small 2020s starting values.

Supplementary References

[1] NREL. Scenario Viewer: Data Downloader, 2021.

[2] Pieter Gagnon, Maxwell Brown, Dan Steinberg, Patrick Brown, Sarah Awara, Vincent Carag, Stuart Cohen, Wesley Cole, Jonathan Ho, Sarah Inskeep, Nate Lee, Trieu Mai, Matthew Mowers, Caitlin Murphy, and Brian Sergi. 2022 Standard Scenarios Report: A U. S. Electricity Sector Outlook. Technical Report December, 2022.

[3] Jonathan Ho, Jonathon Becker, Maxwell Brown, Patrick Brown, Ilya Chernyakhovskiy, Stuart Cohen, Wesley Cole, Sean Corcoran, Kelly Eurek, Will Frazier, Pieter Gagnon, Nathaniel Gates, Daniel Greer, Paige Jadun, Saroj Khanal, Scott Machen, Madeline Macmillan, Trieu Mai, Matthew Mowers, Caitlin Murphy, Amy Rose, Anna Schleifer, Brian Sergi, Daniel Steinberg, Yinong Sun, and Ella Zhou. Regional Energy Deployment System (ReEDS) Model Documentation: Version 2020. Technical Report June, 2021.

[4] Climeworks. Orca: the first large-scale plant, 2021.

[5] U.S Energy Information Administration. Form EIA-860 detailed data with previous form data (EIA-860A/860B), 2020.

[6] Max Wei, Shana Patadia, and Daniel M. Kammen. Putting renewables and energy efficiency to work: How many jobs can the clean energy industry generate in the US? *Energy Policy*, 38(2):919–931, 2010.

[7] Jay Rutovitz, Elsa Dominish, and Jenni Downes. Calculating Global Energy Sector Jobs - Methodology Update 2015. Technical report, 2015.

[8] C. R. Kenley, R. D. Klingler, C. M. Plowman, R. Soto, R. J. Turk, R. L. Baker, S. A. Close, V. L. McDonnell, S. W. Paul, L. R. Rabideau, S. S. Rao, and B. P. Reilly. Job creation due to nuclear power resurgence in the United States. *Energy Policy*, 37(11):4894–4900, 2009.

[9] NEI. The Impact of Exelon's Nuclear Fleet on the Illinois Economy. Technical Report October, 2014.

[10] NEI. Economic Impacts of The Davis-Besse Nuclear Power Station. Technical Report January, 2015.

[11] NEI. Economic Impacts of The R.E. Ginna Nuclear Power Plant. Technical Report February, 2015.

[12] NEI. The Economic Benefits of Texas ' Nuclear Power Plants. Technical Report December, 2015.

[13] Nuclear Energy Institute. Economic Impacts of the Millstone Power Station. Technical Report January, 2017.

[14] NEI. The Impact of Xcel Energy's Nuclear Fleet on the Minnesota Economy. Technical Report April, 2017.

[15] NEI. Economic Impacts of the Columbia Generating Station. Technical Report January, 2018.

[16] NEI. Economic Impacts of the Cooper Nuclear Station. Technical Report February, 2018.

[17] U.S Energy Information Administration. Capital Cost and Performance Characteristic Estimates for Utility Scale Electric Power Generating Technologies. Technical Report February, 2020.

[18] U.S. Energy Information Administration. Table 3. Energy Prices by Sector and Source, 2020.

[19] U.S. Energy Information Administration. Where our uranium comes from, 2021.

[20] U.S. Energy Information Administration. Petroleum Supply Annual, Volume 1, 2020.

[21] U.S. Energy Information Administration. Oil and petroleum products explained, 2021.

[22] U.S. Energy Information Administration. How much coal, natural gas, or petroleum is used to generate a kilowatthour of electricity?

[23] U.S. Energy Information Administration. Energy conversion calculators, 2021.

[24] Kyle Buchheit, Eric Lewis, Kishore Mahbubani, and Derrick Carlson. Technoeconomic and Life Cycle Analysis of Bio-Energy With Carbon Capture and Storage (BECCS) Baseline. *National Energy Technology Laboratory*, 2021.

[25] Melissae Fellet and Venkat Srinivasan. Will the Li-ion battery industry follow solar photovoltaic's lead? *MRS Bulletin*, 41(9):658–659, 2016.

[26] Kendall Mongird, Vilayanur Viswanathan, Jan Alam, Charlie Vartanian, Vincent Sprenkle, and Richard Baxter. 2020 Grid Energy Storage Technology Cost and Performance Assessment. Technical Report December, 2020.

[27] Jon Runyon. Hydrogen Power Generation – Challenges and Prospects for 100 Hydrogen Gas Turbines. 2020.

[28] Jeffrey Goldmeer and John Catillaz. Hydrogen for power generation: Experience, requirements, and implications for use in gas turbines. Technical report, 2022.

[29] James Vicker, David Peterson, and Katie Randolph. Cost of Electrolytic Hydrogen Production with Existing Technology. Technical report, 2020.

[30] David W. Keith, Geoffrey Holmes, David St. Angelo, and Kenton Heidel. A Process for Capturing CO2 from the Atmosphere. *Joule*, 2(8):1573–1594, 2018.

[31] Mahdi Fasihi, Olga Efimova, and Christian Breyer. Techno-economic assessment of CO 2 direct air capture plants. *Journal of Cleaner Production*, 224:957–980, 2019.

[32] U.S. Energy Information Administration. Coal Data Browser, 2020.

[33] U.S. Energy Information Administration. Natural Gas Annual.

[34] US Census Bureau. The 2017 Commodity Flow Survey Final Tables, 2017.

[35] National Renewable Energy Laboratory. Biopower Atlas.

[36] Matthew Langholtz, Bryce Stokes, and Laurence Eaton. 2016 billion-ton report: Advancing domestic resources for a thriving bioeconomy. Technical report, 2016.

[37] R.L. Bain, W.A. Amos, M. Downing, and R.L. Perlack. Biopower Technical Assessment: State of the Industry and the Technology. *NREL Technical Report*, (NREL/TP-510-33123):200–205, 2003.

[38] Wesley Cole, Vincent J Carag, Maxwell Brown, Patrick Brown, Stuart Cohen, Kelly Eurek, Will Frazier, Pieter Gagnon, Nick Grue, Jonathan Ho, Anthony Lopez, Trieu Mai, Matthew Mowers, Caitlin Murphy, Brian Sergi, Dan Steinberg, and Travis Williams. 2021 Standard Scenarios Report: A U.S. Electricity Sector Outlook. Technical Report November, 2021.

[39] Jeffrey Sachs, Elena Crete, Fiona Laird, Cheyenne Maddox, Caroline Fox, Lauren Barredo, Giovanni Bruna, Emily High, Alainna Lynch, Leila Fanaeian, and Joseph Bettles. America's Zero Carbon Action Plan. 2020.

[40] U.S. Bureau of Labor Statistics. 18b. Employed persons by detailed industry and age, 2021.

[41] U.S. Bureau of Labor Statistics. Data Tables, 2022.

[42] U.S. Census Bureau. S2403 Industry by sex for the civilian employed population 16 years and over, 2019.

[43] U.S. Bureau of Labor Statistics. Expanded State Employment Status Demographic Data, 2019.

[44] U.S. Bureau of Labor Statistics. Labor Force Statistics from the Current Population Survey, 2022.

[45] O*NET Online. Browse by Job Zone, 2021.

[46] U.S. Bureau of Labor Statistics. Occupational Employment and Wage Statistics.

[47] US Department of Energy Office of Policy. U.S. Energy Employment Jobs Report (USEER), 2021.

[48] U.S. Bureau of Labor Statistics. Employment Projections 2021-2031. Technical report, 2022.
